# Supplementary material for: Trityl Cation-Catalyzed Hosomi-Sakurai Reaction of Allylsilane with β,γ-Unsaturated α-Ketoester to Form γ,γ-Disubstituted α-Ketoesters
Source: Molecules. 2022 Jul 24;27(15):4730. doi: 10.3390/molecules27154730 (PMC9331905; doi:10.3390/molecules27154730)
Supplement: Supplementary file 1 [file molecules-27-04730-s001.zip › molecules-1826160-supplementary.pdf]

Trityl Cation-catalyzed Hosomi-Sakurai Reaction of Allylsilane with β,γ-Unsaturated α-Ketoester to Form γ, γ-Disubstituted α-Ketoesters

Zubao Gan, Deyun Cui, Hongyun Zhang, Ying Feng, Liying Huang, Yingying Gui, Lu Gao* and Zhenlei Song*

Key Laboratory of Drug-Targeting and Drug Delivery System of the Education Ministry and Sichuan Province, Sichuan Engineering Laboratory for Plant-Sourced Drug and Sichuan Research Center for Drug Precision Industrial Technology, West China School of Pharmacy, Sichuan University, Chengdu 610041, China

E-mail: lugao@scu.edu.cn; zhenleisong@scu.edu.cn

Supporting Information

Table of Contents

1. General Methods ……………………………………………………………………………….S2

2. General Procedure and Spectral Data of Products………………………………….S2-S17

2.1. Preparations and Spectral Data of 3……………………………………………….S2-S14

2.2. Preparations and Spectral Data of 5……………………………………………….S14-S16

2.3. Control Experiments………. ………………………………………………………S16-S19

3. Copies of NMR spectra ………………………………………………………………...S20-S76

1. General Methods

TLC was performed on glass-backed silica plates and visualized using UV, $KMnO_4$ stains, $H_3PO_4 \cdot 12MoO_3$/EtOH stains, H_2SO_4(conc.)/anisaldehyde/EtOH stains. Column chromatography was performed using silica gel (300-400 mesh) eluting with EtOAc/petroleum ether. ^{1}H-NMR spectra were recorded at 400 MHz (Varian) or 600 M Hz (Agilent) and ^{13}C-NMR spectra were recorded at 100 MHz or 150 M Hz (Agilent) using $CDCl_3$ (except where noted) with TMS or residual solvent as standard. Infrared spectra were obtained using KCl plates on a VECTOR22. High-resolution mass spectral analyses were performed on Waters Q-TOF Premier at State Key Laboratory of Biotherapy, West China Hospital, Sichuan University. In each case, diastereoselective ratio was determined by ^{1}H-NMR spectra were recorded at 400 M Hz (Varian) or 600 M Hz (Agilent). CH_2Cl_2, Et_3N were distilled from CaH_2. All spectral data obtained for new compounds are reported here.

2. General Procedure and Spectral Data of Products

2.1. Preparations and Spectral Data of 3

***Preparation of* 3a**

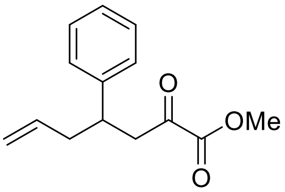

3a

3a: To a solution of β,γ-unsaturated α-ketoester **1a** (20 mg, 0.11 mmol) and allyltrimethylsilane **2a** (21 μL, 0.13 mmol) and in anhyd. CH_2Cl_2 (2 mL) under argon atmosphere was added $[(Ph_3C)[BPh(^F)_4]]$ (1 mg, 0.11×10^{-2} mmol) at 25 °C. After stirring for 10 minutes, the reaction was quenched with *p*-TsOH (0.5 M in MeOH, 0.1 mL). The mixture was directly concentrated under reduced pressure. Purification of the crude residue via silica gel flash column chromatography (gradient eluent: 0-2.0% of EtOAc /petroleum ether) afforded **3a** (25 mg, 97%) as a colorless oil. ^{1}H NMR (400 MHz, $CDCl_3$) δ 2.37 (dd, 1H, J_1 = 7.2 Hz, J_2 = 14.0 Hz), 2.45 (dd, 1H, J_1 = 7.2 Hz, J_2 = 14.0 Hz), 3.19 (d, 2H, J = 7.2 Hz), 3.34 (dddd, 1H, J = 7.2 Hz), 3.80 (s, 3H), 5.00 (d, 1H, J = 8.0 Hz), 5.03 (d, 1H, J = 15.2 Hz), 5.66 (m, 1H), 7.20 (m, 3H), 7.28 (d, 1H, J = 3.6 Hz), 7.31 (d, 1H, J = 7.6 Hz); ^{13}C NMR (100 MHz, $CDCl_3$) δ 40.2, 40.8, 44.9, 52.9, 117.3, 126.7, 127.5, 128.5, 135.8, 143.2,

161.3, 192.9; IR (neat) cm^{-1} 3029, 2954, 2923, 1731, 1442, 1277, 1253, 1089, 919; HRMS (MALDI, m/z) calcd for $C_{14}H_{16}O_3Na$ $(M+Na)^+$: 255.0992, found 255.0989.

Preparation of 3b

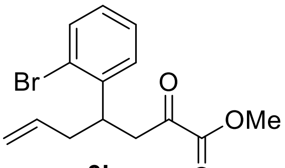

3b

3b: Using the same procedure as that used for **3a**. (*E*)-methyl 4-(2-bromophenyl)-2-oxobut-3-enoate (28 mg, 0.11 mmol) and **2a** (21 μL, 0.13 mmol) in anhyd. CH_2Cl_2 (2 mL) under argon atmosphere with $[(Ph_3C)[BPh(^F)_4]]$ (1 mg, 0.11 × 10^{-2} mmol) at 25 °C for 10 minutes afforded **3b** (34 mg, 98%) as a colorless oil. ^1H NMR (400 MHz, $CDCl_3$) δ 2.37 (dd, 1H, J_1 = 7.2 Hz, J_2 = 14.0 Hz), 2.46 (dd, 1H, J_1 = 7.2 Hz, J_2 = 13.6 Hz), 3.15 (dd, 1H, J_1 = 7.2 Hz, J_2 = 17.6 Hz), 3.26 (dd, 1H, J_1 = 7.2 Hz, J_2 = 18.0 Hz), 3.82 (s, 3H), 3.92 (dddd, 1H, *J* = 7.2 Hz), 5.01 (d, 1H, *J* = 9.2 Hz), 5.02 (d, 1H, *J* = 18.0 Hz), 5.68 (m, 1H), 7.06 (t, 1H, *J* = 7.2 Hz), 7.20 (d, 1H, *J* = 7.2 Hz), 7.27 (t, 1H, *J* = 7.2 Hz), 7.55 (d, 1H, *J* = 8.0 Hz) ; ^{13}C NMR (400 MHz, $CDCl_3$) δ 38.0, 39.3, 43.6, 53.0, 117.4, 126.2, 127.4, 128.5, 130.8, 131.7, 135.6, 137.0, 161.5, 193.2; IR (neat) cm^{-1} 2954, 2853, 1730, 1640, 1440, 1267, 1066, 917; HRMS (MALDI, m/z) calcd for $C_{14}H_{15}BrO_3Na$ $(M+Na)^+$: 333.0097, found 333.0095.

Preparation of 3c

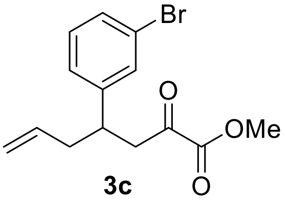

3c

3c: Using the same procedure as that used for **3a**. (*E*)-methyl 4-(3-bromophenyl)-2-oxobut-3-enoate (28 mg, 0.11 mmol) and **2a** (21 μL, 0.13 mmol) in anhyd. CH_2Cl_2 (2 mL) under argon atmosphere with $[(Ph_3C)[BPh(^F)_4]]$ (1 mg, 0.11 × 10^{-2} mmol) at 25 °C for 10 minutes afforded **3c** (34 mg, 98%) as a colorless oil. ^1H NMR (400 MHz, $CDCl_3$) δ 2.34 (dd, 1H, J_1 = 7.2 Hz, J_2 = 13.6 Hz), 2.41 (dd, 1H, J_1 = 7.2 Hz, J_2 = 14.0 Hz), 3.17 (d, 2H, *J* = 7.6 Hz), 3.30 (dddd, 1H, *J* = 7.2 Hz), 3.82 (s, 3H), 5.00 (d, 1H, *J* = 10.0 Hz), 5.03 (d, 1H, *J* = 16.8 Hz), 5.61 (m, 1H), 7.15 (m, 2H), 7.33 (d, 1H, *J* = 8.8

Hz); ^{13}C NMR (400 MHz, $CDCl_3$) δ 37.5, 38.4, 42.8, 52.0, 116.6, 123.8, 126.6, 126.9, 127.1, 132.2, 134.2, 140.9, 160.2, 191.3; IR (neat) cm^{-1} 2956, 2923, 2852, 1731, 1470, 1438, 1261, 1074, 1023, 919; HRMS (MALDI, m/z) calcd for $C_{14}H_{15}BrO_3Na$ $(M+Na)^+$: 333.0097, found 333.0093.

Preparation of 3d

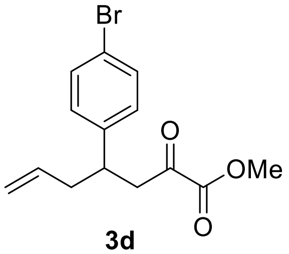

3d

3d: Using the same procedure as that used for **3a**. (*E*)-methyl 4-(4-bromophenyl)-2-oxobut-3-enoate (28 mg, 0.11 mmol) and **2a** (21 μL, 0.13 mmol) in anhyd. CH_2Cl_2 (2 mL) under argon atmosphere with $[(Ph_3C)[BPh(^F)_4]]$ (1 mg, 0.11 × 10^{-2} mmol) at 25 °C for 10 minutes afforded **3d** (33 mg, 97%) as a colorless oil. ^{1}H NMR (400 MHz, $CDCl_3$) δ 2.34 (dd, 1H, J_1 = 7.2 Hz, J_2 = 14.4 Hz), 2.39 (dd, 1H, J_1 = 7.2 Hz, J_2 = 15.2 Hz), 3.16 (d, 2H, J = 9.2 Hz), 3.30 (dddd, 1H, J = 7.2 Hz), 3.81 (s, 3H), 5.00 (d, 1H, J = 11.2 Hz), 5.01 (d, 1H, J = 16.2 Hz), 5.61 (m, 1H), 7.08 (d, 2H, J = 8.0 Hz), 7.41 (d, 1H, J = 8.4 Hz); ^{13}C NMR (100 MHz, $CDCl_3$) δ 39.6, 40.6, 44.7, 117.6, 120.4, 129.3, 131.6, 135.3, 142.2, 161.1, 192.5; IR (neat) cm^{-1} 2953, 2851, 1731, 1488, 1439, 1406, 1276, 1072, 1010, 918, 822; HRMS (MALDI, m/z) calcd for $C_{14}H_{15}BrO_3Na$ $(M+Na)^+$: 333.0097, found 333.0103.

Preparation of 3e

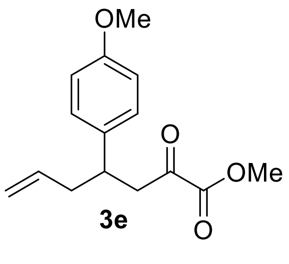

3e

3e: Using the same procedure as that used for **3a**. (*E*)-methyl 4-(4-methoxyphenyl)-2-oxobut-3-enoate (23 mg, 0.11 mmol) and **2a** (21 μL, 0.13 mmol) in anhyd. CH_2Cl_2 (2 mL) under argon atmosphere with $[(Ph_3C)[BPh(^F)_4]]$ (1 mg, 0.11 × 10^{-2} mmol) at 25 °C for 10 minutes afforded **3e** (26 mg, 90%, H-S:D-A = 90:10) as a colorless oil. ^{1}H NMR (400 MHz, $CDCl_3$) δ 2.34 (dd, 1H, J_1 = 7.2 Hz, J_2 = 13.6 Hz), 2.41 (dd, 1H, J_1 = 6.8 Hz, J_2 = 14.0 Hz), 3.15 (d, 2H, J = 6.8 Hz), 3.29 (dddd, 1H,

J = 7.2 Hz), 3.77 (s, 3H), 3.79 (s, 3H), 4.99 (d, 1H, J = 8.4 Hz), 5.01 (d, 1H, J = 16.8 Hz), 5.65 (m, 1H), 6.82 (d, 2H, J = 8.4 Hz), 7.11 (d, 1H, J = 8.4 Hz); ^{13}C NMR (400 MHz, $CDCl_3$) δ 39.5, 40.9, 45.1, 52.9, 55.2, 113.8, 117.1, 128.4, 135.2, 135.9, 158.2, 161.3, 193.0 ; IR (neat) cm^{-1} 3001, 2925, 2840, 1729, 1611, 1512, 1441, 1250, 1179, 1072, 1035(m), 918, 829; HRMS (MALDI, m/z) calcd for $C_{15}H_{18}O_4Na$ $(M+Na)^+$: 285.1097, found 285.1099.

Preparation of 3f

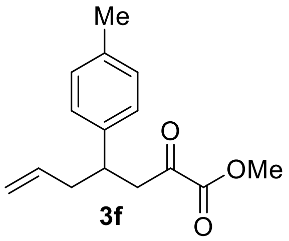

3f: Using the same procedure as that used for **3a**. (*E*)-methyl 4-(4-methylphenyl)-2-oxobut-3-enoate (22 mg, 0.11 mmol) and **2a** (21 μL, 0.13 mmol) in anhyd. CH_2Cl_2 (2 mL) under argon atmosphere with $[(Ph_3C)[BPh(^F)_4]]$ (1 mg, 0.11 × 10^{-2} mmol) at 25 °C for 10 minutes afforded **3f** (26 mg, 95%, H-S:D-A = 95:5) as a colorless oil. ^{1}H NMR (400 MHz, $CDCl_3$) δ 2.30 (s, 3H), 2.34 (dd, 1H, J_1 = 7.2 Hz, J_2 = 14.4 Hz), 2.42 (dd, 1H, J_1 = 7.2 Hz, J_2 = 14.4 Hz), 3.16 (d, 2H, J = 6.8 Hz), 3.30 (dddd, 1H, J = 7.2 Hz), 3.79 (s, 3H), 4.98 (d, 1H, J = 8.4 Hz), 5.02 (d, 1H, J = 15.6 Hz), 5.65 (m, 1H), 7.09 (m, 4H); ^{13}C NMR (400 MHz, $CDCl_3$) δ 21.0, 39.8, 40.8, 45.0, 52.9, 117.2, 127.3, 129.2, 136.0, 136.2, 140.2, 161.3, 193.0; IR (neat) cm^{-1} 2922, 2734, 1731, 1641, 1514, 1440, 1276, 1072, 917; HRMS (MALDI, m/z) calcd for $C_{15}H_{18}O_3Na$ $(M+Na)^+$: 269.1148, found 269.1147.

Preparation of 3g

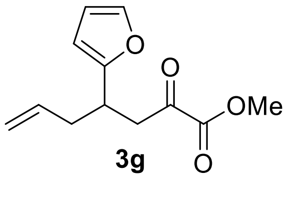

3g: Using the same procedure as that used for **3a**. Methyl (*E*)-4-(furan-2-yl)-2-oxobut-3-enoate (19 mg, 0.11 mmol) and **2a** (21 μL, 0.13 mmol) in anhyd. CH_2Cl_2 (2 mL) under argon atmosphere with $[(Ph_3C)[BPh(^F)_4]]$ (1 mg, 0.11 × 10^{-2} mmol) at 25 °C for 10 minutes afforded **3g** (24 mg, 97%) as a colorless oil. ^{1}H NMR (400 MHz, $CDCl_3$) δ 2.37 (ddd, 1H, $J_1 = J_2 =$ 7.2 Hz, J_3 = 14.0 Hz), 2.49 (ddd,

1H, $J_1 = J_2 =$ 6.8 Hz, J_3 = 14.0 Hz), 3.10 (dd, 1H, J_1 = 6.4 Hz, J_2 = 18.0 Hz), 3.20 (dd, 1H, J_1 = 7.6 Hz, J_2 = 17.6 Hz), 3.47 (dddd, 1H, J = 7.2 Hz), 3.84 (s, 3H), 5.04 (d, 1H, J = 10.0 Hz), 5.04 (d, 1H, J = 17.2 Hz), 5.69 (m, 1H), 6.03 (d, 1H, J = 3.2 Hz), 6.25 (dd, 1H, J_1 = 2.0 Hz, J_2 = 3.2 Hz), 7.29 (d, 1H, J = 1.2 Hz); ^{13}C NMR (400 MHz, $CDCl_3$) δ 33.6, 37.9, 42.3, 53.0, 105.5, 110.0, 117.6, 135.2, 141.3, 156.1, 161.1, 192.5; IR (neat) cm^{-1} 2924, 2853, 1731, 1505, 1442, 1259, 1070, 1011, 917, 803; HRMS (MALDI, m/z) calcd for $C_{12}H_{14}O_4Na$ $(M+Na)^+$: 245.0784, found 245.0783.

<u>Preparation of 3h</u>

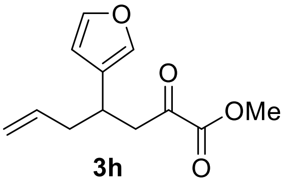

3h

3h: Using the same procedure as that used for **3a**. Methyl (*E*)-4-(furan-3-yl)-2-oxobut-3-enoate (19 mg, 0.11 mmol) and **2a** (21 μL, 0.13 mmol) in anhyd. CH_2Cl_2 (2 mL) under argon atmosphere with [$(Ph_3C)[BPh(^F)_4]$] (1 mg, 0.11 × 10^{-2} mmol) at 25 °C for 10 minutes afforded **3h** (24 mg, 97%) as a colorless oil. ^{1}H NMR (400 MHz, $CDCl_3$) δ 2.35 (m, 2H), 3.05 (dd, 1H, J_1 = 7.6 Hz, J_2 = 18.4 Hz), 3.10 (dd, 1H, J_1 = 6.4 Hz, J_2 = 18.4 Hz), 3.30 (dddd, 1H, J = 7.2 Hz), 3.84 (s, 3H), 5.03 (d, 1H, J = 10.4 Hz), 5.04 (d, 1H, J = 16.4 Hz), 5.70 (m, 1H), 6.28 (s, 1H), 7.25 (s, 1H), 7.34 (s, 1H); ^{13}C NMR (400 MHz, $CDCl_3$) δ 30.7, 39.8, 44.4, 53.0, 109.4, 117.5, 127.0, 135.6, 139.0, 143.1, 161.3, 192.9; IR (neat) cm^{-1} 2924, 2854, 1731, 1641, 1441, 1261, 1160, 1068, 1029, 920, 873, 789; HRMS (MALDI, m/z) calcd for $C_{12}H_{14}O_4Na$ $(M+Na)^+$: 245.0784, found 245.0779.

<u>Preparation of 3i</u>

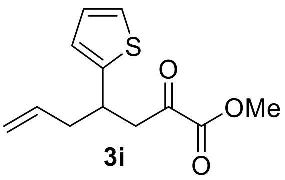

3i

3i: Using the same procedure as that used for **3a**. Methyl (*E*)-2-oxo-4-(thiophen-2-yl)but-3-enoate (21 mg, 0.11 mmol) and **2a** (21 μL, 0.13 mmol) in anhyd. CH_2Cl_2 (2 mL) under argon atmosphere with [$(Ph_3C)[BPh(^F)_4]$] (1 mg, 0.11 × 10^{-2} mmol) at 25 °C for 10 minutes afforded **3i** (26 mg, 98%) as a colorless oil. ^{1}H NMR (400 MHz, $CDCl_3$) δ 2.46 (m, 2H), 3.21 (d, 1H, J = 7.2 Hz), 3.69 (dddd,

1H, J = 6.8 Hz), 3.83 (s, 3H), 5.05 (d, 1H, J = 9.2 Hz), 5.06 (d, 1H, J = 18.4 Hz), 5.71 (m, 1H), 6.83 (d, 1H, J = 2.4 Hz), 6.90 (dd, 1H, J_1 = 3.2, J_2 =3.6 Hz), 7.14 (dd, 1H, J_1 = 0.8 Hz, J_2 = 5.2 Hz); ^{13}C NMR (400 MHz, $CDCl_3$) δ 35.5, 41.5, 45.7, 53.0, 117.8, 123.4, 124.2, 126.6, 135.3, 146.9, 161.1, 192.4; IR (neat) cm^{-1} 2924, 2853, 1731, 1641, 1439, 1260, 1069, 920; HRMS (MALDI, m/z) calcd for $C_{12}H_{14}O_3SNa$ $(M+Na)^+$: 261.0556, found 261.0551.

Preparation of 3j

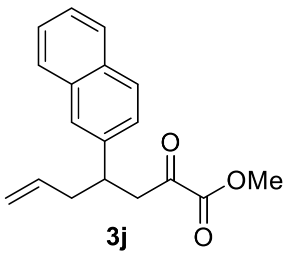

3j

3j: Using the same procedure as that used for **3a**. Methyl 4-(naphthalen-2-yl)-2-oxohept-6-enoate (25 mg, 0.11 mmol) and **2a** (21 μL, 0.13 mmol) in anhyd. CH_2Cl_2 (2 mL) under argon atmosphere with [(Ph_3C)[BPh(^{F})$_4$]] (1 mg, 0.11 × 10^{-2} mmol) at 25 °C for 10 minutes afforded **3j** (30 mg, 96%) as a colorless oil. ^{1}H NMR (400 MHz, $CDCl_3$) δ 2.45 (dd, 1H, J_1 = 7.2 Hz, J_2 = 14.0 Hz), 2.53 (dd, 1H, J_1 = 7.2 Hz, J_2 = 14.0 Hz), 3.25 (dd, 1H, J_1 = 6.8 Hz, J_2 = 13.6 Hz), 3.31 (dd, 1H, J_1 = 7.2 Hz, J_2 = 13.6 Hz), 3.51 (dddd, 1H, J = 7.2 Hz), 3.77 (s, 3H), 4.99 (d, 1H, J = 10.4 Hz), 5.04 (d, 1H, J = 18.0 Hz), 5.67 (m, 1H), 7.35 (d, 1H, J = 8.0 Hz), 7.44 (m, 1H), 7.63 (s, 1H), 7.79 (d, 3H, J = 8.0 Hz) ; ^{13}C NMR (400 MHz, $CDCl_3$) δ 40.4, 40.7, 44.9, 52.9, 117.3, 125.5, 125.7, 126.0, 126.1, 127.6, 127.7, 128.3, 132.4, 133.4, 135.7, 140.6, 161.2, 192.8; IR (neat) cm^{-1} 3008, 2924, 2852, 1730, 1638, 1439, 1260, 1073; HRMS (MALDI, m/z) calcd for $C_{18}H_{18}O_3Na$ $(M+Na)^+$: 305.1148, found 305.1151.

Preparation of 3k

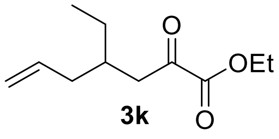

3k

3k: Using the same procedure as that used for **3a**. Ethyl (E)-2-oxohex-3-enoate (17 mg, 0.11 mmol) and **2a** (21 μL, 0.13 mmol) in anhyd. CH_2Cl_2 (2 mL) under argon atmosphere with [(Ph_3C)[BPh(^{F})$_4$]] (1 mg, 0.11 × 10^{-2} mmol) at 25 °C for 10 minutes afforded **3k** (21 mg, 96%) as a colorless oil. ^{1}H

NMR (400 MHz, $CDCl_3$) δ 0.88 (t, 3H, J = 7.2 Hz), 1.30-1.41 (m, 5H), 2.02 (m, 2H), 2.14 (m, 1H), 2.71 (dd, 1H, J_1 = 6.4 Hz, J_2 = 17.6 Hz), 2.79 (dd, 1H, J_1 = 6.4 Hz, J_2 = 17.6 Hz), 4.30 (q, 2H, J = 7.2 Hz), 5.01 (d, 1H, J = 14.8 Hz), 5.71 (m, 1H) ; ^{13}C NMR (400 MHz, $CDCl_3$) δ 11.0, 14.0, 26.4, 34.9, 37.8, 42.9, 62.3, 117.0, 136.3, 161.3, 194.7; IR (neat) cm^{-1} 2966, 2926, 1728, 1640, 1253, 1060, 916; HRMS (MALDI, m/z) calcd for $C_{11}H_{18}O_3Na$ $(M+Na)^+$: 221.1148, found 221.1140.

Preparation of 3l

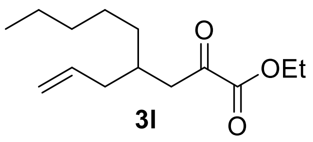

3l

3l: Using the same procedure as that used for **3a**. Ethyl (*E*)-2-oxonon-3-enoate (42 mg, 0.21 mmol) and **2a** (42 μL, 0.26 mmol) in anhyd. CH_2Cl_2 (4 mL) under argon atmosphere with $[(Ph_3C)[BPh(^F)_4]]$ (2 mg, 0.22×10^{-2} mmol) at 25 °C for 10 minutes afforded **3l** (46 mg, 94%) as a colorless oil. ^{1}H NMR (400 MHz, $CDCl_3$) δ 0.88 (t, 3H, J = 7.2 Hz), 1.27 (m, 8H), 2.00 (dddd, 1H, J = 6.8 Hz), 2.09-2.19 (br, 2H), 2.71 (dd, 1H, J_1 = 6.0 Hz, J_2 = 17.2 Hz), 2.81 (dd, 1H, J_1 = 6.4 Hz, J_2 = 17.2 Hz), 4.31 (q, 2H, J = 7.2 Hz), 5.00 (d, 1H, J = 4.8 Hz), 5.02 (d, 1H, J = 16.0 Hz) ; ^{13}C NMR (400 MHz, $CDCl_3$) δ 14.0, 22.5, 26.4, 29.7, 31.9, 33.4, 33.8, 38.3, 43.3, 62.3, 117.0, 136.3, 161.3, 194.7; IR (neat) cm^{-1} 2958, 2926, 2856, 1728, 1463, 1258, 1061, 915; HRMS (MALDI, m/z) calcd for $C_{14}H_{24}O_3Na$ $(M+Na)^+$: 263.1618, found 263.1618.

Preparation of 3m

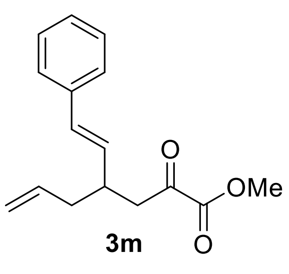

3m

3m: Using the same procedure as that used for **3a**. Ethyl (3*E*,5*E*)-2-oxo-6-phenylhexa-3,5-dienoate (23 mg, 0.11 mmol) and **2a** (21 μL, 0.13 mmol) in anhyd. CH_2Cl_2 (2 mL) under argon atmosphere with $[(Ph_3C)[BPh(^F)_4]]$ (1 mg, 0.11×10^{-2} mmol) at 25 °C for 10 minutes afforded **3m** (23 mg, 80%) as a colorless oil. ^{1}H NMR (400 MHz, $CDCl_3$) δ 2.23 (dd, 1H, J_1 = 6.8 Hz, J_2 = 14.0 Hz), 2.30 (dd,

1H, J_1 = 6.8 Hz, J_2 = 14.0 Hz), 2.91-3.05 (br, 3H), 3.81 (s, 3H), 5.06 (d, 1H, J = 9.2 Hz), 5.07 (d, 1H, J = 17.2 Hz), 5.77 (m, 1H), 6.05 (d, 1H, J = 16.0 Hz), 6.07 (d, 1H, J = 15.6 Hz), 6.41 (d, 1H, J = 15.6 Hz), 7.21 (t, 1H, J = 6.8 Hz), 7.31 (m, 4H) ; ^{13}C NMR (400 MHz, $CDCl_3$) δ 39.9, 40.6, 44.7, 53.0, 117.7, 122.6, 126.3, 129.9, 130.1, 130.6, 135.3, 161.2, 192.4; IR (neat) cm^{-1} 3003, 2924, 2852, 1641, 1567, 1435, 1276, 920; HRMS (MALDI, m/z) calcd for $C_{16}H_{18}O_3Na$ $(M+Na)^+$: 281.1148, found 281.1150.

Preparation of 3n

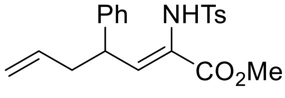

3n

3n: Using the same procedure as that used for **3a**. Methyl (2Z,3E)-4-phenyl-2-((phenylsulfonyl)-l4-azanylidene)but-3-enoate (73 mg, 0.21 mmol) and **2a** (42 μL, 0.26 mmol) in anhyd. CH_2Cl_2 (4 mL) under argon atmosphere with [$(Ph_3C)[BPh(^F)_4]$] (2 mg, 0.22 × 10^{-2} mmol) at 25 °C for 10 minutes afforded **3n** (61 mg, 75%). ^{1}H NMR (400 MHz, $CDCl_3$) δ 2.36 (s, 3H), 2.41-2.58 (m, 2H), 3.58 (s, 3H), 4.48 (dt, 1H, J_1 = 7.2 Hz, J_2 = 10.4 Hz), 4.97 (d, 1H, J = 10.0 Hz), 5.02 (d, 1H, J = 17.2 Hz), 5.84 (m, 1H), 6.59 (m, 1H), 6.66 (d, 1H, J = 10.8 Hz), 7.11 (d, 2H, J = 8.0 Hz), 7.18 (d, 2H, J = 8.0 Hz), 7.23 (t, 1H, J = 7.2 Hz), 7.31 (t, 2H, J = 7.2 Hz) 7.47 (d, 2H, J = 8.4 Hz); ^{13}C NMR (400 MHz, $CDCl_3$) δ 21.5, 40.9, 43.5, 52.3, 116.9, 123.3, 126.6, 127.5, 127.6, 128.6, 129.4, 135.4, 140.9, 142.9, 143.8, 148.0, 163.6; IR (neat) cm^{-1} 3265, 1724, 1640, 1598, 1493, 1435, 1304, 1166, 1136, 1090, 915; HRMS (MALDI, m/z) calcd for $C_{21}H_{23}NO_4SNa$ $(M+Na)^+$: 408.1240, found 408.1240.

Preparation of 3o

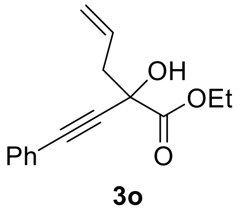

3o

3o: Using the same procedure as that used for **3a**. Ethyl 2-oxo-4-phenylbut-3-ynoate (40 mg, 0.21 mmol) and **2a** (42 μL, 0.26 mmol) in anhyd. CH_2Cl_2 (4 mL) under argon atmosphere with [$(Ph_3C)[BPh(^F)_4]$] (2 mg, 0.22 × 10^{-2} mmol) at 25 °C for 10 minutes afforded **3o** (45 mg, 93%) as a

yellow oil. ^{1}H NMR (400 MHz, $CDCl_3$) δ 1.35 (t, 3H, J = 7.2 Hz), 2.77 (dd, 1H, J_1 = 8.0 Hz, J_2 = 14.0 Hz), 2.86 (dd, 1H, J_1 = 6.8 Hz, J_2 = 14.0 Hz), 3.64 (s, 1H), 4.34(m, 2H), 5.21 (d, 1H, J = 10.0 Hz), 5.22 (d, 1H, J = 17.2 Hz), 5.90 (m, 1H), 7.30 (m, 3H), 7.43 (d, 1H, J = 2.0 Hz), 7.45 (d, 1H, J = 1.6 Hz),; ^{13}C NMR (400 MHz, $CDCl_3$) δ 14.1, 44.5, 63.0, 70.9, 84.9, 87.2, 119.8, 122.0, 128.2, 128.7, 131.3, 131.8, 171.9; IR (neat) cm^{-1} 3488, 2981, 1734, 1490, 1443, 1222, 1146, 1073, 1024, 921; HRMS (MALDI, m/z) calcd for $C_{15}H_{16}ONa$ $(M+Na)^+$: 267.0992, found 267.0994.

Preparation of 3p

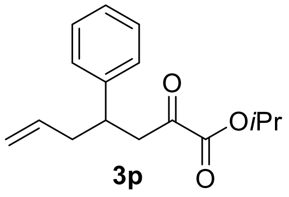

3p

3p: Using the same procedure as that used for **3a**. Isopropyl (E)-2-oxo-4-phenylbut-3-enoate (23 mg, 0.11 mmol) and **2a** (21 μL, 0.13 mmol) in anhyd. CH_2Cl_2 (2 mL) under argon atmosphere with [$(Ph_3C)[BPh(^F)_4]$] (1 mg, 0.11 × 10^{-2} mmol) at 25 °C for 10 minutes afforded **3o** (26 mg, 90%, H-S:D-A = 91:9) as a yellow oil. ^{1}H NMR (400 MHz, $CDCl_3$) δ 1.29 (t, 6H, J = 6.4 Hz), 2.37 (dd, 1H, J_1 = 7.6 Hz, J_2 = 13.6 Hz), 2.43 (dd, 1H, J_1 = 6.8 Hz, J_2 = 14.0 Hz), 3.16 (d, 2H, J = 7.2 Hz), 3.32 (dddd, 1H, J = 7.2 Hz), 4.98 (d, 1H, J = 8.4 Hz), 5.02 (d, 1H, J = 16.0 Hz), 5.07 (m, 1H), 5.65 (m, 1H), 7.19 (m, 3H), 7.27 (m, 2H); ^{13}C NMR (100 MHz, $CDCl_3$) δ 21.5, 40.4, 40.8, 44.9, 70.6, 117.2, 126.6, 127.5, 128.5, 135.9, 143.3, 160.5, 193.7; IR (neat) cm^{-1} 3029, 2923, 2852, 1723, 1494, 1377, 1278, 1184, 1104, 916; HRMS (MALDI, m/z) calcd for $C_{16}H_{20}O_3Na$ $(M+Na)^+$: 283.1305, found 283.1312.

Preparation of 3q

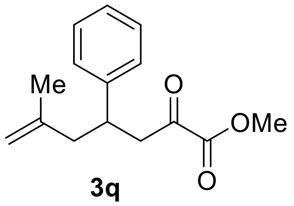

3q

3q: Using the same procedure as that used for **3a**. Methyl (*E*)-2-oxo-4-phenylbut-3-enoate (40 mg, 0.21 mmol) and **2b** (33 mg, 0.26 mmol) in anhyd. CH_2Cl_2 (4 mL) under argon atmosphere with

[$(Ph_3C)[BPh(^F)_4]$] (2 mg, 0.22 × 10^{-2} mmol) at 25 °C for 10 minutes afforded **3q** (49 mg, 95%) as a yellow oil. ^{1}H NMR (400 MHz, $CDCl_3$) δ 1.69 (s, 3H), 2.34 (d, 2H, J = 7.6 Hz), 3.11 (dd, 1H, J_1 = 8.0 Hz, J_2 = 17.6 Hz), 3.18 (dd, 1H, J_1 = 6.4 Hz, J_2 = 17.6 Hz), 3.46 (dddd, 1H, J = 7.6 Hz), 3.77 (s, 3H), 4.67 (s, 1H), 4.73 (s, 1H), 7.19 (m, 3H), 7.28 (t, 2H, J = 7.2 Hz); ^{13}C NMR (100 MHz, $CDCl_3$) δ 22.0, 38.6, 45.0, 45.2, 52.8, 113.3, 126.6, 127.3, 128.4, 143.1, 143.6, 161.2, 192.6; IR (neat) cm^{-1} 2927, 2854, 1731, 1643, 1441, 1287, 1250, 1217; HRMS (MALDI, m/z) calcd for $C_{28}H_{38}OSi_3Na$ $(M+Na)^+$: 269.1148, found 269.1143.

Preparation of 3r

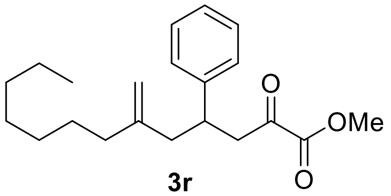

3r

3r: Using the same procedure as that used for **3a**. Methyl (*E*)-2-oxo-4-phenylbut-3-enoate (40 mg, 0.21 mmol) and **2c** (55 mg, 0.26 mmol) in anhyd. CH_2Cl_2 (4 mL) under argon atmosphere with [$(Ph_3C)[BPh(^F)_4]$] (2 mg, 0.22 × 10^{-2} mmol) at 25 °C for 10 minutes afforded **3q** (66 mg, 95%) as a yellow oil. ^{1}H NMR (400 MHz, $CDCl_3$) δ 0.89 (t, 3H, J = 7.2 Hz), 1.21-1.29 (m, 10H), 1.97 (m, 2H), 2.35 (m, 2H), 3.13 (dd, 1H, J_1 = 8.0 Hz, J_2 = 13.6 Hz), 3.20 (dd, 1H, J_1 = 6.8 Hz, J_2 = 14.0 Hz), 3.45 (dddd, 1H, J = 7.2 Hz), 3.79 (s, 3H), 4.70 (s, 1H), 4.75 (s, 1H), 7.20 (m, 3H), 7.29 (m, 2H); ^{13}C NMR (100 MHz, $CDCl_3$) δ 14.1, 22.6, 27.6, 29.1, 29.3, 31.8, 35.4, 38.8, 43.4, 45.1, 52.8, 112.1, 126.6, 127.4, 128.5, 143.7, 147.2, 161.2, 192.7; IR (neat) cm^{-1} 2925, 2855, 1730, 1643, 1494, 1453, 1286, 1235, 1069, 892; HRMS (MALDI, m/z) calcd for $C_{21}H_{30}O_3Na$ $(M+Na)^+$: 353.2087, found 353.2089.

Preparation of 3s

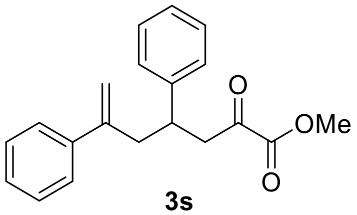

3s

3s: Using the same procedure as that used for **3a**. Methyl (*E*)-2-oxo-4-phenylbut-3-enoate (40 mg, 0.21 mmol) and **2d** (48 mg, 0.26 mmol) in anhyd. CH_2Cl_2 (4 mL) under argon atmosphere with

[(Ph$_3$C)[BPh(^{F})$_4$]] (2 mg, 0.22 × 10^{-2} mmol) at 25 °C for 10 minutes afforded **3s** (55 mg, 85%) as a yellow oil. ^{1}H NMR (400 MHz, CDCl$_3$) δ 2.82 (dd, 1H, J_1 = 7.6 Hz, J_2 = 14.0 Hz), 2.87 (dd, 1H, J_1 = 7.6 Hz, J_2 = 13.6 Hz), 3.19 (d, 2H, J = 7.2 Hz), 3.33 (dddd, 1H, J = 7.2 Hz), 3.76 (s, 3H), 4.93 (s, 1H), 5.22 (s, 1H), 7.11 (d, 2H, J = 7.6 Hz), 7.19 (m, 2H), 7.24-7.35 (br, 6H); ^{13}C NMR (100 MHz, CDCl$_3$) δ 38.9, 42.6, 44.9, 52.9, 115.2, 126.3, 126.7, 127.5, 128.4, 128.5, 140.2, 143.2, 145.9, 161.2, 192.7; IR (neat) cm^{-1} 2927, 1728, 1494, 1448, 1264, 1069, 903; HRMS (MALDI, m/z) calcd for $C_{20}H_{20}O_3Na$ (M+Na)$^+$: 331.1305, found 331.1304.

Preparation of 3t

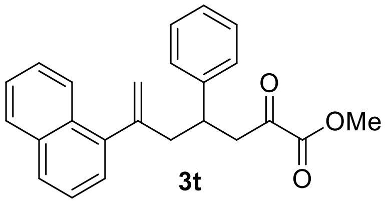

3t

3t: Using the same procedure as that used for **3a**. Methyl (*E*)-2-oxo-4-phenylbut-3-enoate (40 mg, 0.21 mmol) and **2e** (63 mg, 0.26 mmol) in anhyd. CH_2Cl_2 (4 mL) under argon atmosphere with [(Ph$_3$C)[BPh(^{F})$_4$]] (2 mg, 0.22 × 10^{-2} mmol) at 25 °C for 10 minutes afforded **3t** (72 mg, 96%) as a yellow oil. ^{1}H NMR (400 MHz, CDCl$_3$) δ 2.93 (m, 2H), 3.22 (d, 1H, J = 6.0 Hz), 3.24 (d, 1H, J = 7.2 Hz), 3.32 (m, 1H), 5.14 (s, 1H), 5.38 (s, 1H), 7.13 (d, 2H, J = 7.2 Hz), 7.18 (t, 2H, J = 7.2 Hz), 7.26 (m, 2H), 7.46 (m, 3H), 7.89 (d, 1H, J = 8.4 Hz), 7.86 (dd, 2H, J_1 = 2.8 Hz, J_2 = 8.0 Hz); ^{13}C NMR (100 MHz, CDCl$_3$) δ 39.0, 44.6, 45.6, 52.8, 118.3, 125.1, 125.2, 125.6, 125.7, 126.7, 127.5, 127.7, 128.3, 128.4, 131.0, 133.7, 140.1, 142.9, 145.7, 161.1, 192.7; IR (neat) cm^{-1} 3030, 2951, 1727, 1494, 1453, 1436, 1394, 1271, 1250, 1069, 909, 804; HRMS (MALDI, m/z) calcd for $C_{24}H_{22}O_3Na$ (M+Na)$^+$: 381.1461, found 381.1460.

Preparation of 3u

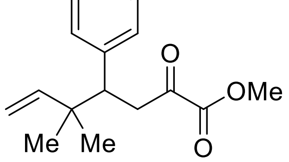

3u

3u: Using the same procedure as that used for **3a**. Methyl (*E*)-2-oxo-4-phenylbut-3-enoate (40 mg,

0.21 mmol) and **2f** (37 mg, 0.26 mmol) in anhyd. CH_2Cl_2 (4 mL) under argon atmosphere with [(Ph_3C)[$BPh(^F)_4$]] (2 mg, 0.22 × 10^{-2} mmol) at 25 °C for 10 minutes afforded **3u** (51 mg, 93%) as a yellow oil. ^{1}H NMR (400 MHz, $CDCl_3$) δ 0.94 (s, 3H), 0.96 (s, 3H), 3.12-3.20 (m, 2H), 3.36 (m, 1H), 3.75 (s, 3H), 4.99 (d, 1H, J = 17.6 Hz), 5.05 (d, 1H, J = 10.0 Hz), 5.82 (dd, 1H, J_1 = 6.8 Hz, J_2 = 17.6 Hz), 7.17 (t, 3H, J = 6.8 Hz), 7.25 (m, 2H); ^{13}C NMR (100 MHz, $CDCl_3$) δ 22.4, 26.9, 40.0, 40.8, 49.6, 52.8, 112.7, 126.7, 128.4, 129.5, 140.2, 146.4, 161.4, 193.3; IR (neat) cm^{-1} 2961, 2874, 1731, 1453, 1269, 1069, 917, 843; HRMS (MALDI, m/z) calcd for $C_{16}H_{20}O_3Na$ $(M+Na)^+$: 283.1305, found 283.1306.

<u>Preparation of 3v</u>

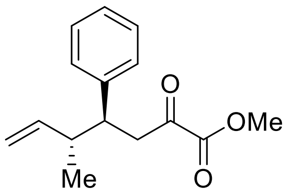

3v

3v: Using the same procedure as that used for **3a**. Methyl (*E*)-2-oxo-4-phenylbut-3-enoate (40 mg, 0.21 mmol) and **2g-Z** (42 μL, 0.26 mmol) in anhyd. CH_2Cl_2 (4 mL) under argon atmosphere with [(Ph_3C)[$BPh(^F)_4$]] (2 mg, 0.22 × 10^{-2} mmol) at 25 °C for 10 minutes afforded **3v** (53 mg, 98%) as a colorless oil. *anti:syn* = 3:2.

3v: Using the same procedure as that used for **3a**. Methyl (*E*)-2-oxo-4-phenylbut-3-enoate (40 mg, 0.21 mmol) and **2g-E** (42 μL, 0.26 mmol) in anhyd. CH_2Cl_2 (4 mL) under argon atmosphere with [(Ph_3C)[$BPh(^F)_4$]] (2 mg, 0.22 × 10^{-2} mmol) at 25 °C for 10 minutes afforded **3v** (49 mg, 91%) as a colorless oil. *anti:syn* = 3:1. ^{1}H NMR (400 MHz, $CDCl_3$) δ 0.73 (d, 3H, J = 6.8 Hz, *syn*), 0.90 (d, 3H, J = 6.8 Hz, *anti*), 2.33-2.24 (m, 1H, *syn*), 2.43-2.35 (m, 1H, *anti*), 3.25-2.94 (m, 1H), 3.25-2.94 (m, 2H), 3.68 (s, 3H, *syn*), 3.71 (s, 3H, *anti*), 4.87 (d, 1H, J = 17.6 Hz,), 4.90 (d, 1H, J = 10.8 Hz), 5.53 (ddd, 1H, J_1 = 17.2 Hz, J_2 = 10.8 Hz, J_3 = 8.4 Hz), 7.22-7.05 (m, 5H); ^{13}C NMR (100 MHz, $CDCl_3$) δ17.4, 42.2, 42.4, 45.2, 52.8, 115.1, 126.6, 128.1, 128.6, 140.8, 142.8, 161.4, 193.2; IR (neat) cm^{-1} 3029, 2960, 2923, 1728, 1453, 1262, 1065, 916, 801, 761; HRMS (MALDI, m/z) calcd for $C_{15}H_{18}O_3$ $(M+Na)^+$: 269.1148, found 269.1149.

2.2. Preparations and Spectral Data of 5

<u>Preparation of 5a</u>

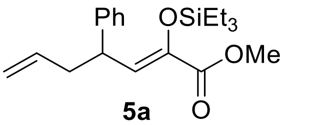

5a

5a: To a solution of β,γ-unsaturated α-ketoester **1a** (40 mg, 0.22 mmol) and allyltriethylsilane **2h** (40 mg, 0.26 mmol) and in anhyd. CH_2Cl_2 (4 mL) under argon atmosphere was added [(Ph_3C)[$BPh(^F)_4$]] (2 mg, 0.22 × 10^{-2} mmol) at 25 °C. After stirring for 10 minutes, the reaction was quenched with NEt_3 (134 mg, 1.32 mmol). The mixture was directly concentrated under reduced pressure. Purification of the crude residue via silica gel flash column chromatography (gradient eluent: 0-2.0% of EtOAc /petroleum ether) afforded **5a** (74 mg, 97%) as a colorless oil. ^{1}H NMR (600 MHz, $CDCl_3$) δ 0.79 (q, 6H, *J* = 7.8 Hz), 1.05 (t, 9H, *J* = 7.8 Hz), 2.56 (m, 2H), 3.82 (s, 3H), 3.99 (m, 1H), 5.05 (d, 1H, *J* = 10.2 Hz), 5.11 (d, 1H, *J* = 16.8 Hz), 5.78 (m, 1H), 7.30 (m, 3H), 7.38 (m, 2H); ^{13}C NMR (100 MHz, $CDCl_3$) δ 5.5, 6.8, 40.6, 41.8, 51.9, 116.4, 124.6, 126.4, 127.5, 128.5, 135.0, 140.1, 143.3, 165.3; IR (neat) cm^{-1} 2955, 2878, 1727, 1642, 1439, 1372, 1266, 1232, 1143, 1010, 913; HRMS (MALDI, m/z) calcd for $C_{20}H_{30}O_3SiNa$ $(M+Na)^+$: 369.1856, found 369.1856.

Preparation of 5b

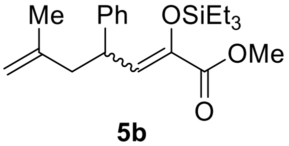

5b

5b: Using the same procedure as that used for **5a**. Methyl (*E*)-2-oxo-4-phenylbut-3-enoate (40 mg, 0.21 mmol) and **2i** (44 mg, 0.26 mmol) in anhyd. CH_2Cl_2 (4 mL) under argon atmosphere with [(Ph_3C)[$BPh(^F)_4$]] (2 mg, 0.22 × 10^{-2} mmol) at 25 °C for 10 minutes and quenching with NEt_3 (134 mg, 1.32 mmol) afforded **5b** (74 mg, 93%, *Z*:*E* = 72:28) as a yellow oil. ^{1}H NMR (600 MHz, $CDCl_3$), *Z*-isomer: δ 0.70 (q, 6H, *J* = 7.8 Hz), 0.96 (t, 9H, *J* = 7.8 Hz), 1.70 (s, 3H), 2.41 (m, 2H), 3.72 (s, 3H), 4.06 (m, 1H), 4.67 (m, 2H), 6.11 (d, 1H, *J* = 10.2 Hz), 7.17 (t, 1H, *J* = 7.2 Hz), 7.22 (d, 2H, *J* = 7.2 Hz), 7.28 (t, 2H, *J* = 7.2 Hz); δ 5.6, 6.8, 22.4, 40.2, 44.7, 51.9, 112.4, 125.0, 126.3, 127.4, 128.4, 139.8, 143.0, 143.6, 165.3; ***E***-isomer: δ 0.64 (q, 6H, *J* = 7.8 Hz), 0.94 (t, 9H, *J* = 7.8 Hz), 1.71 (s, 3H), 2.41 (m, 2H), 3.75 (s, 3H), 4.06 (m, 1H), 4.67 (m, 2H), 5.58 (d, 1H, *J* = 10.8 Hz), 7.17 (t, 1H, *J* = 7.2 Hz), 7.22 (d, 2H, *J* = 7.2 Hz), 7.28 (t, 2H, *J* = 7.2 Hz); ^{13}C NMR (100 MHz, $CDCl_3$) δ 4.7, 6.5, 22.1, 40.2, 45.7, 51.5, 112.5, 125.0, 126.1, 127.3, 128.4, 139.5, 143.3, 144.5, 165.0; IR (neat) cm^{-1} 3064,

2955, 2879, 1726, 1641, 1489, 1439, 1372, 1230, 1139, 1008, 890; HRMS (MALDI, m/z) calcd for $C_{21}H_{32}O_3SiNa$ (M+Na)$^+$: 383.2013, found 383.2008.

Preparation of 5c

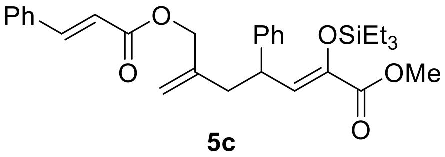

5c: Using the same procedure as that used for **5a**. Methyl (*E*)-2-oxo-4-phenylbut-3-enoate (20 mg, 0.21 mmol) and **2j** (41 mg, 0.13 mmol) in anhyd. CH_2Cl_2 (2 mL) under argon atmosphere with [(Ph_3C)[BPh(^{F})$_4$]] (1 mg, 0.11× 10^{-2} mmol) at 25 °C for 10 minutes and quenching with NEt_3 (67 mg, 0.66 mmol) afforded **5c** (35 mg, 62%) as a yellow oil. ^{1}H NMR (400 MHz, $CDCl_3$) δ 0.70 (q, 6H, *J* = 8.0 Hz), 0.95 (t, 9H, *J* = 8.0 Hz), 2.50 (dd, 1H, J_1 = 7.6 Hz, J_2 = 14.8 Hz), 2.59 (dd, 1H, J_1 = 7.2 Hz, J_2 = 14.8 Hz), 3.73 (s, 3H), 4.10 (q, 1H, *J* = 8.0 Hz), 4.57 (d, 1H, *J* = 13.2 Hz), 4.63 (d, 1H, *J* = 13.2 Hz), 4.97 (s, 1H), 5.11 (s, 1H), 8.13 (d, 1H, *J* = 10.0 Hz), 8.46 (d, 1H, *J* = 16.0 Hz), 7.18-7.31 (br, 5H), 7.39 (t, 3H, *J* = 2.8 Hz), 7.53 (m, 2H), 7.70 (d, 1H, *J* = 16.0 Hz); ^{13}C NMR (100 MHz, $CDCl_3$) δ 5.6, 6.8, 40.0, 40.2, 52.0, 66.9, 114.9, 117.9, 124.3, 126.5, 127.4, 128.1, 128.6, 128.9, 130.3, 134.3, 140.1, 141.3, 143.2, 145.0, 165.2, 166.5; IR (neat) cm^{-1} 2955, 2878, 1723, 1640, 1450, 1008; HRMS (MALDI, m/z) calcd for $C_{30}H_{38}O_5SiNa$ (M+Na)$^+$: 529.2381, found 529.2382.

Preparation of 5d

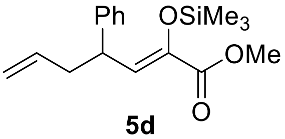

5d: Using the same procedure as that used for **5a**. Methyl (*E*)-2-oxo-4-phenylbut-3-enoate (40 mg, 0.21 mmol) and **2a** (42 μL, 0.26 mmol) in anhyd. CH_2Cl_2 (4 mL) under argon atmosphere with [(Ph_3C)[BPh(^{F})$_4$]] (2 mg, 0.22× 10^{-2} mmol) at 25 °C for 10 minutes and quenching with NEt_3 (67 mg, 0.66 mmol) afforded **5d**. The crude **5d** was directly used for ^{1}H NMR experiment without purification. ^{1}H NMR (600 MHz, $CDCl_3$) δ 0.28 (s, 9H), 2.53 (ddd, 1H, J_1 = J_2 = 7.2 Hz, J_3 = 13.8 Hz), 2.60 (ddd, 1H, J_1 = J_2 = 7.2 Hz, J_3 = 13.8 Hz), 3.83 (s, 3H), 3.94 (m, 1H), 5.07 (d, 1H, *J* = 10.2 Hz), 5.13 (d, 1H, *J* = 16.8 Hz), 6.30 (d, 1H, *J* = 9.6 Hz), 7.30 (m, 3H), 7.39 (m, 2H).

Preparation of 5e

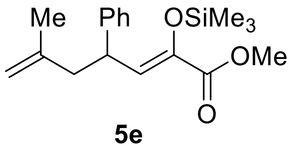

5e

5e: Using the same procedure as that used for **5a**. Methyl (*E*)-2-oxo-4-phenylbut-3-enoate (40 mg, 0.21 mmol) and **2b** (33 mg, 0.26 mmol) in anhyd. CH_2Cl_2 (4 mL) under argon atmosphere with $[(Ph_3C)[BPh(^F)_4]]$ (2 mg, 0.22× 10^{-2} mmol) at 25 °C for 10 minutes and quenching with NEt_3 (134 mg, 1.32 mmol) afforded **5e**. The crude **5e** was directly used for ^1H NMR experiment without purification. ^1H NMR (600 MHz, $CDCl_3$) δ 0.28 (s, 9H), 1.80 (s, 3H), 2.48 (dd, 1H, J_1 = 8.4 Hz, J_2 = 14.4 Hz), 2.54 (dd, 1H, J_1 = 7.2 Hz, J_2 = 13.8 Hz), 3.82 (s, 3H), 4.08 (m, 1H), 4.74 (s, 1H), 4.80 (s, 1H), 6.26 (d, 1H, J = 9.6 Hz), 7.29 (m, 3H), 7.38 (m, 2H).

2.3. Control Experiments

Reaction of 2a and 2i with 1a

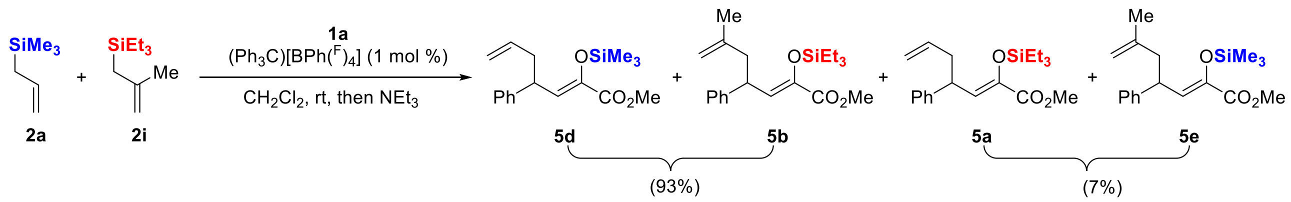

Scheme S1. Reaction of **2a** and **2i** with **1a**.

Using the same procedure as that used for **5a**. Methyl (*E*)-2-oxo-4-phenylbut-3-enoate (40 mg, 0.21 mmol), **2a** (21 μL, 0.13 mmol) and **2i** (22 mg, 0.13 mmol) in anhyd. CH_2Cl_2 (4 mL) under argon atmosphere with $[(Ph_3C)[BPh(^F)_4]]$ (2 mg, 0.22× 10^{-2} mmol) at 25 °C for 10 minutes and quenching with NEt_3 (67 mg, 0.66 mmol) afforded a mixture of **5d**, **5b**, **5a** and **5e**. The crude product was directly used for ^1H NMR experiment without purification.

Reaction of 2h and 2b with 1a

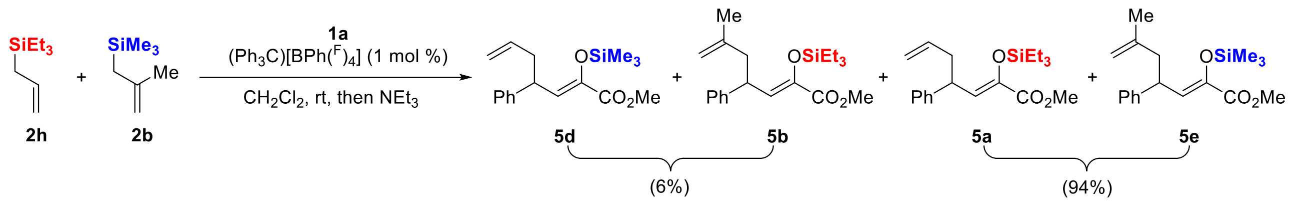

Scheme S2. Reaction of **2h** and **2b** with **1a**.

Using the same procedure as that used for **5a**. Methyl (*E*)-2-oxo-4-phenylbut-3-enoate (40 mg, 0.21 mmol), **2h** (20 mg, 0.13 mmol) and **2b** (17 mg, 0.13 mmol) in anhyd. CH_2Cl_2 (4 mL) under argon atmosphere with $[(Ph_3C)[BPh(^F)_4]]$ (2 mg, 0.22×10^{-2} mmol) at 25 °C for 10 minutes and quenching with NEt_3 (67 mg, 0.66 mmol) afforded a mixture of **5d**, **5b**, **5a** and **5e**. The crude product was directly used for ^{1}H NMR experiment without purification.

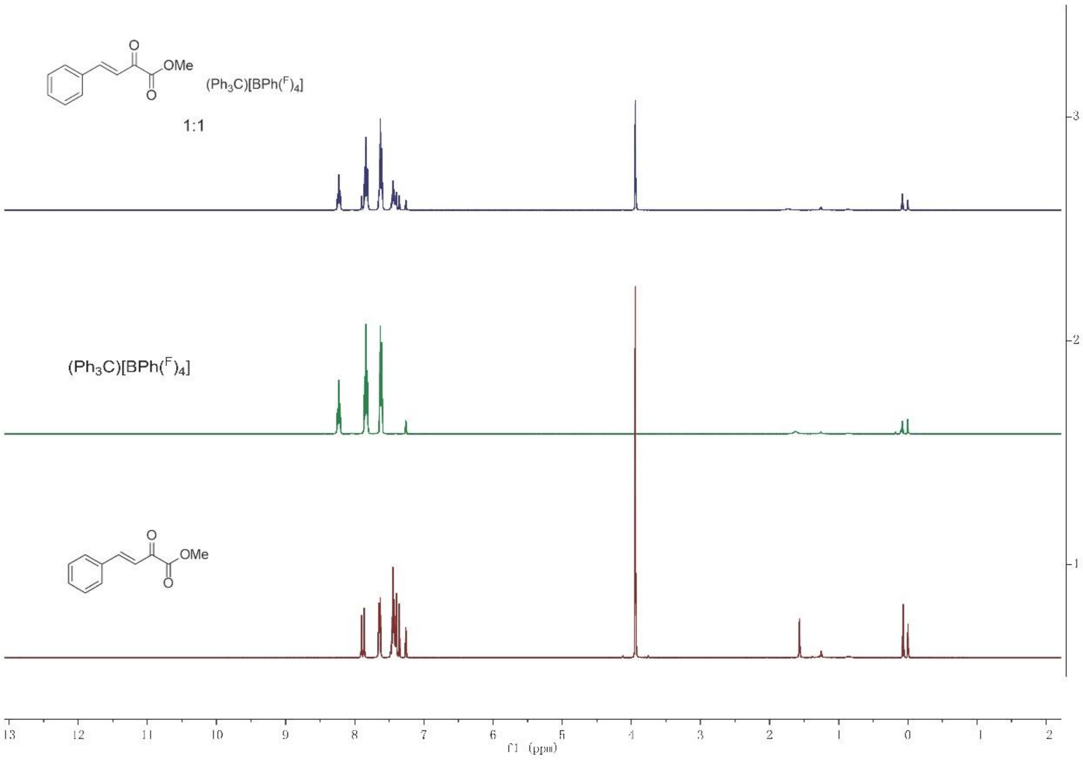
OMe
(Ph3C)[BPh(F)4]
1:1
(Ph3C)[BPh(F)4]
OMe
f1 (ppm)

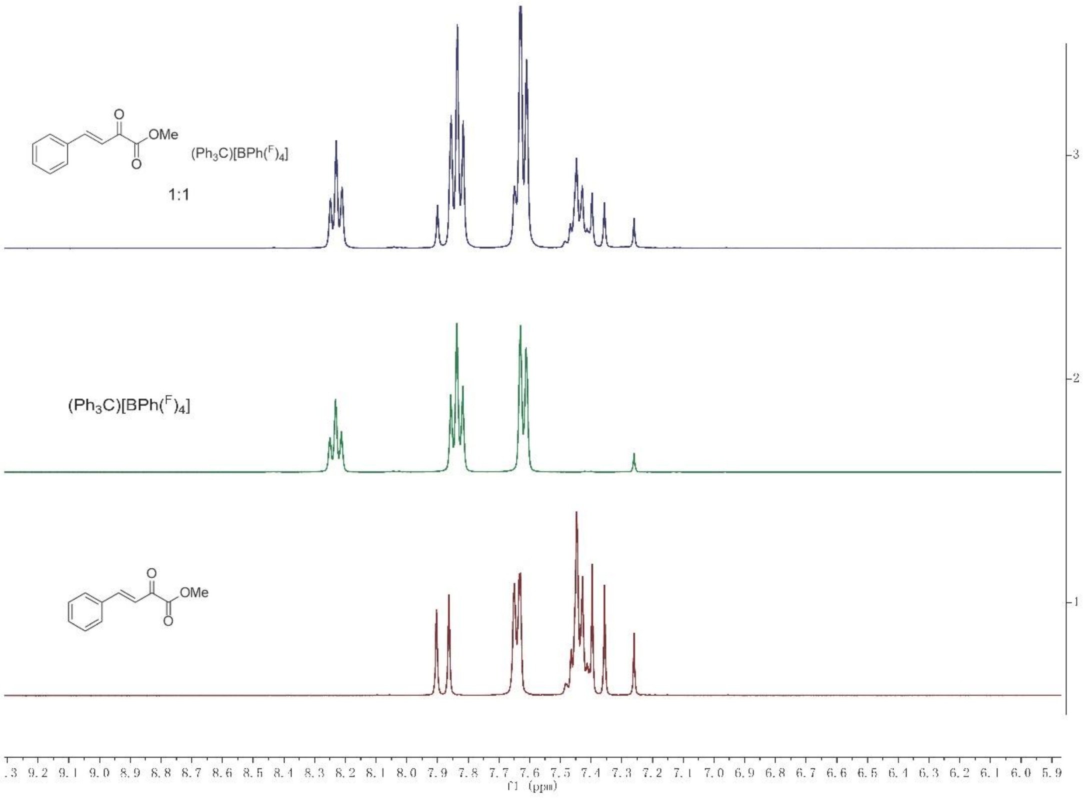
OMe
(Ph3C)[BPh(F)4]
1:1
(Ph3C)[BPh(F)4]
OMe
f1 (ppm)

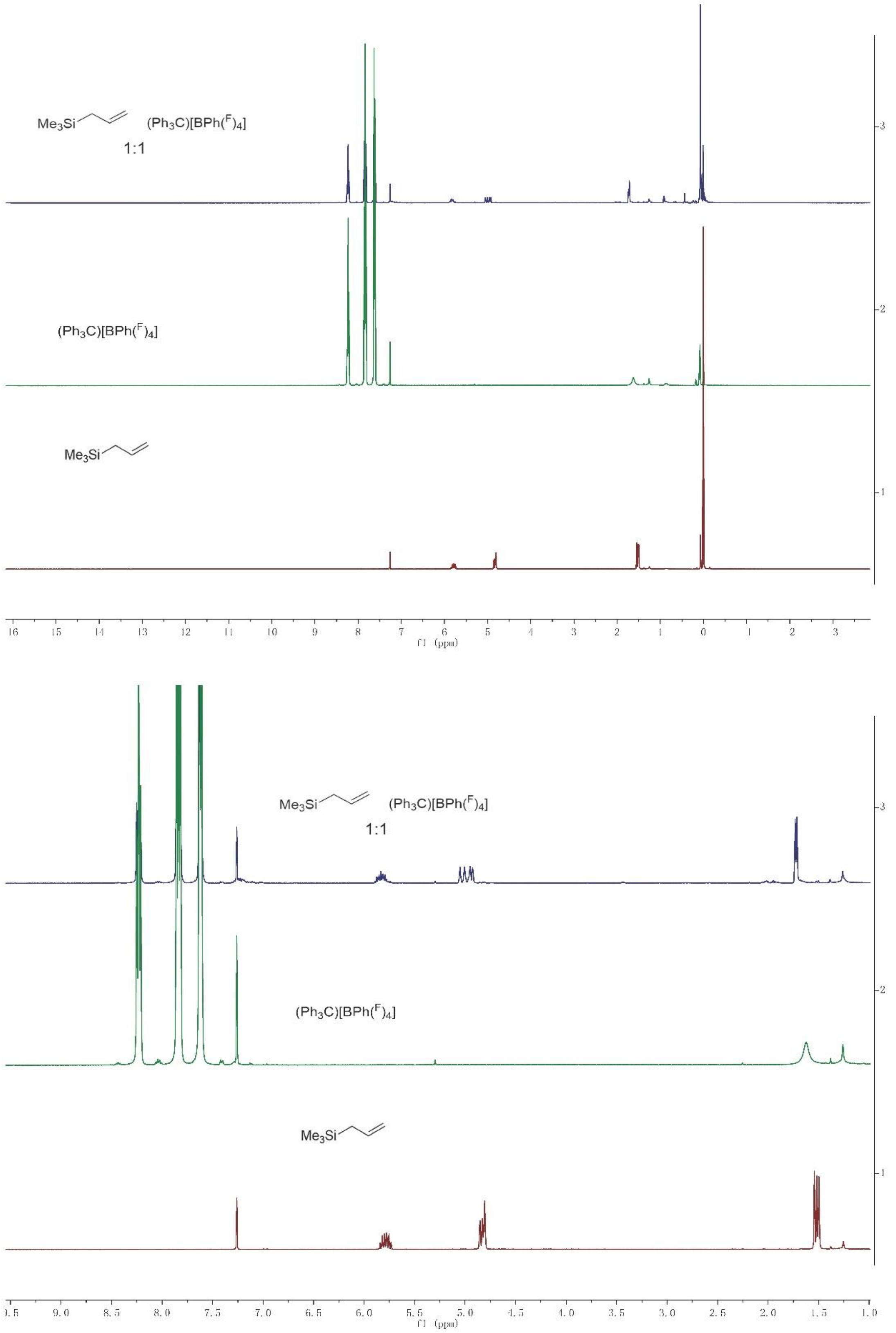

Scheme S3. The ^{1}H NMR titration experiment of [(Ph_3C)[BPh(^{F})$_4$]] with β,γ-unsaturated α-ketoester **1a** and allylsilane **2a**.

Gan-14-43-p H1 CDCl3
2015-11-11 400MHZ

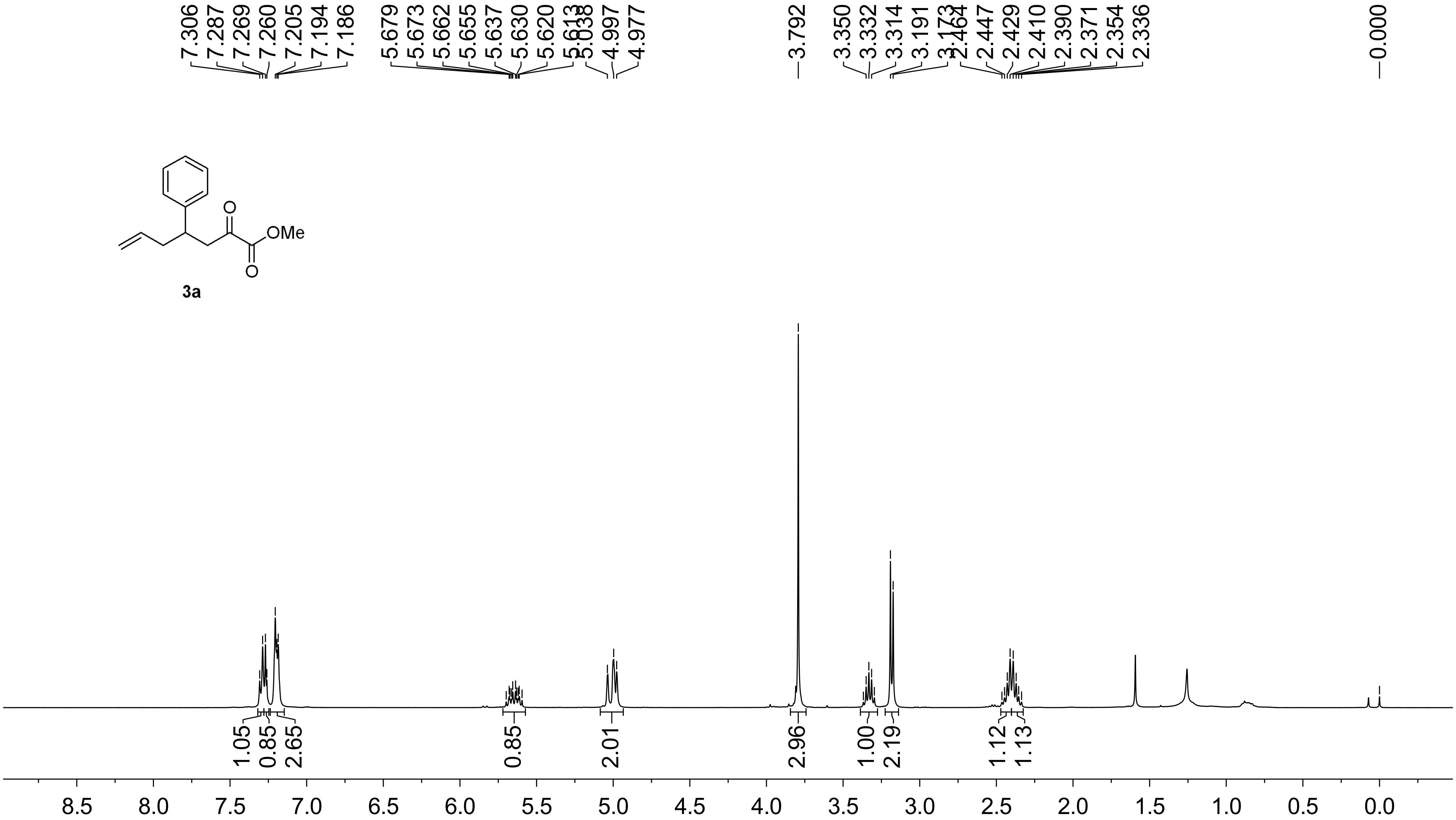

Gan-14-43-p C13 CDCl3 2015-11-12 100MHZ

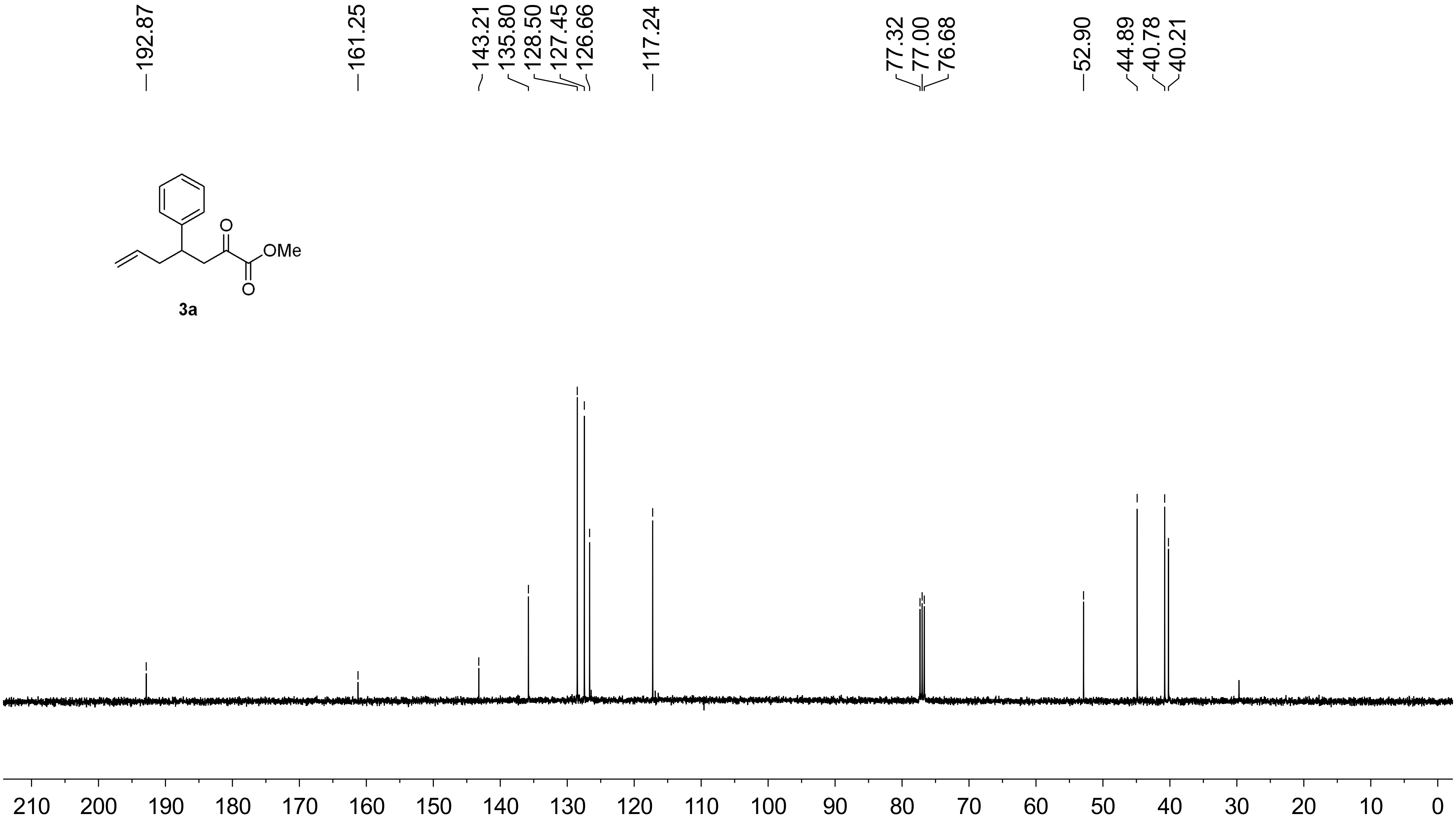

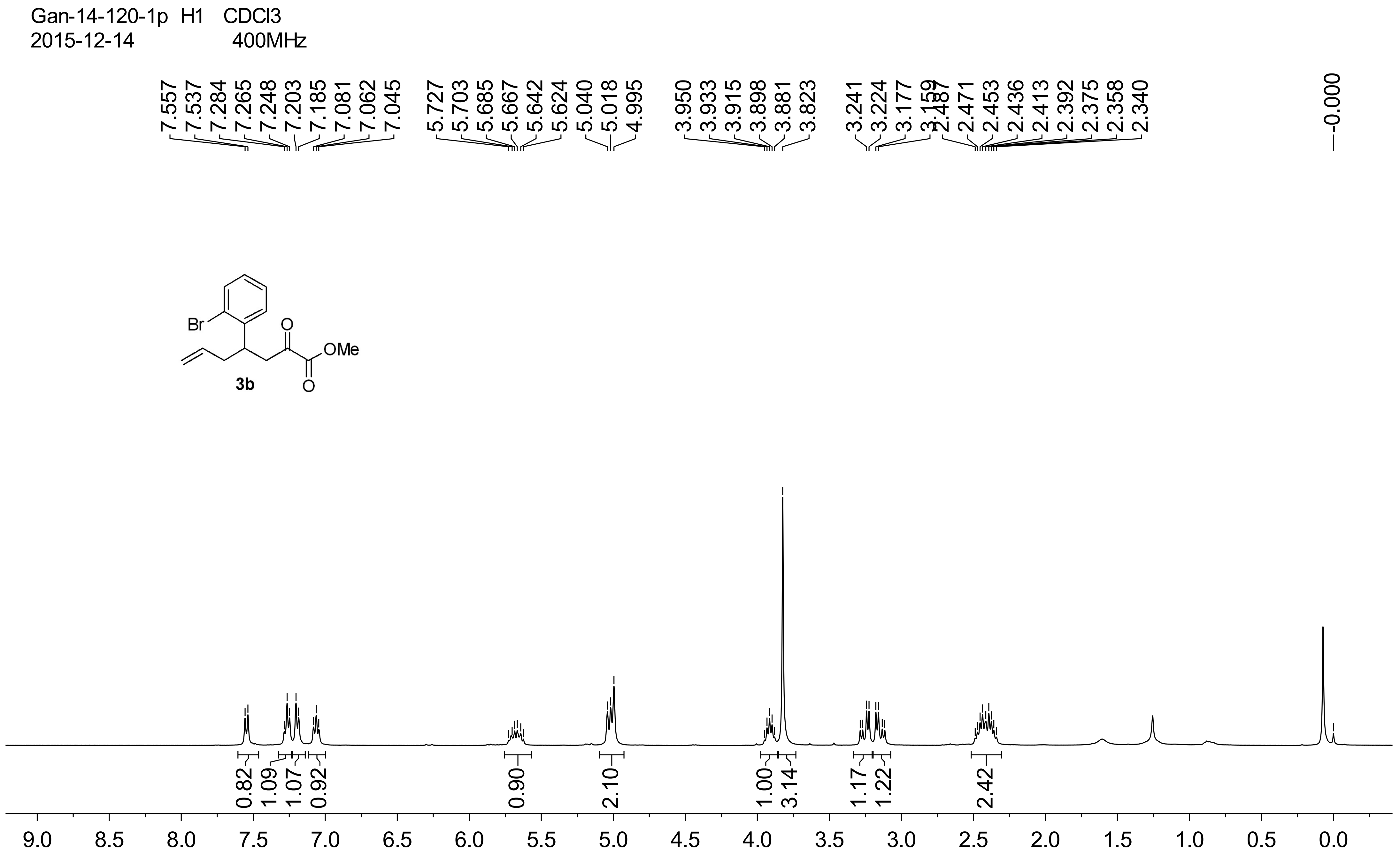

Gan-14-120-1p H1 CDCl3
2015-12-14 400MHz
7.557 7.537 7.284 7.265 7.248 7.203 7.185 7.081 7.062 7.045
5.727 5.703 5.685 5.667 5.642 5.624 5.040 5.018 4.995
3.950 3.933 3.915 3.898 3.881 3.823
3.241 3.224 3.177 3.159 2.487 2.471 2.453 2.436 2.413 2.392 2.375 2.358 2.340
-0.000
Br
O
OMe
O
3b
0.82 1.09 1.07 0.92 0.90 2.10 1.00 3.14 1.17 1.22 2.42
9.0 8.5 8.0 7.5 7.0 6.5 6.0 5.5 5.0 4.5 4.0 3.5 3.0 2.5 2.0 1.5 1.0 0.5 0.0

Gan-14-120-1p C13 CDCl3
2015-12-14 100MHz

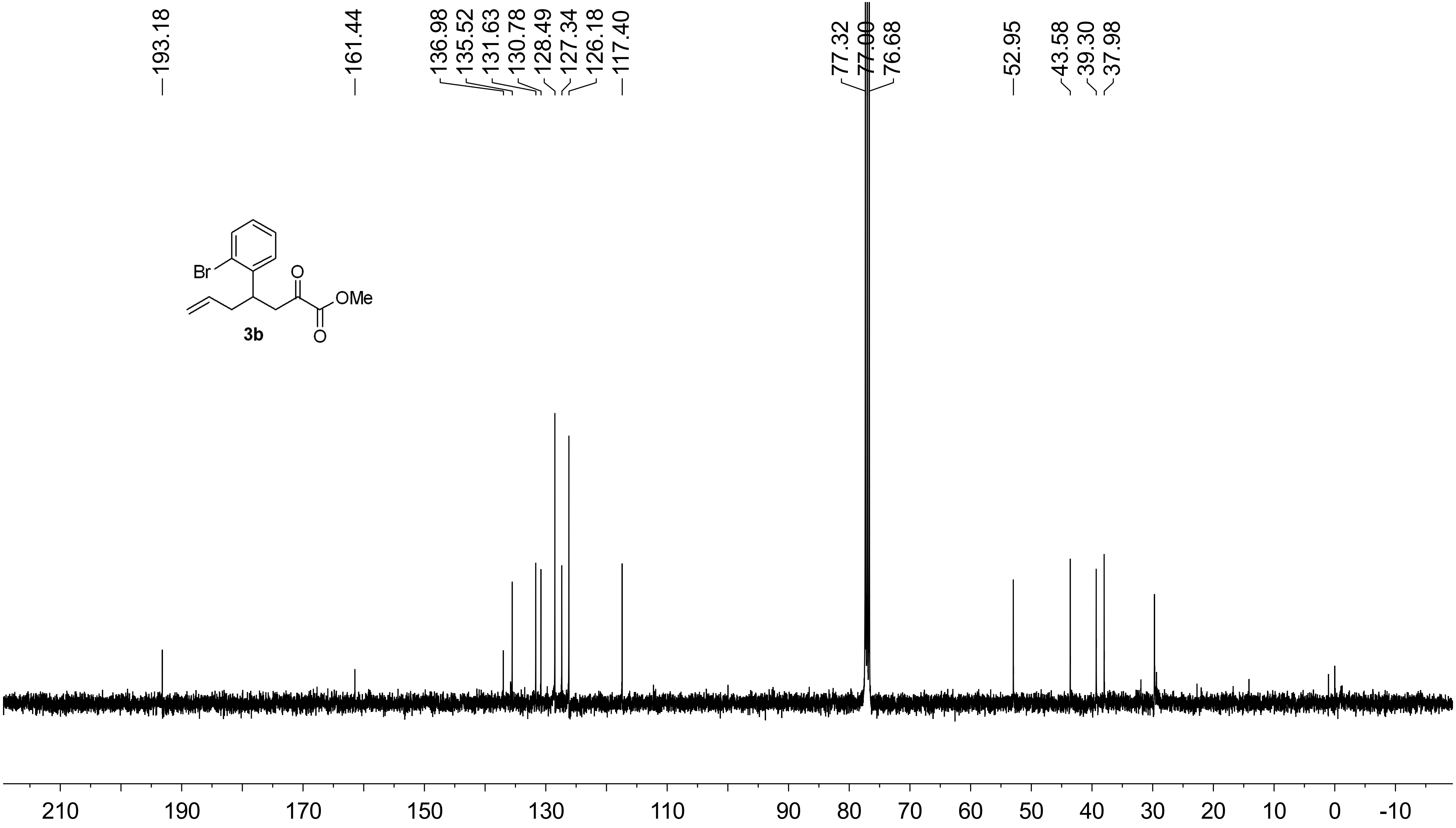

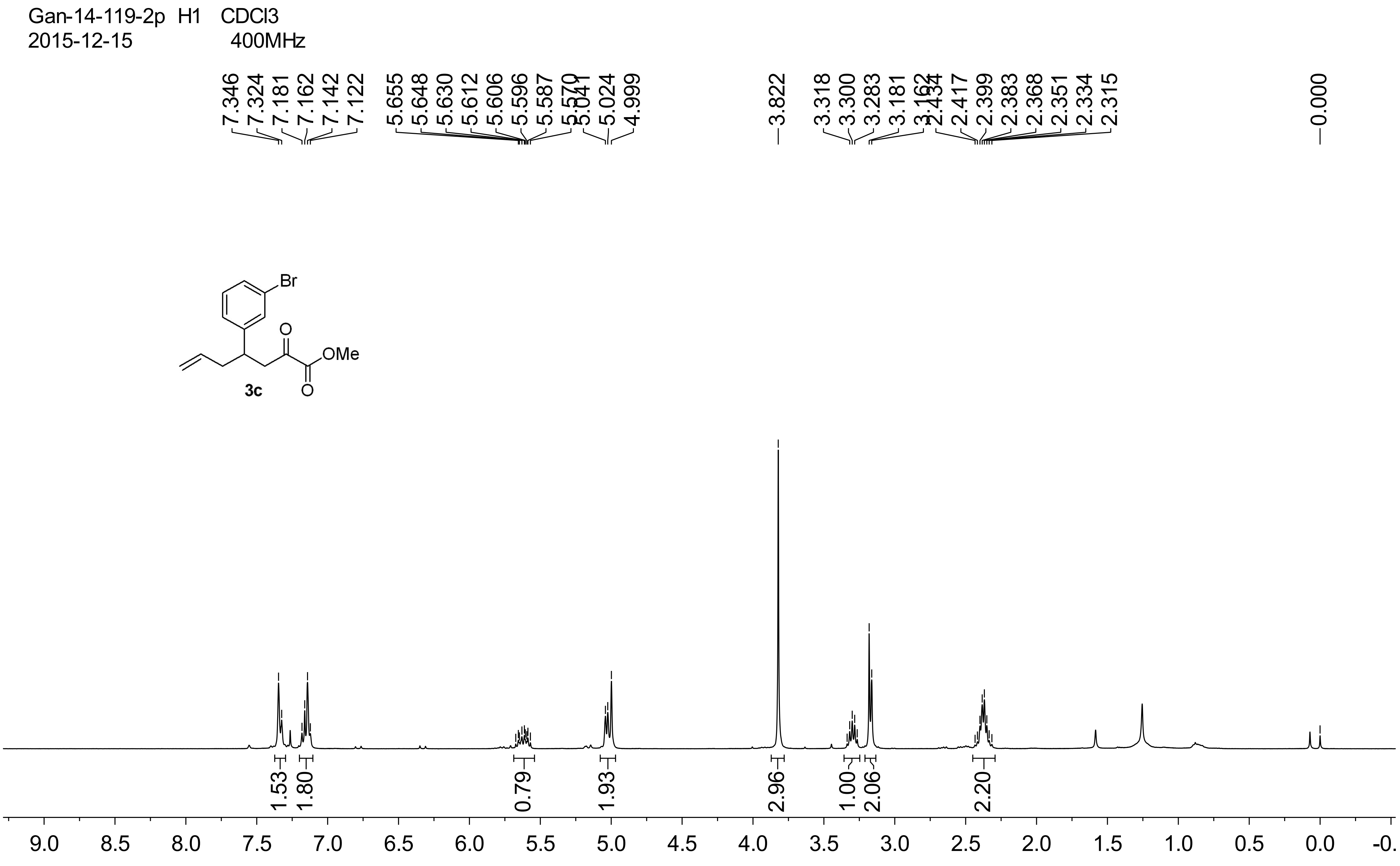
Gan-14-119-2p H1 CDCl3
2015-12-15 400MHz
7.346 7.324 7.181 7.162 7.142 7.122
5.655 5.648 5.630 5.612 5.606 5.596 5.587 5.570
5.041 5.024 4.999
3.822
3.318 3.300 3.283 3.181 3.162
2.434 2.417 2.399 2.383 2.368 2.351 2.334 2.315
0.000
Br
O
OMe
O
3c
1.53 1.80 0.79 1.93 2.96 1.00 2.06 2.20
9.0 8.5 8.0 7.5 7.0 6.5 6.0 5.5 5.0 4.5 4.0 3.5 3.0 2.5 2.0 1.5 1.0 0.5 0.0 -0.

Gan-14-119-2p C13 CDCl3
2015-12-15 100MHz

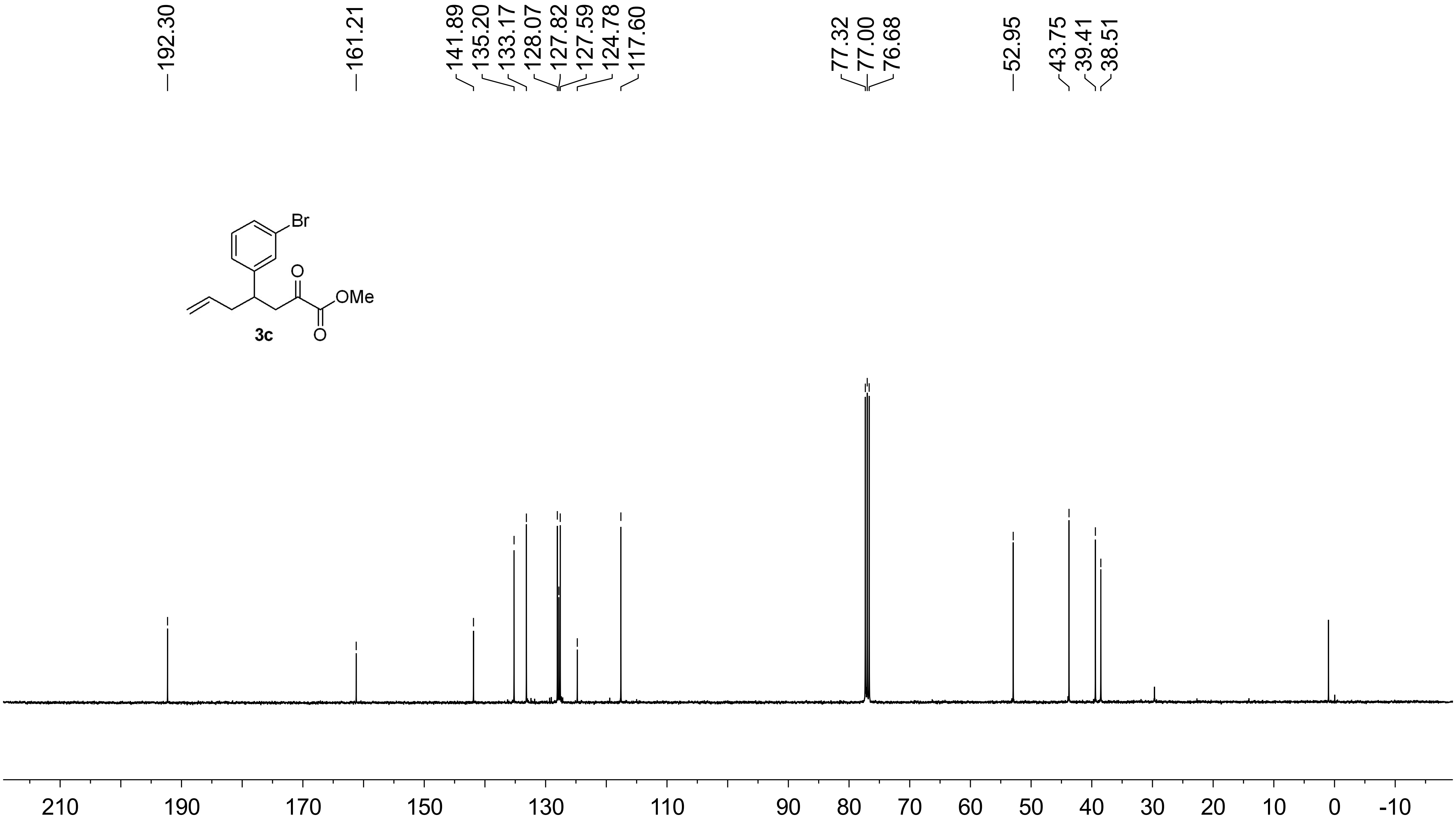

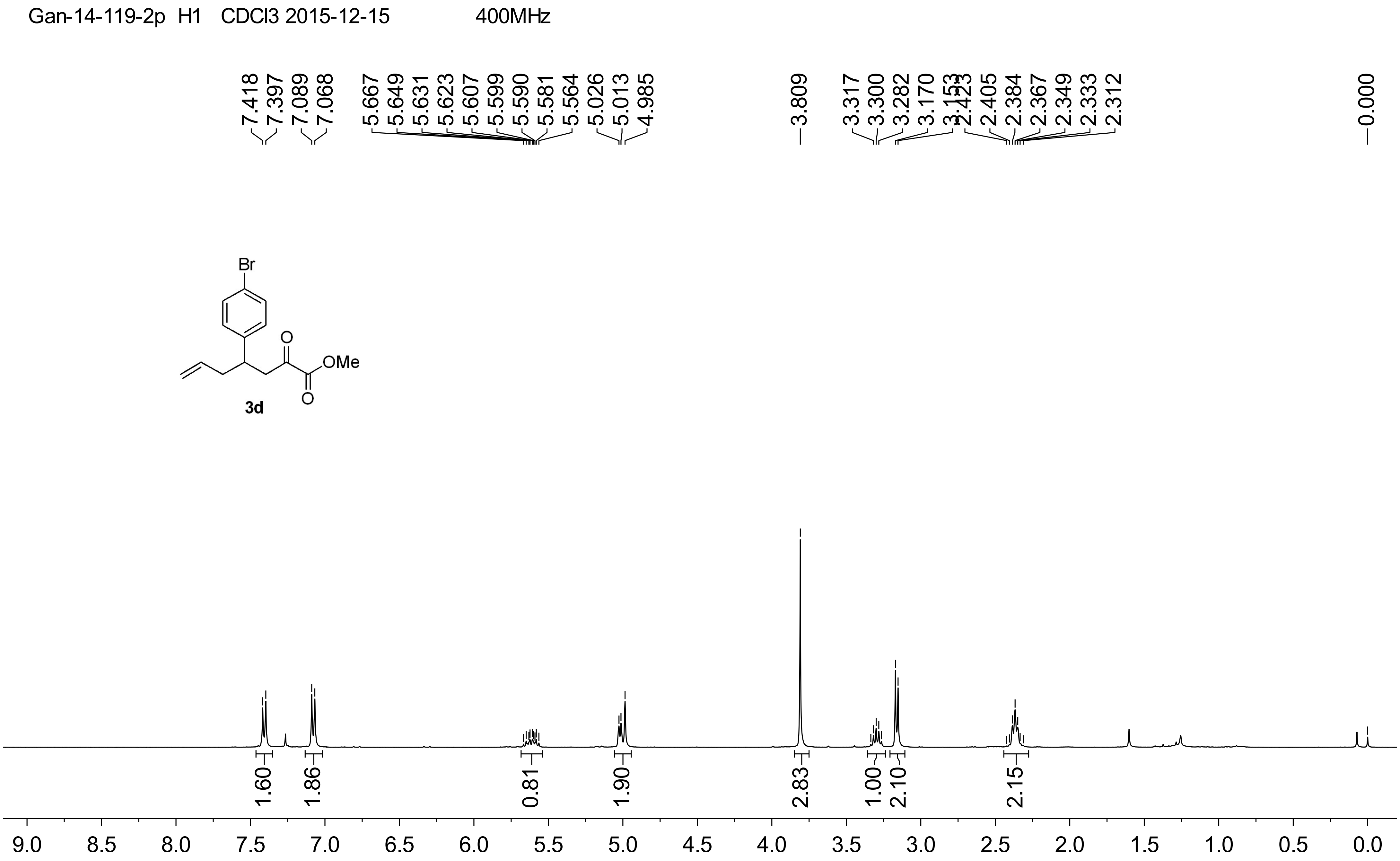
Gan-14-119-2p H1 CDCl3 2015-12-15 400MHz
7.418
7.397
7.089
7.068
5.667
5.649
5.631
5.623
5.607
5.599
5.590
5.581
5.564
5.026
5.013
4.985
3.809
3.317
3.300
3.282
3.170
3.153
2.423
2.405
2.384
2.367
2.349
2.333
2.312
0.000
Br
O
OMe
O
3d
1.60
1.86
0.81
1.90
2.83
1.00
2.10
2.15
9.0
8.5
8.0
7.5
7.0
6.5
6.0
5.5
5.0
4.5
4.0
3.5
3.0
2.5
2.0
1.5
1.0
0.5
0.0

Gan-14-119-2p C13 CDCl3 2015-12-23 100MHz

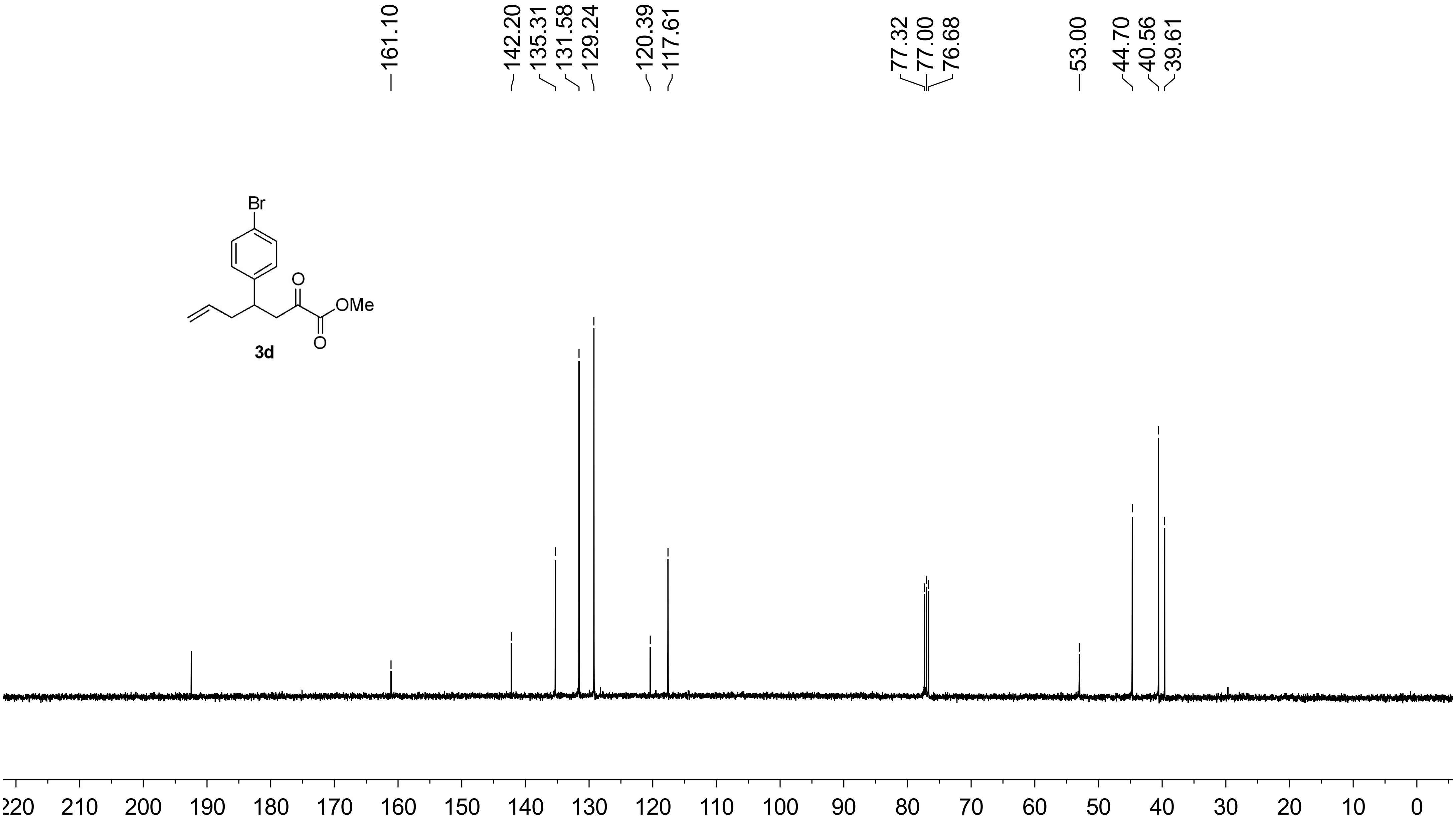

Gan-14-120-2P H1 CDCl3
2015-12-14 400MHz

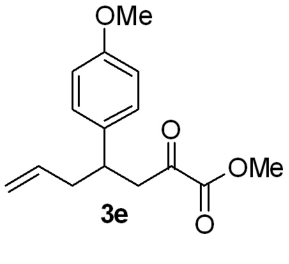

3e

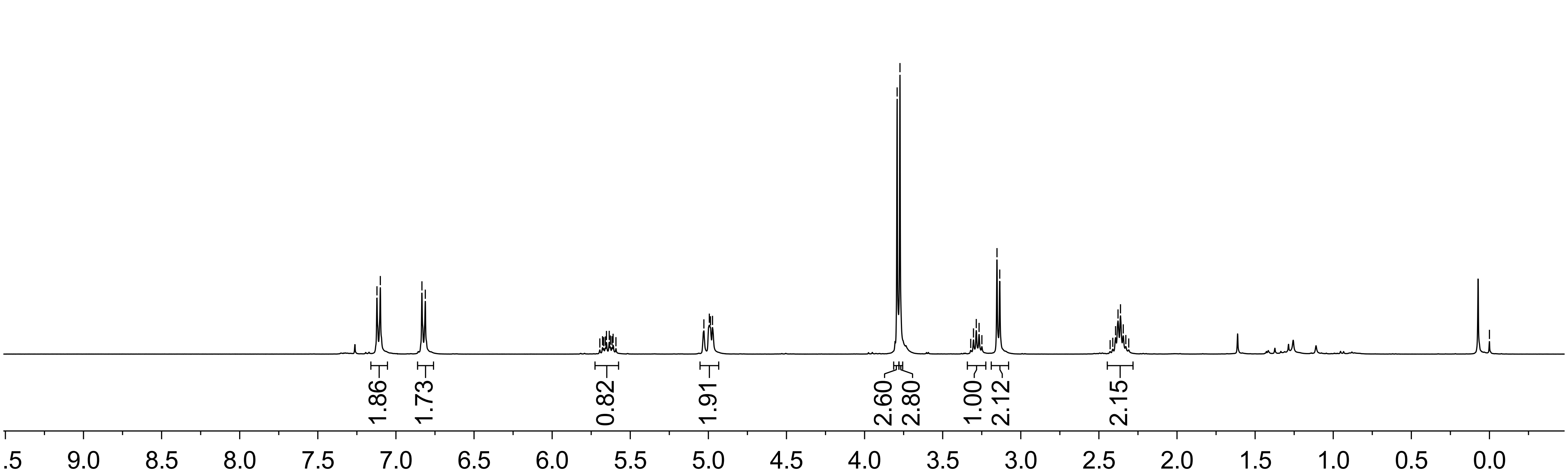

Gan-14-120-2P C13 CDCl3
2015-12-14 100MHz

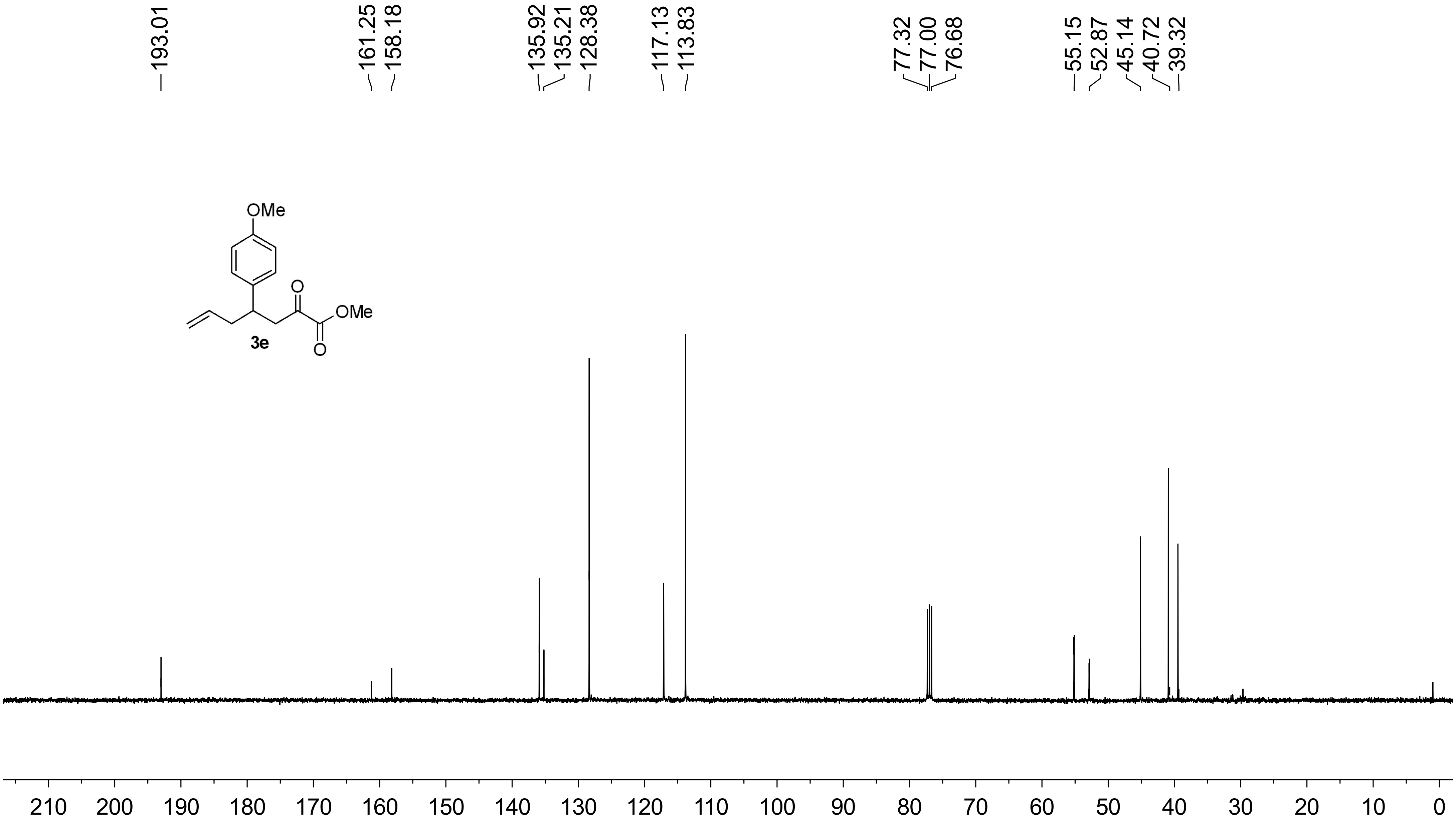

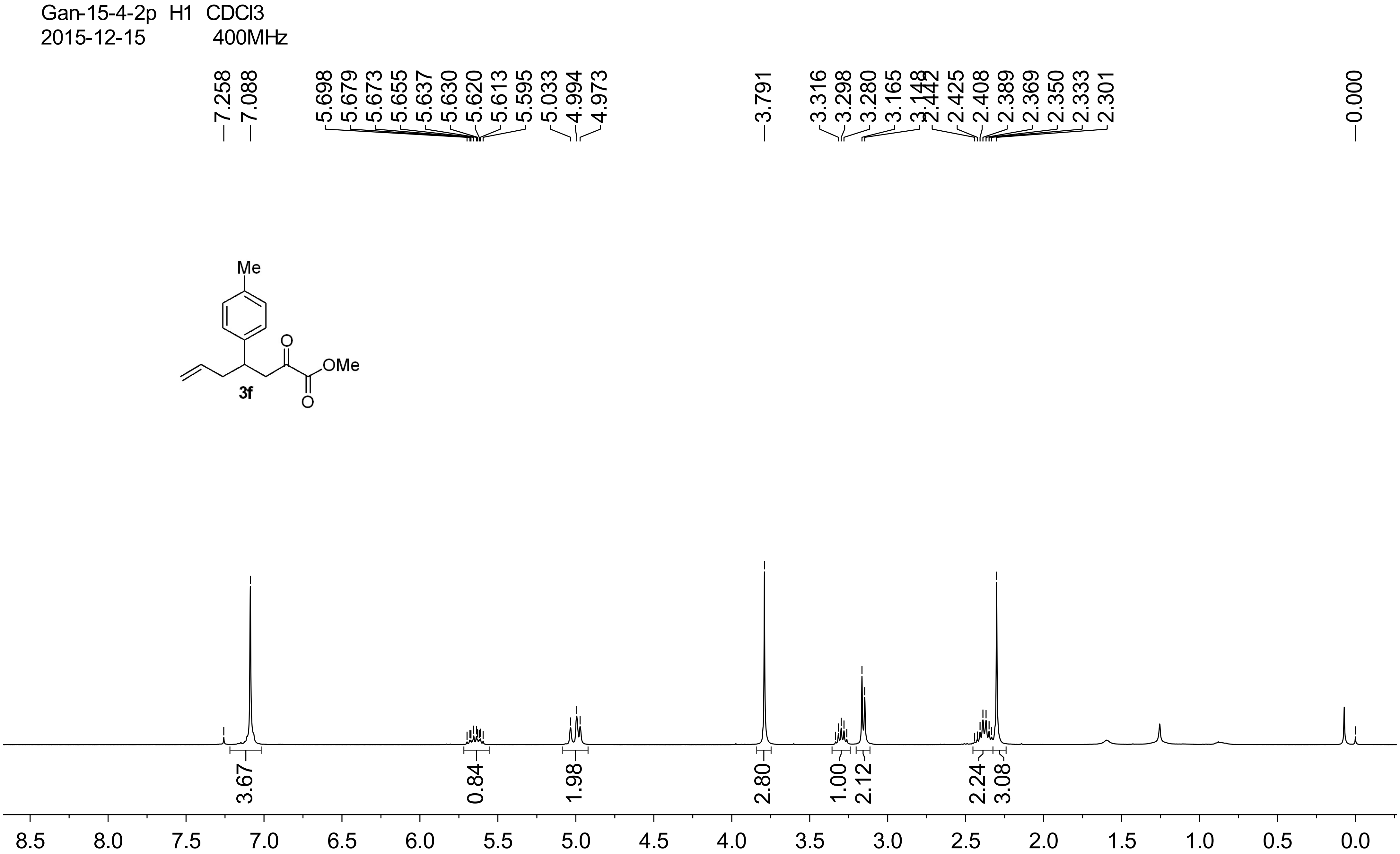

Gan-15-4-2p H1 CDCl3
2015-12-15 400MHz
7.258
7.088
5.698
5.679
5.673
5.655
5.637
5.630
5.620
5.613
5.595
5.033
4.994
4.973
3.791
3.316
3.298
3.280
3.165
3.148
2.442
2.425
2.408
2.389
2.369
2.350
2.333
2.301
0.000
Me
O
OMe
O
3f
3.67
0.84
1.98
2.80
1.00
2.12
2.24
3.08
8.5
8.0
7.5
7.0
6.5
6.0
5.5
5.0
4.5
4.0
3.5
3.0
2.5
2.0
1.5
1.0
0.5
0.0

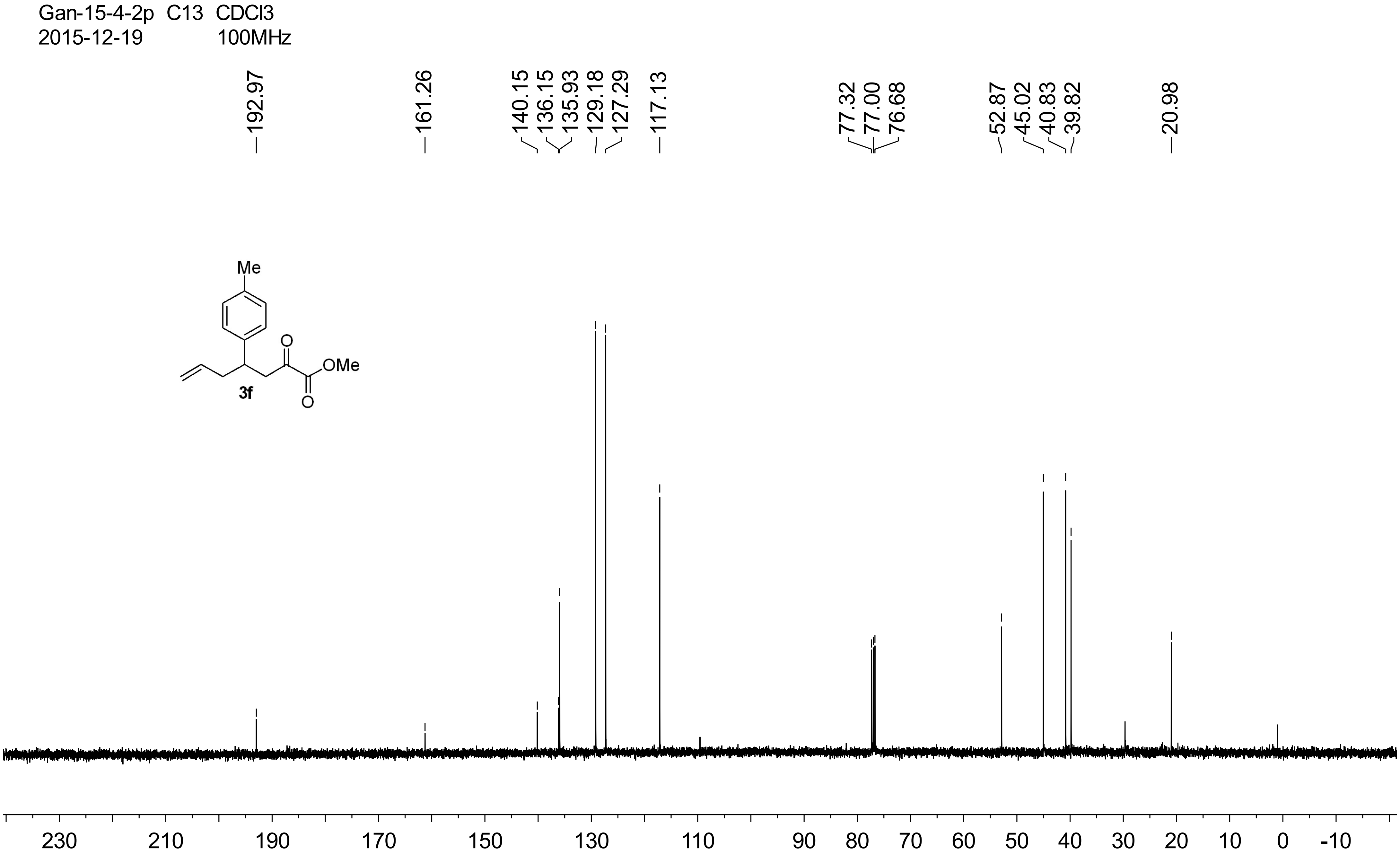

Gan-15-4-2p C13 CDCl3
2015-12-19 100MHz
192.97
161.26
140.15
136.15
135.93
129.18
127.29
117.13
77.32
77.00
76.68
52.87
45.02
40.83
39.82
20.98
Me
O
OMe
O
3f
230
210
190
170
150
130
110
90
80
70
60
50
40
30
20
10
0
-10

Gan-15-14-1p H1 CDCl3
2015-12-15 400MHz

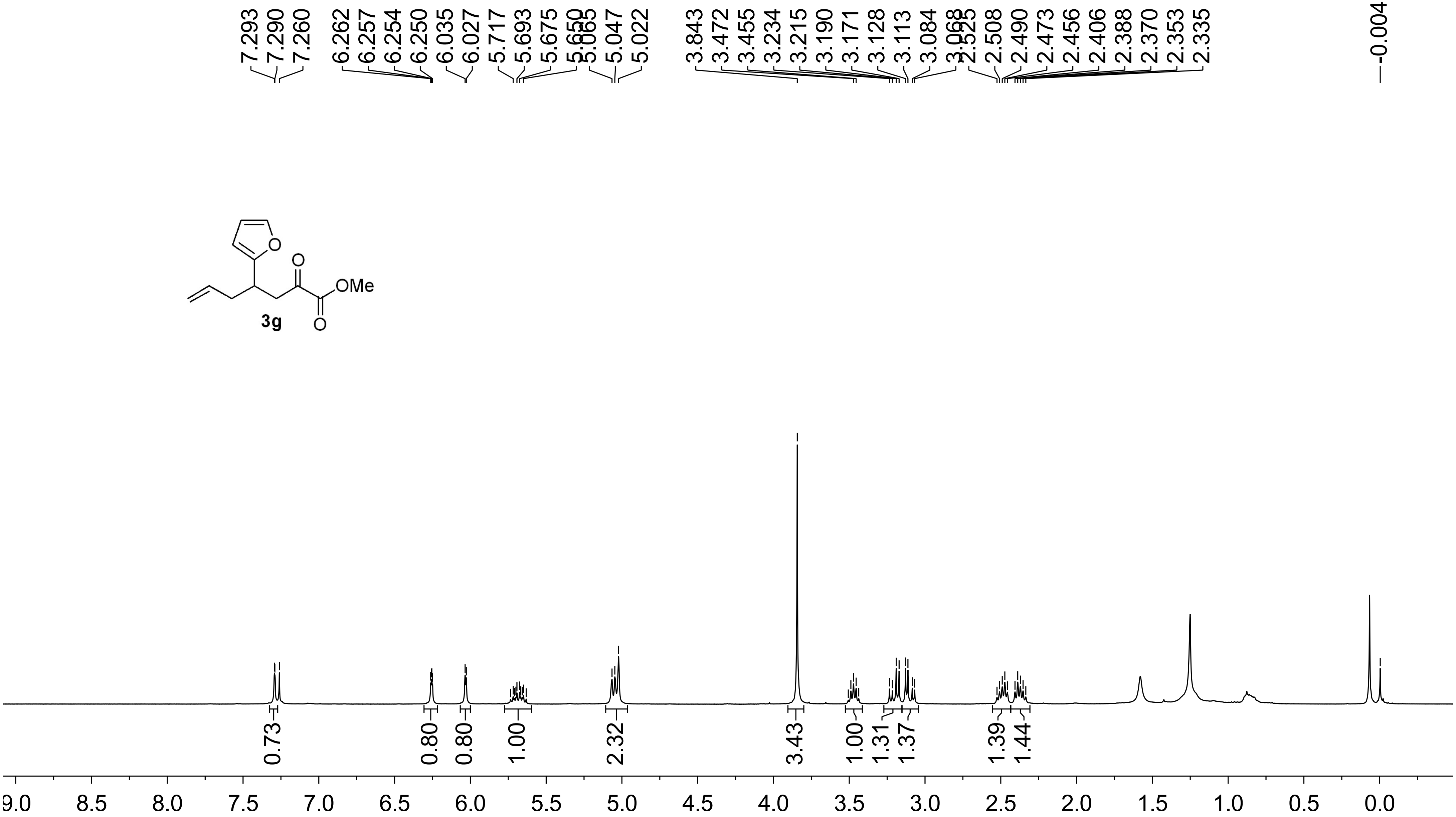

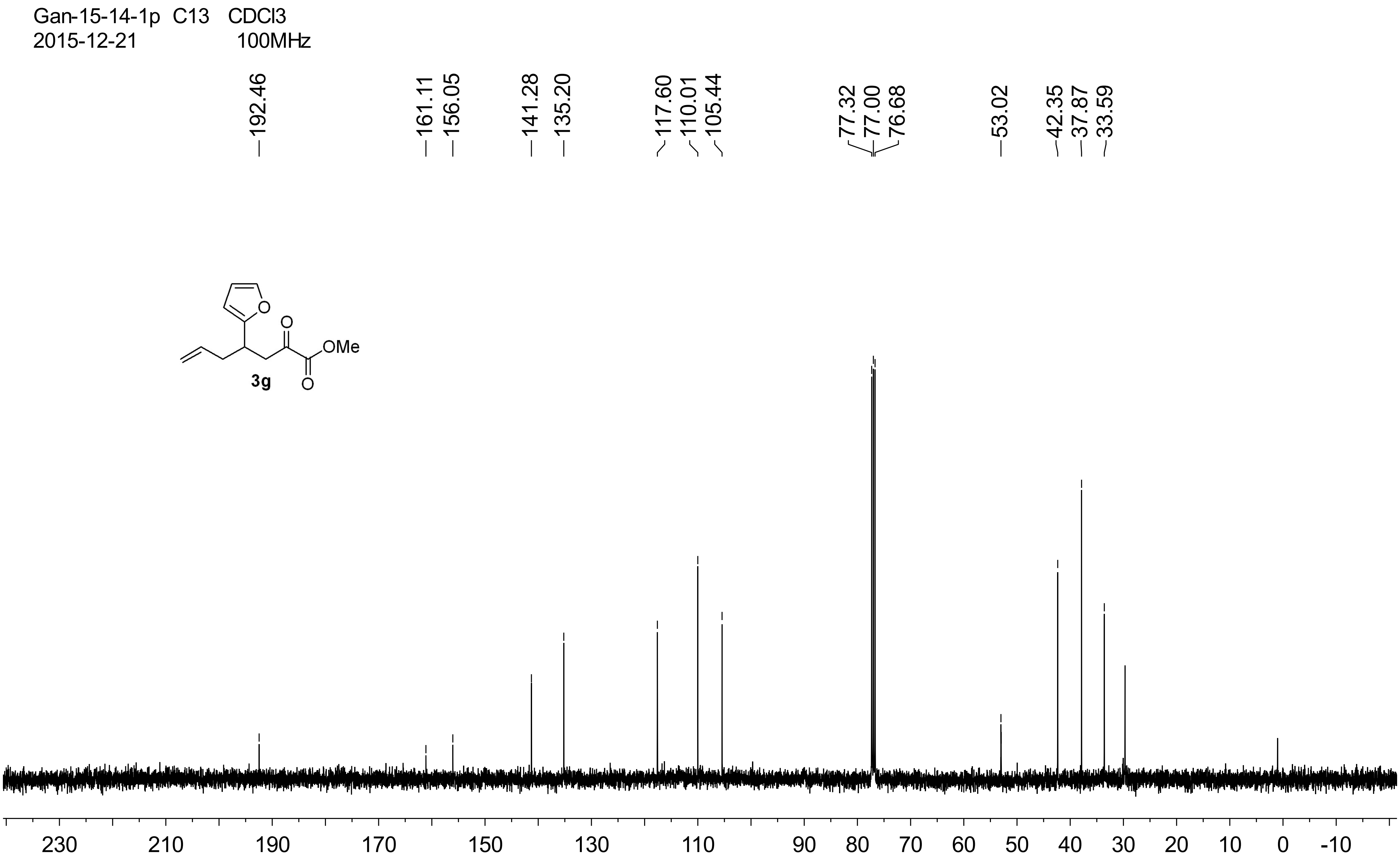
Gan-15-14-1p C13 CDCl3
2015-12-21 100MHz
192.46
161.11
156.05
141.28
135.20
117.60
110.01
105.44
77.32
77.00
76.68
53.02
42.35
37.87
33.59
O
O
OMe
O
3g
230
210
190
170
150
130
110
90
80
70
60
50
40
30
20
10
0
-10

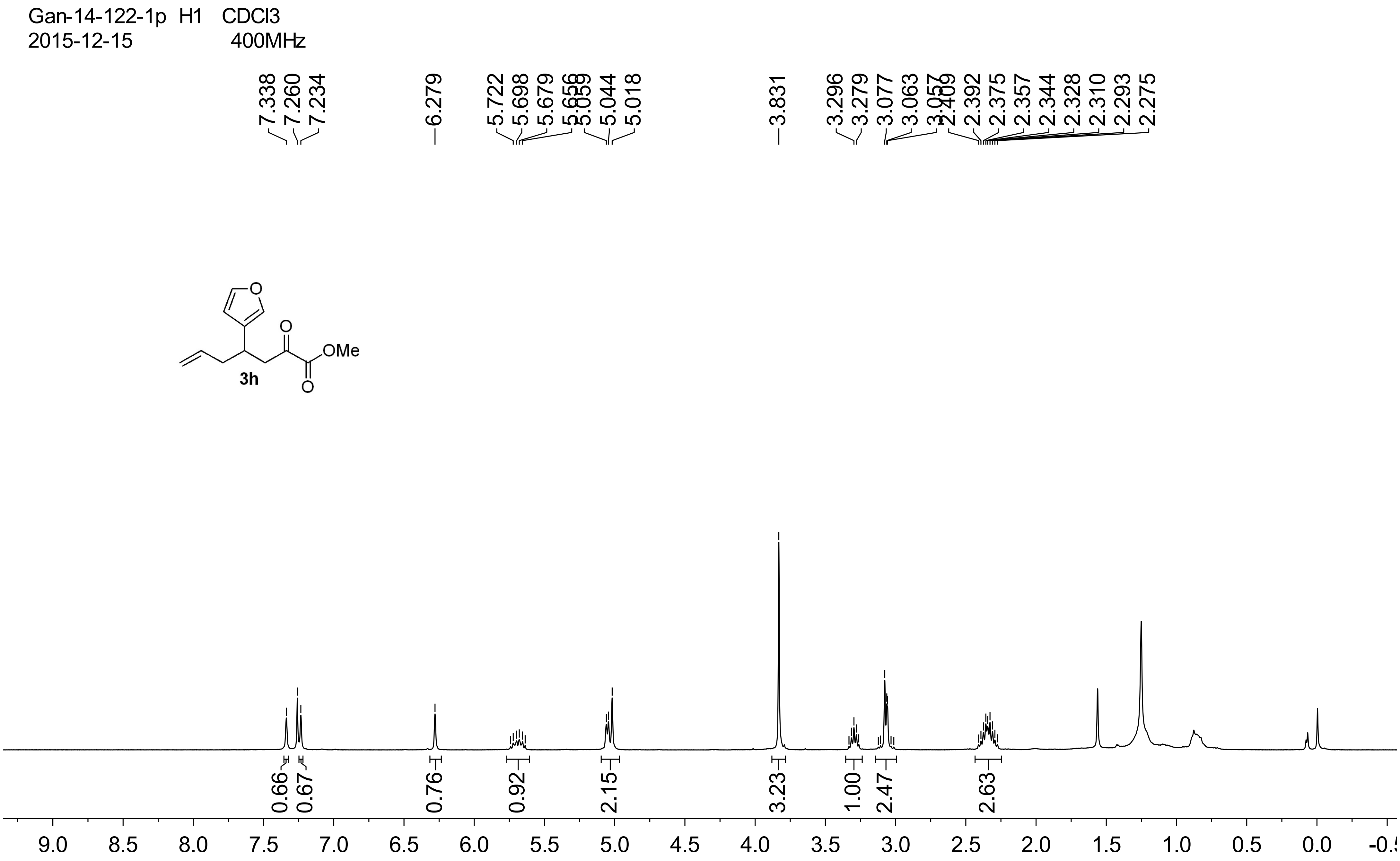

Gan-14-122-1p H1 CDCl3
2015-12-15 400MHz
7.338
7.260
7.234
6.279
5.722
5.698
5.679
5.656
5.059
5.044
5.018
3.831
3.296
3.279
3.077
3.063
3.057
2.409
2.392
2.375
2.357
2.344
2.328
2.310
2.293
2.275
OMe
3h
0.66
0.67
0.76
0.92
2.15
3.23
1.00
2.47
2.63
9.0
8.5
8.0
7.5
7.0
6.5
6.0
5.5
5.0
4.5
4.0
3.5
3.0
2.5
2.0
1.5
1.0
0.5
0.0

Gan-14-122-1p C13 CDCl3
2015-12-15 100MHz

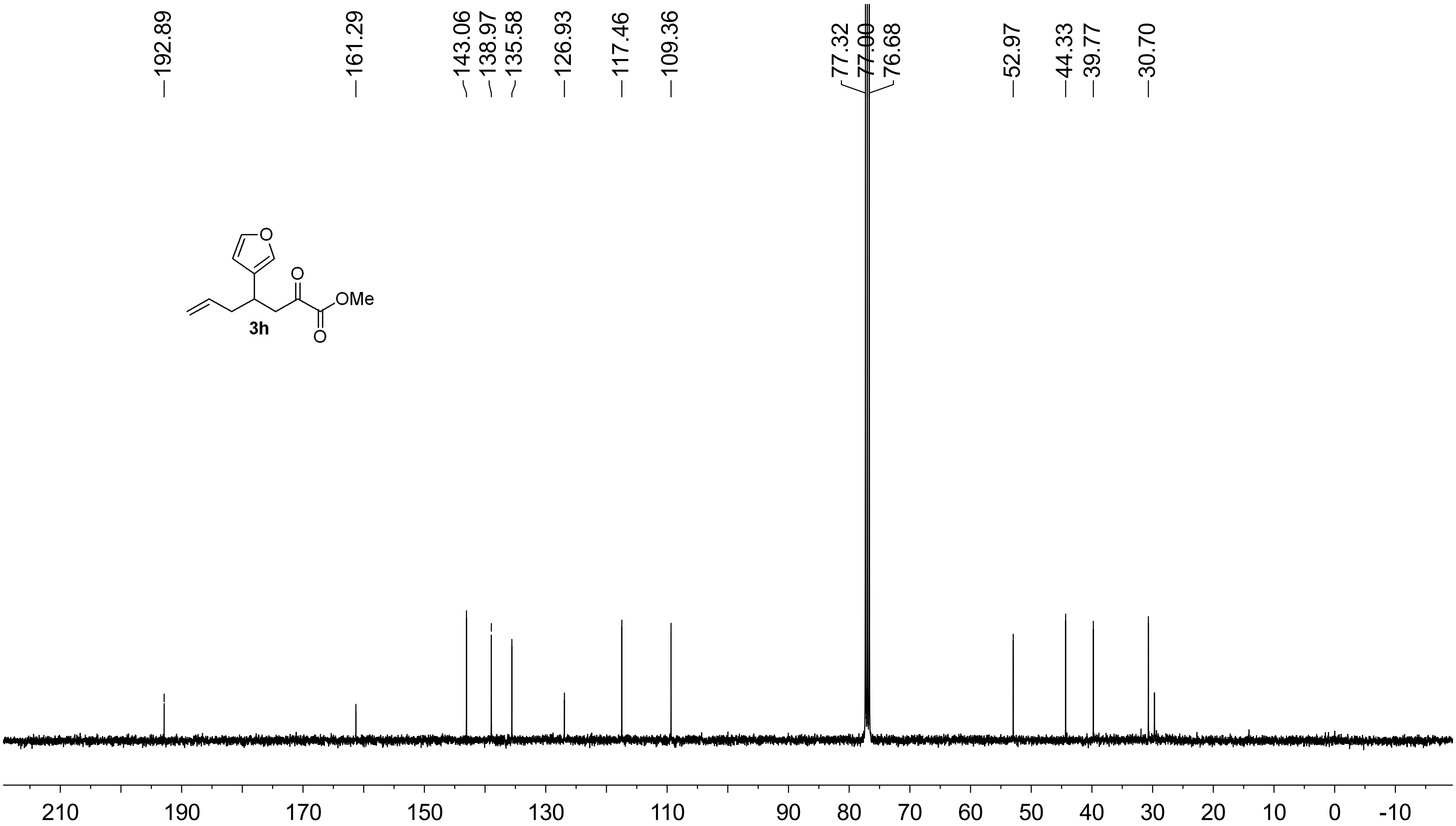

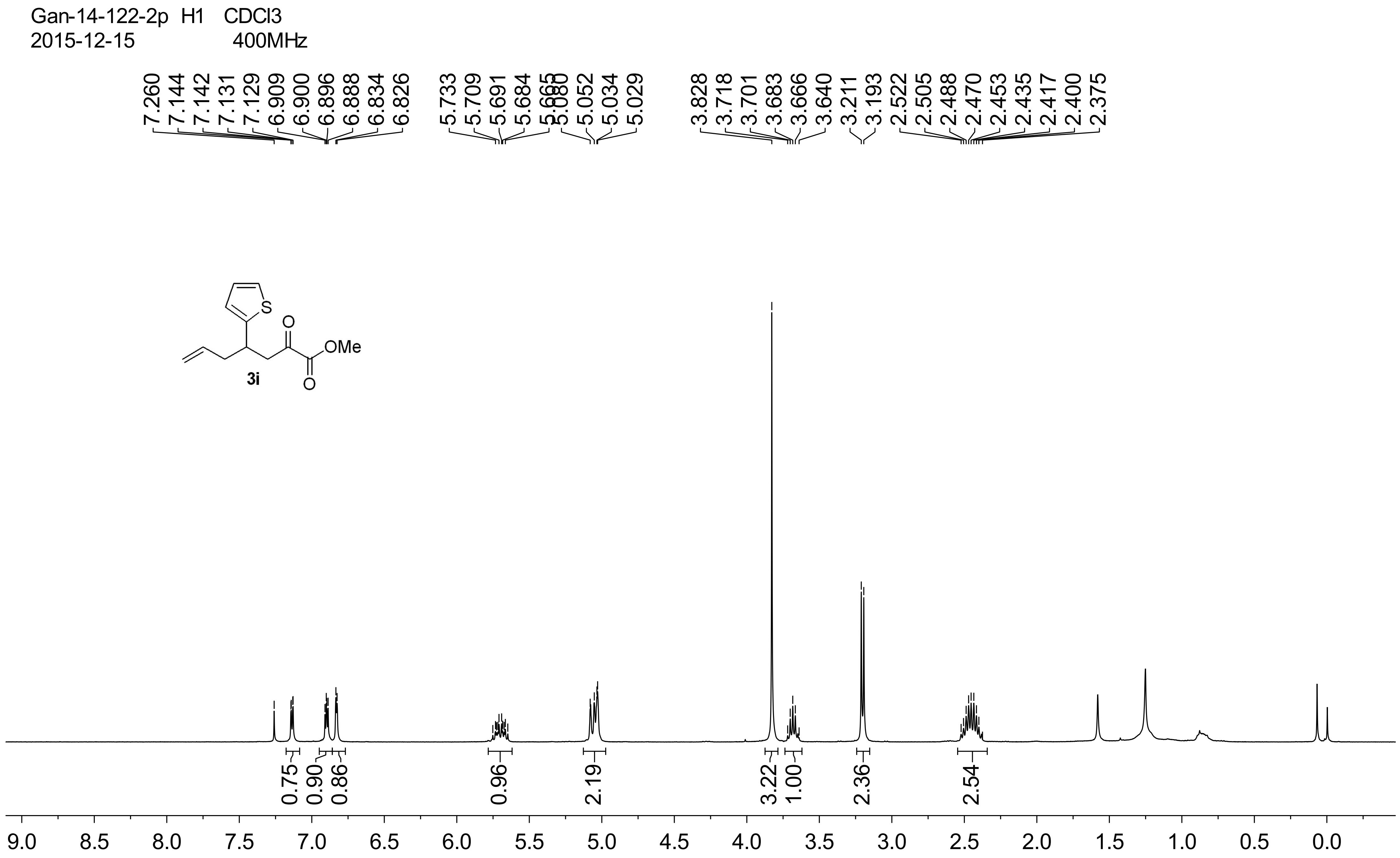
Gan-14-122-2p H1 CDCl3
2015-12-15 400MHz
7.260 7.144 7.142 7.131 7.129 6.909 6.900 6.896 6.888 6.834 6.826
5.733 5.709 5.691 5.684 5.665 5.080 5.052 5.034 5.029
3.828 3.718 3.701 3.683 3.666 3.640 3.211 3.193 2.522 2.505 2.488 2.470 2.453 2.435 2.417 2.400 2.375
S
O
OMe
O
3i
0.75 0.90 0.86 0.96 2.19 3.22 1.00 2.36 2.54
9.0 8.5 8.0 7.5 7.0 6.5 6.0 5.5 5.0 4.5 4.0 3.5 3.0 2.5 2.0 1.5 1.0 0.5 0.0

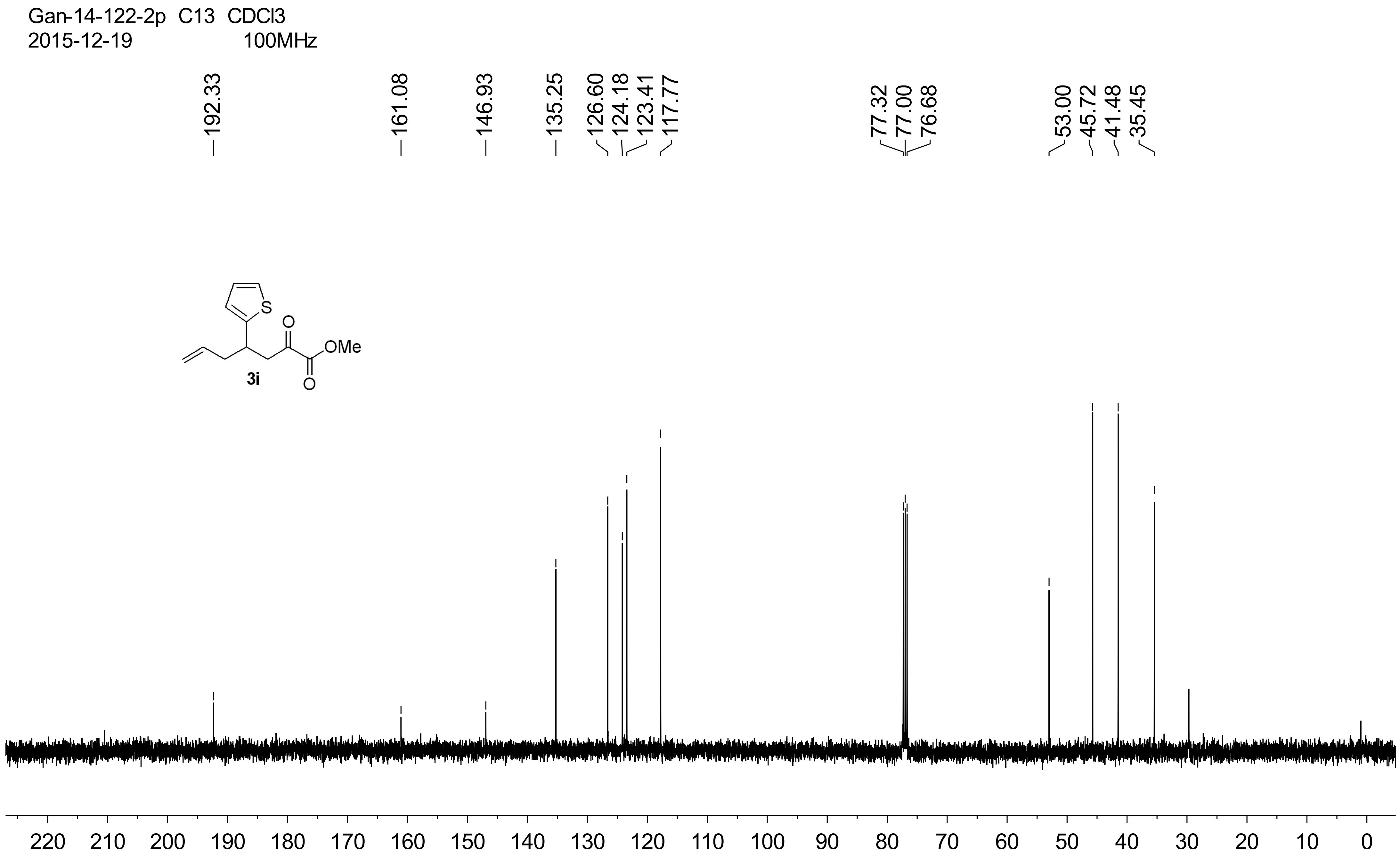
Gan-14-122-2p C13 CDCl3
2015-12-19 100MHz
192.33
161.08
146.93
135.25
126.60
124.18
123.41
117.77
77.32
77.00
76.68
53.00
45.72
41.48
35.45
S
O
OMe
O
3i
220
210
200
190
180
170
160
150
140
130
120
110
100
90
80
70
60
50
40
30
20
10
0

Gan-14-121-2p H1 CDCl3
2015-12-15 400MHz

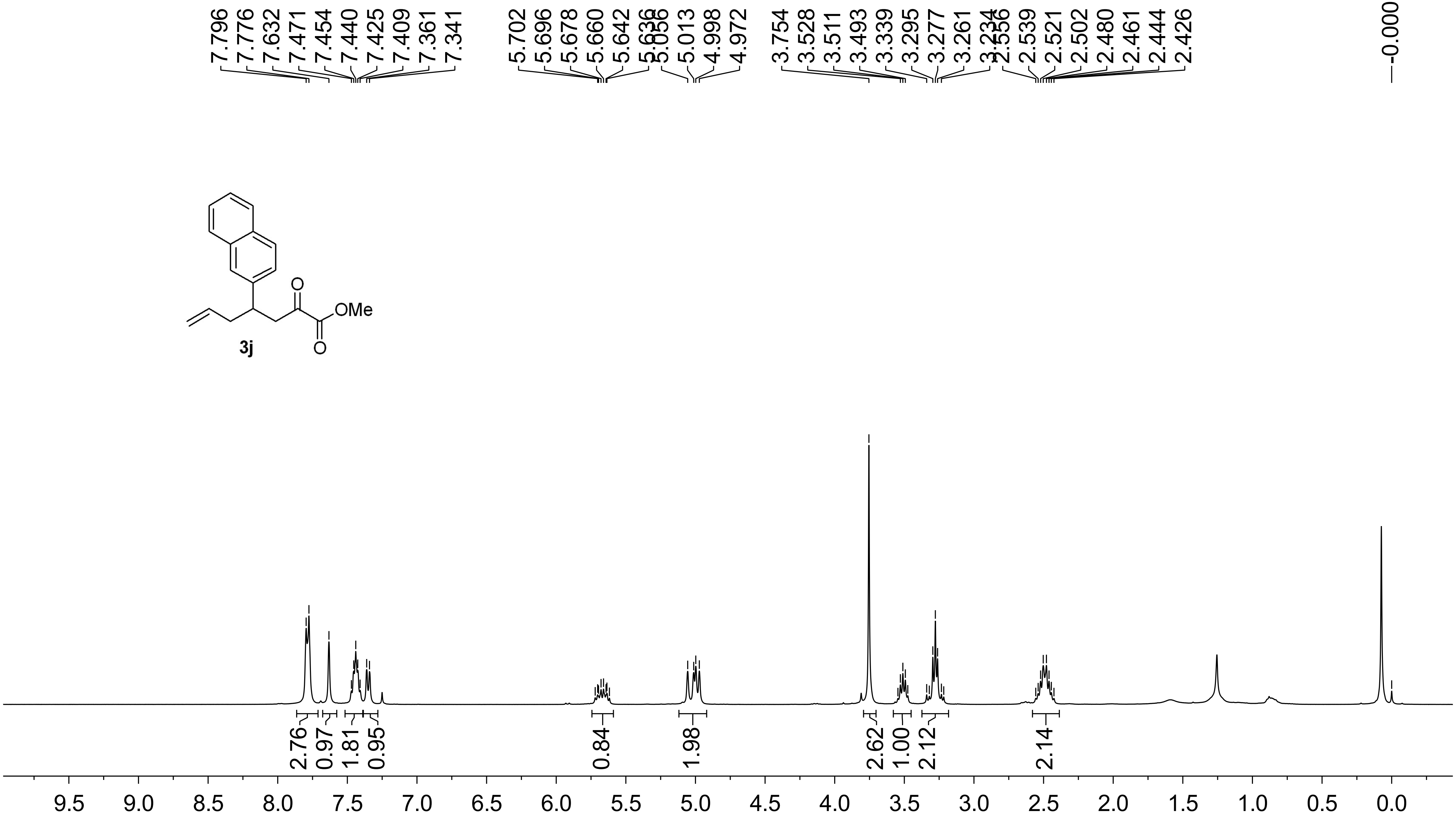

Gan-14-121-2p C13 CDCl3
2015-12-15 100MHz

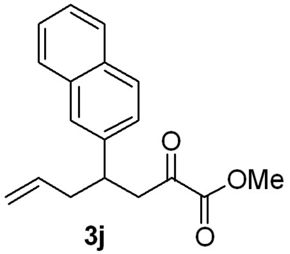

3j

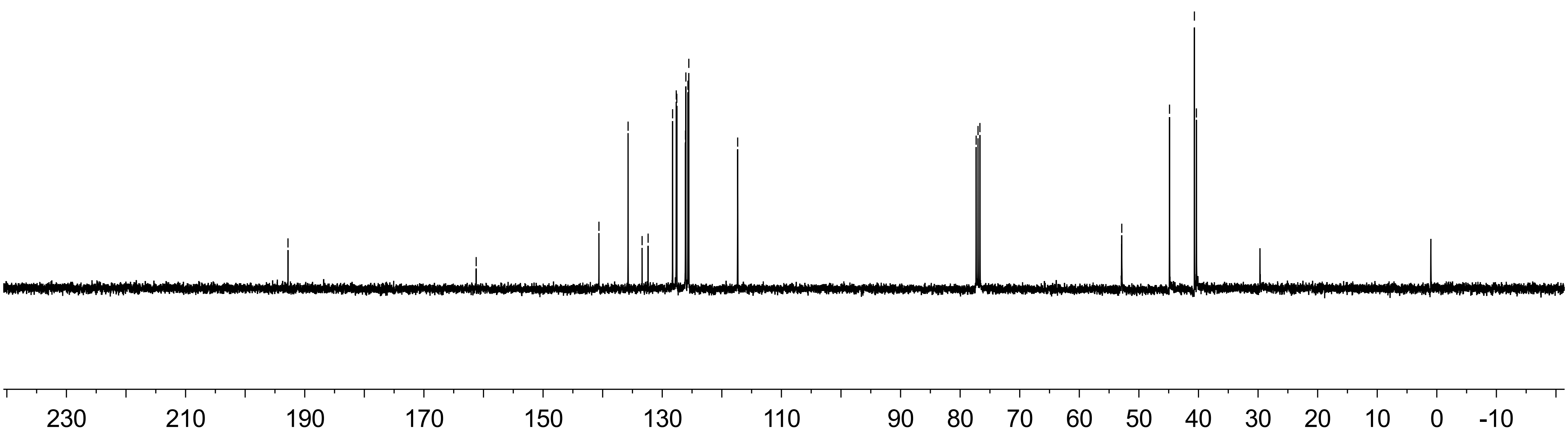

Gan-15-101-p H1 CDCl3
2016-1-13 400MHz

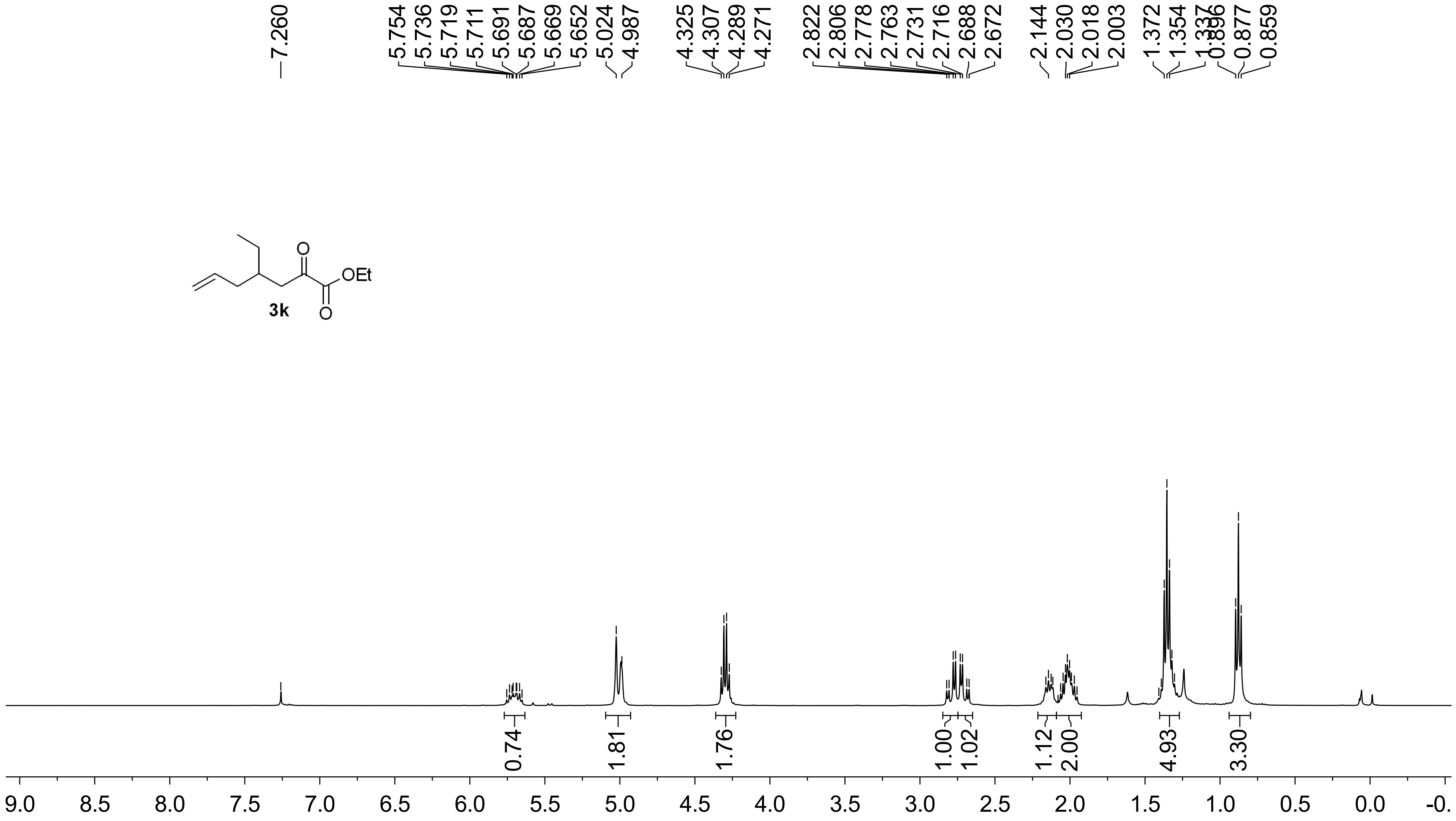

Gan-15-101-p C13 CDCl3
2016-1-16 100MHz

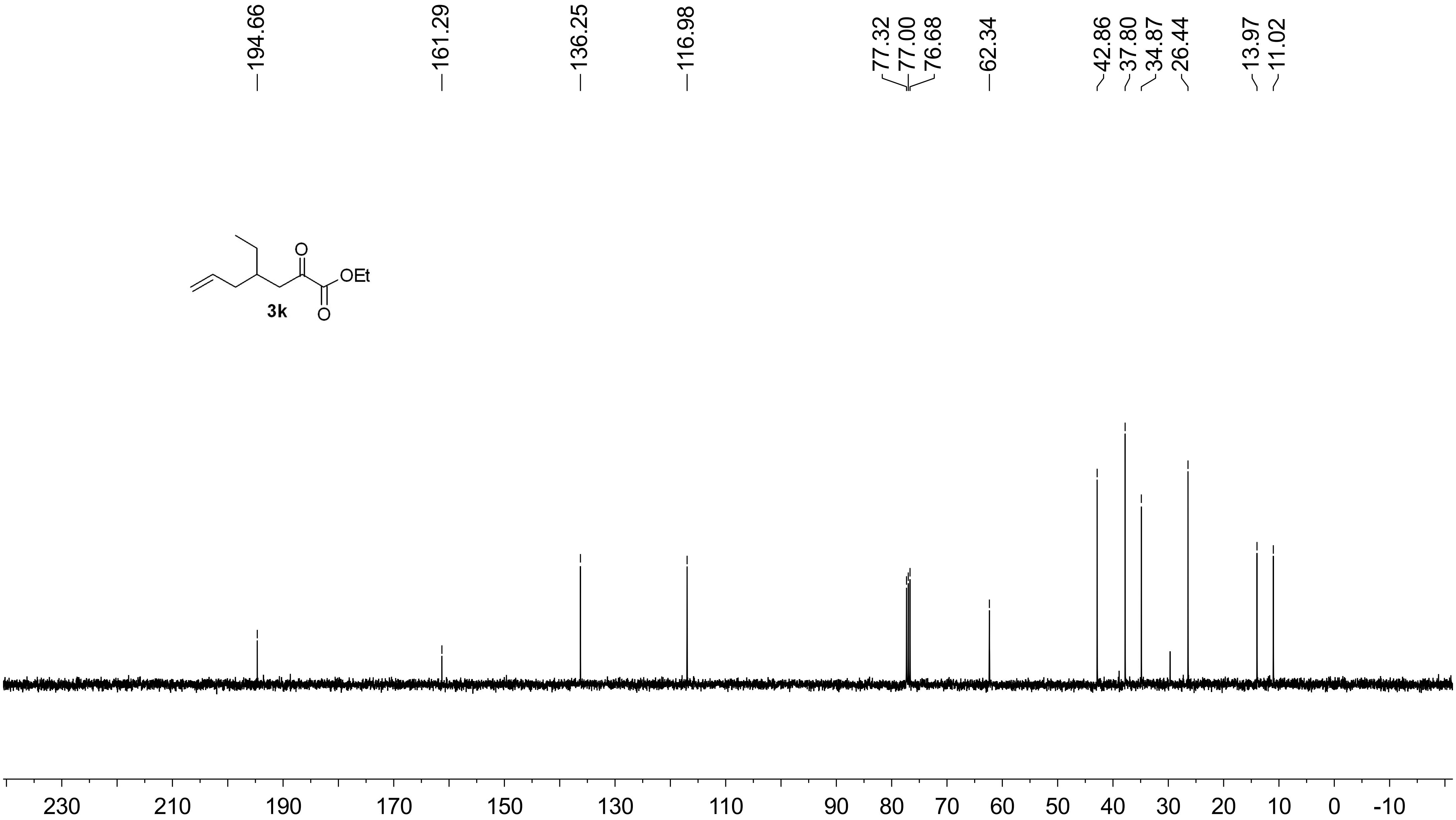

Gan-15-105-p H1 CDCl3
2016-1-15 400MHz

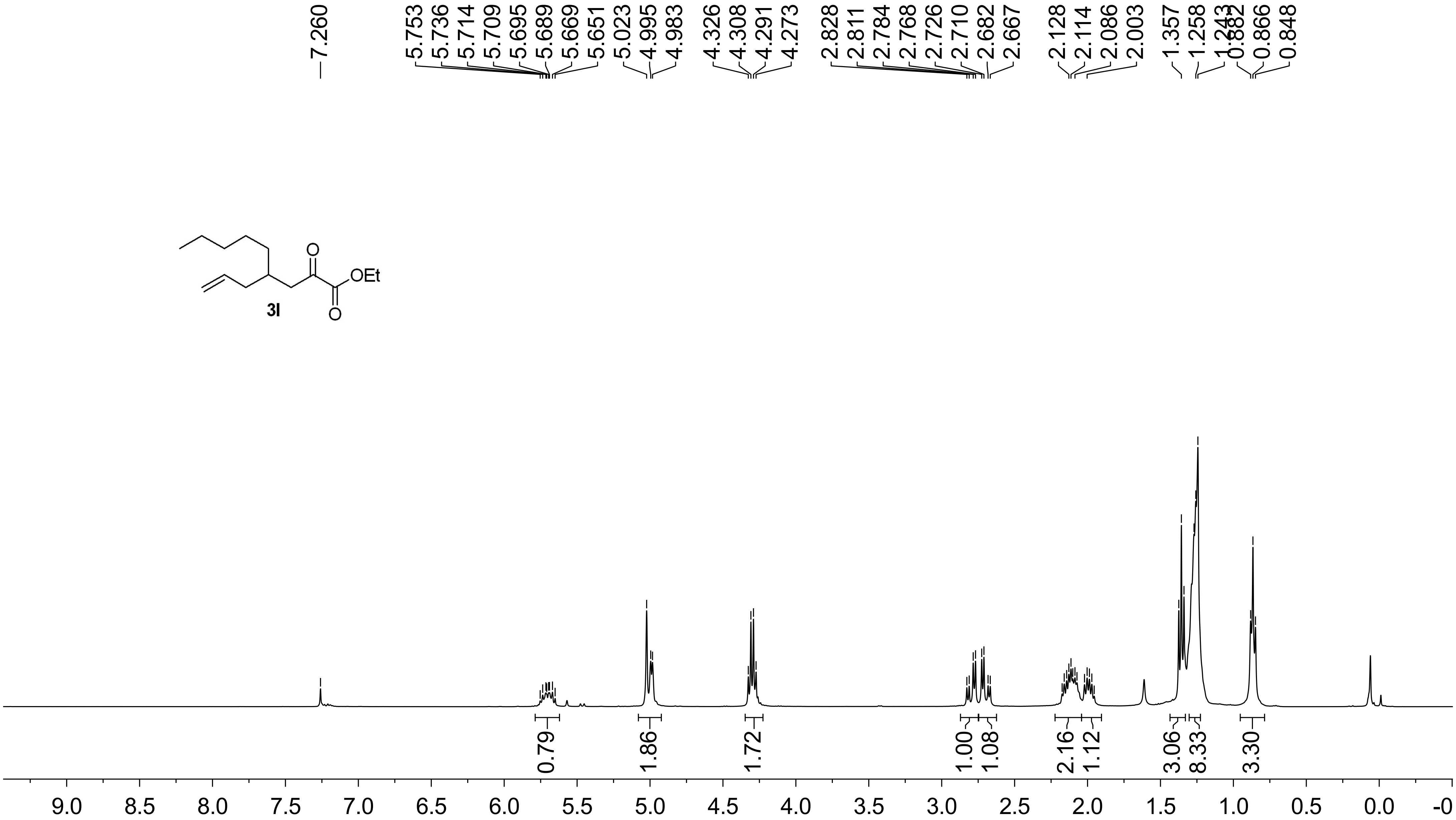

Gan-15-105-p C13 CDCl3
2016-1-16 100MHz

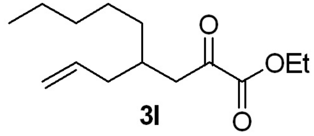

3l

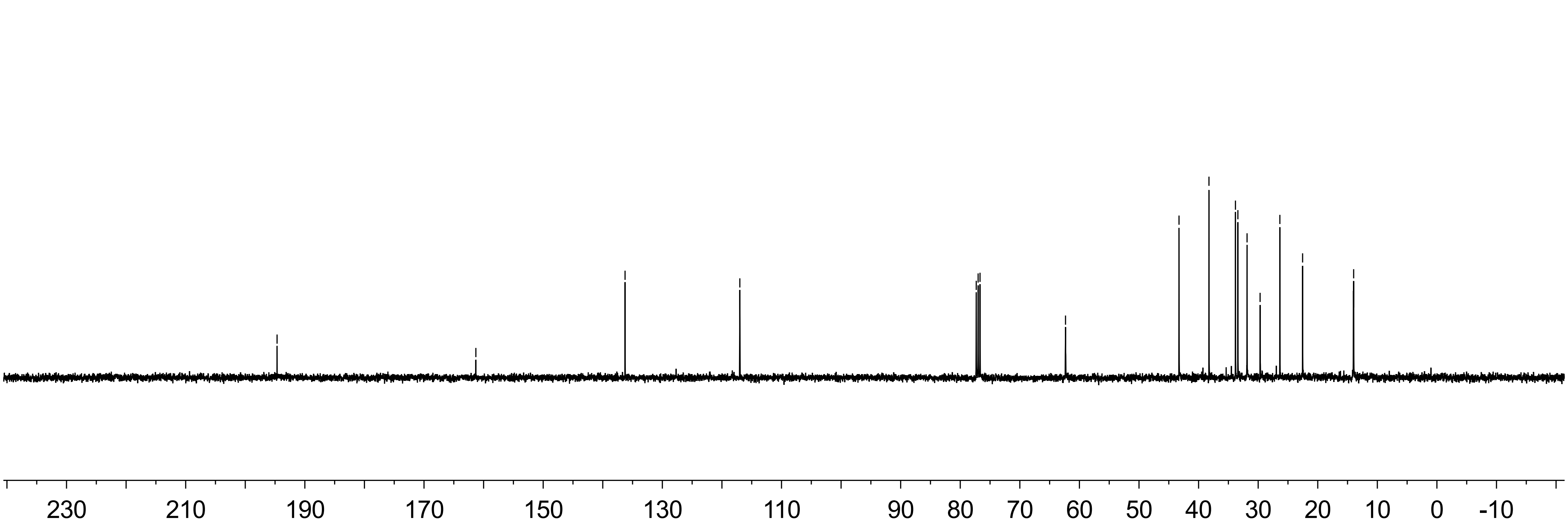

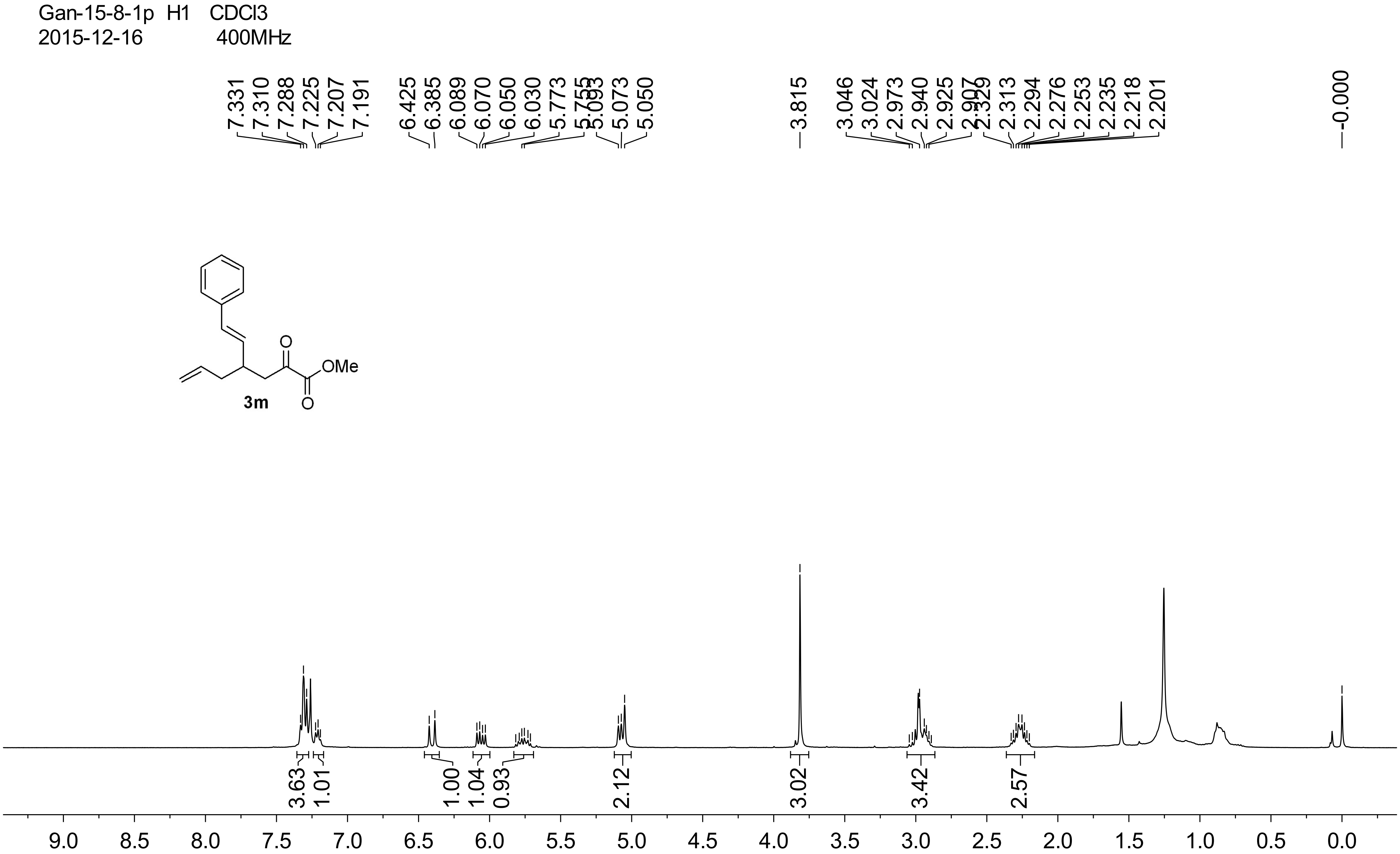
Gan-15-8-1p H1 CDCl3
2015-12-16 400MHz
7.331 7.310 7.288 7.225 7.207 7.191
6.425 6.385 6.089 6.070 6.050 6.030 5.773 5.755 5.093 5.073 5.050
3.815
3.046 3.024 2.973 2.940 2.925 2.907 2.329 2.313 2.294 2.276 2.253 2.235 2.218 2.201
-0.000
O
OMe
O
3m
3.63 1.01 1.00 1.04 0.93 2.12 3.02 3.42 2.57
9.0 8.5 8.0 7.5 7.0 6.5 6.0 5.5 5.0 4.5 4.0 3.5 3.0 2.5 2.0 1.5 1.0 0.5 0.0

Gan-15-8-1p C13 CDCl3
2015-12-16 100MHz

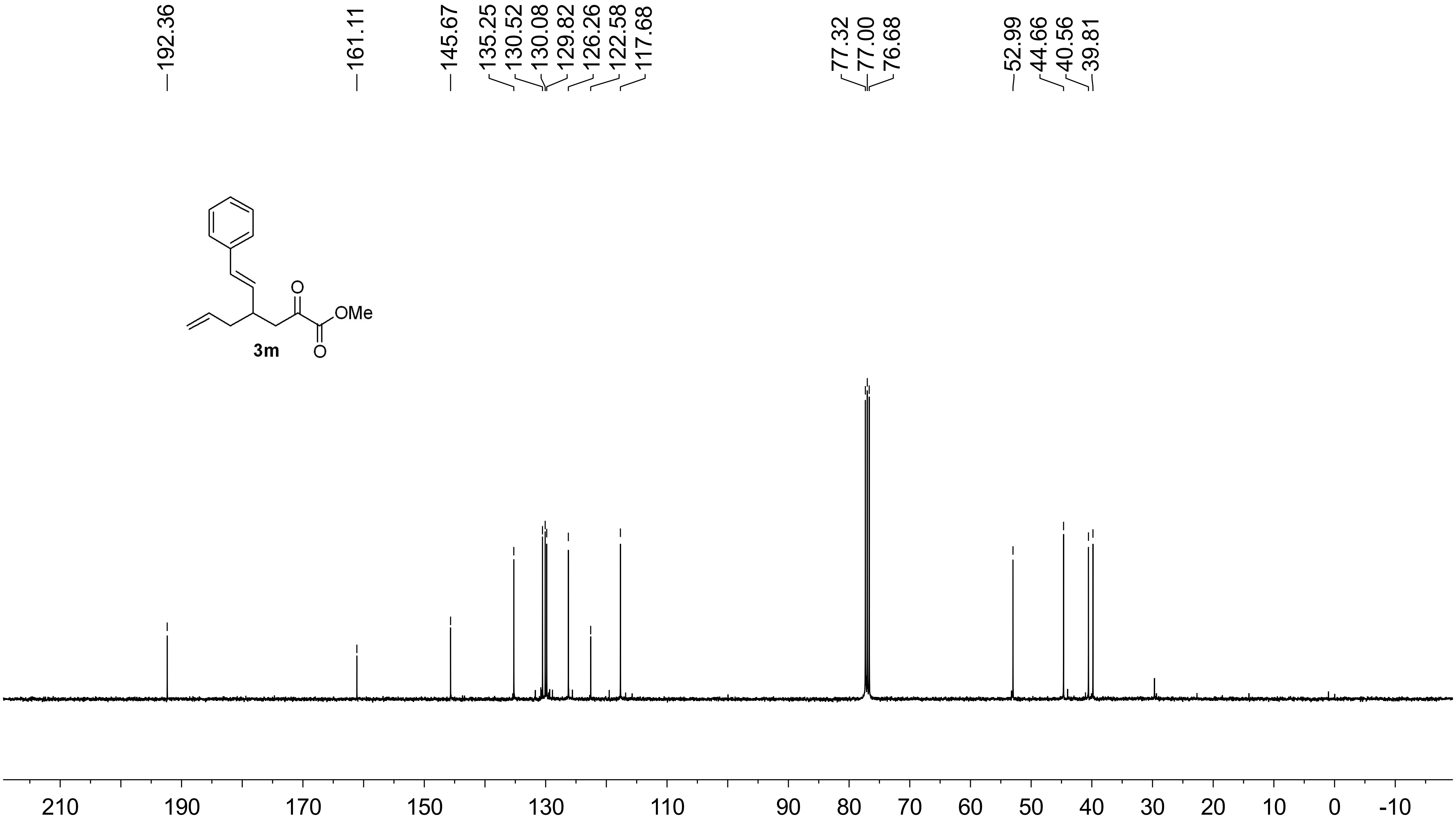

Gan-16-6-1p H1 CDCl3
2015-1-19 400MHz

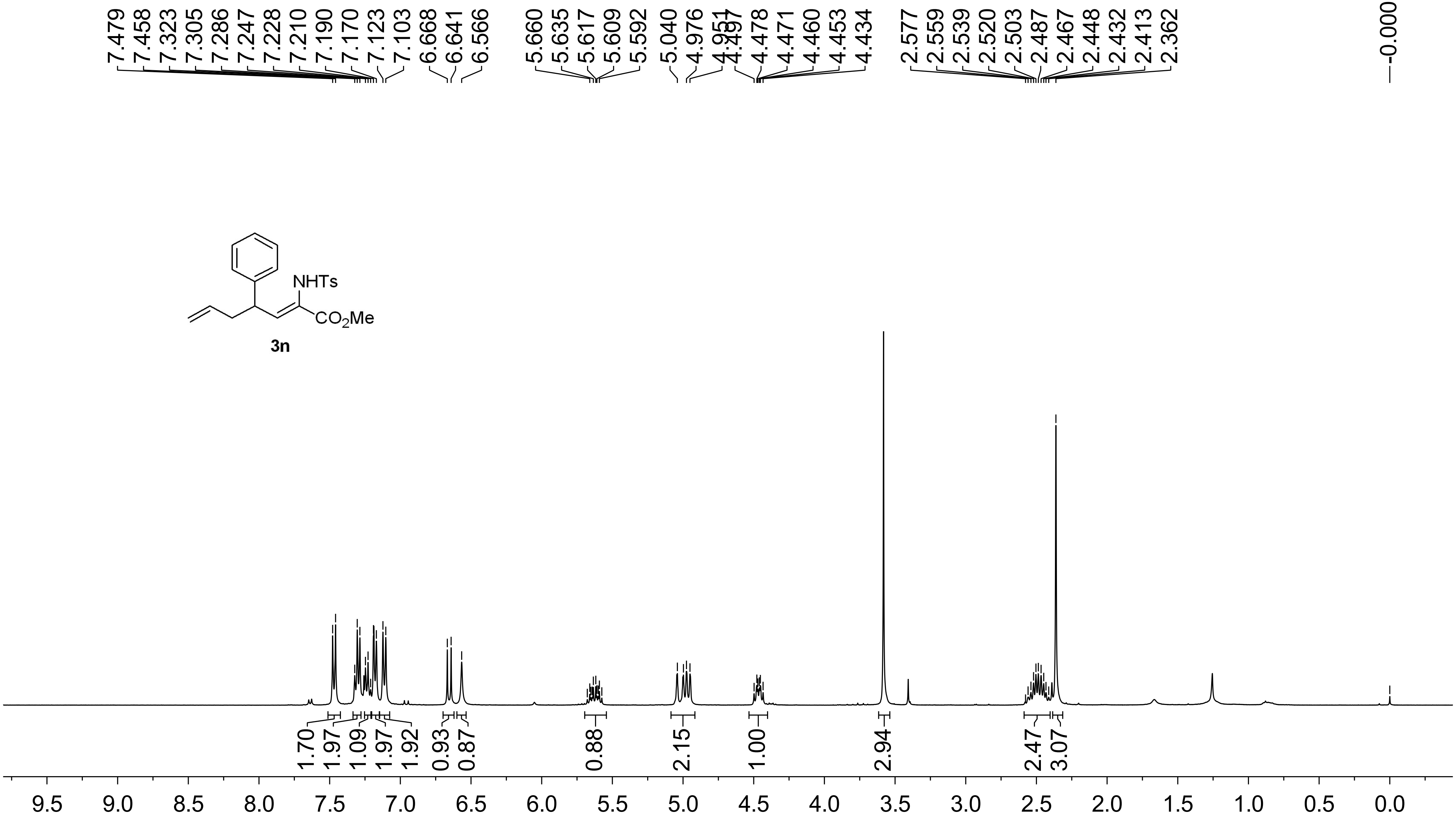

Gan-16-6-1p H1 CDCl3
2015-1-23 100MHz

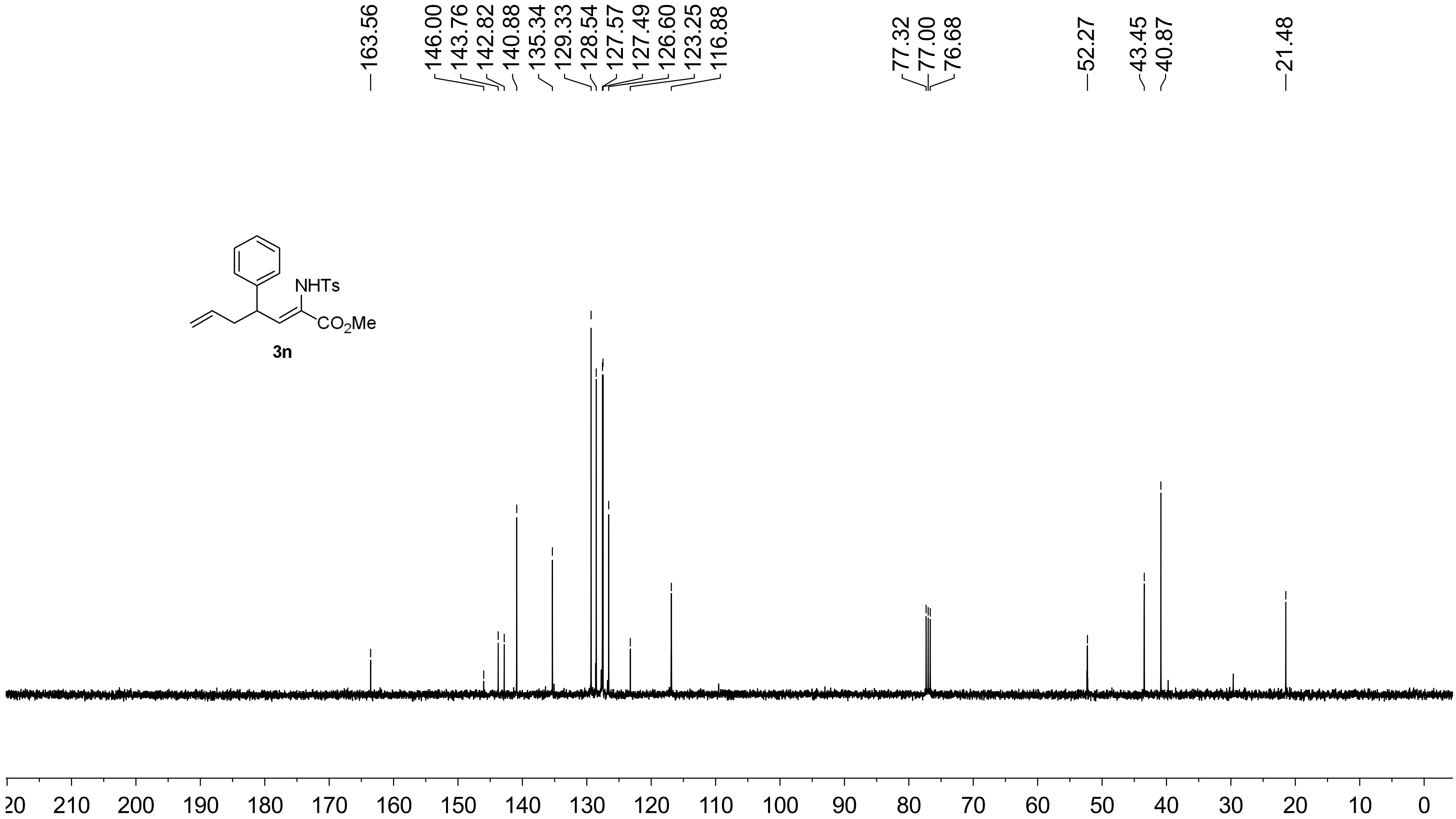

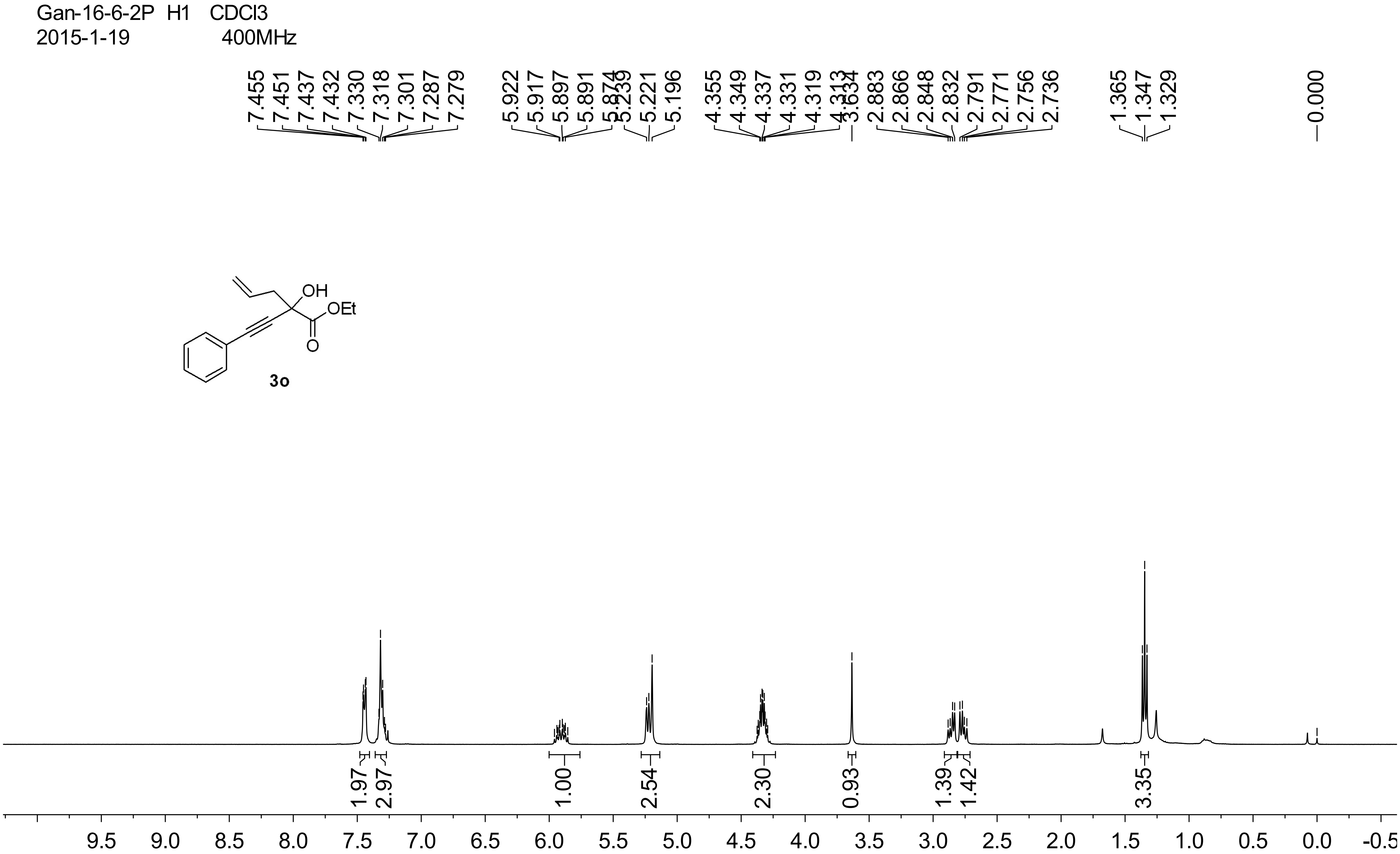
Gan-16-6-2P H1 CDCl3
2015-1-19 400MHz
7.455 7.451 7.437 7.432 7.330 7.318 7.301 7.287 7.279
5.922 5.917 5.897 5.891 5.874 5.239 5.221 5.196
4.355 4.349 4.337 4.331 4.319 4.313 3.634
2.883 2.866 2.848 2.832 2.791 2.771 2.756 2.736
1.365 1.347 1.329
0.000
OH
OEt
O
3o
1.97 2.97 1.00 2.54 2.30 0.93 1.39 1.42 3.35
9.5 9.0 8.5 8.0 7.5 7.0 6.5 6.0 5.5 5.0 4.5 4.0 3.5 3.0 2.5 2.0 1.5 1.0 0.5 0.0 -0.5

Gan-16-6-2P C13 CDCl3
2015-1-23 100MHz

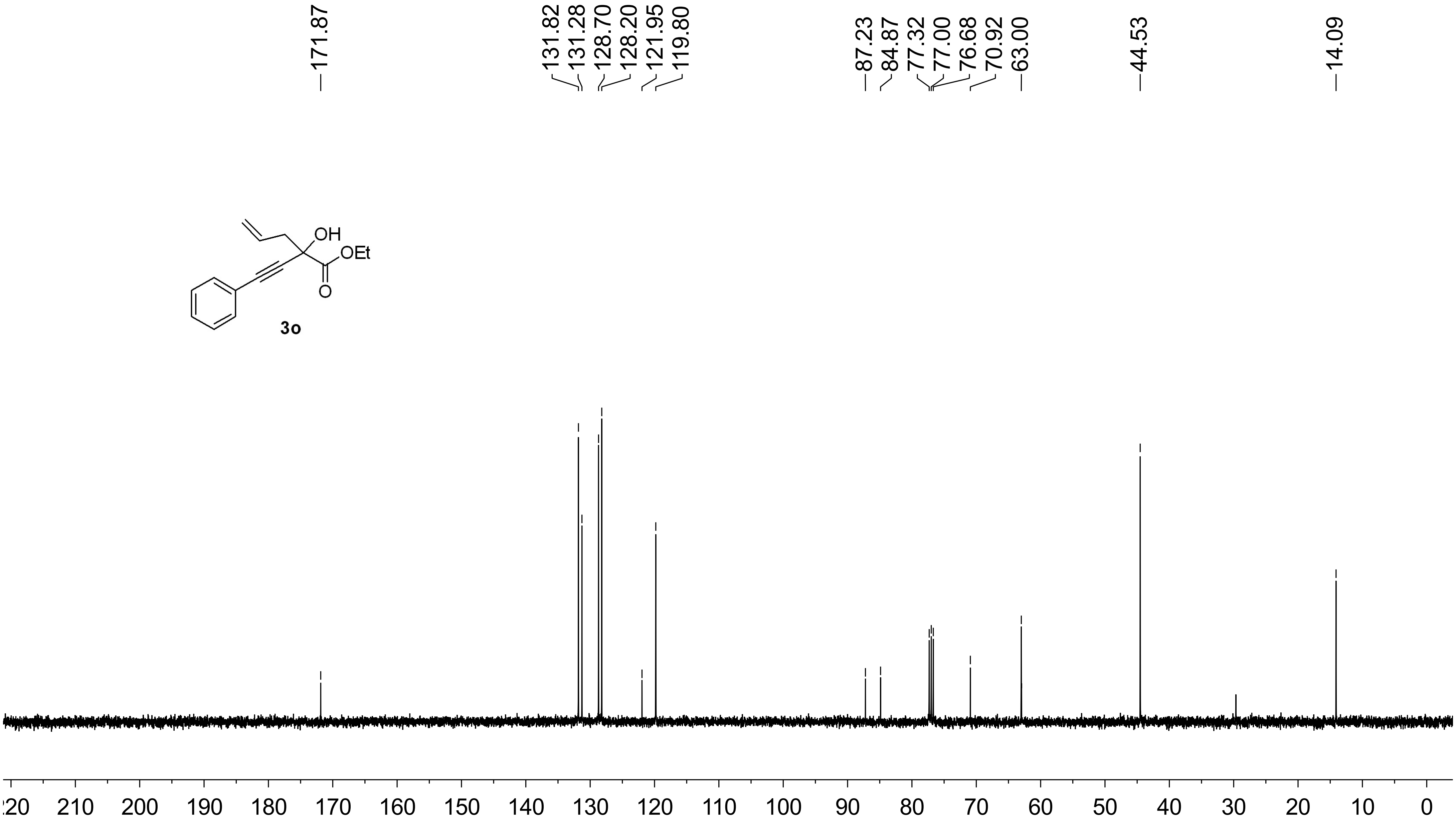

Gan-15-16-p1 H1 CDCl3
2015-12-15 400MHz

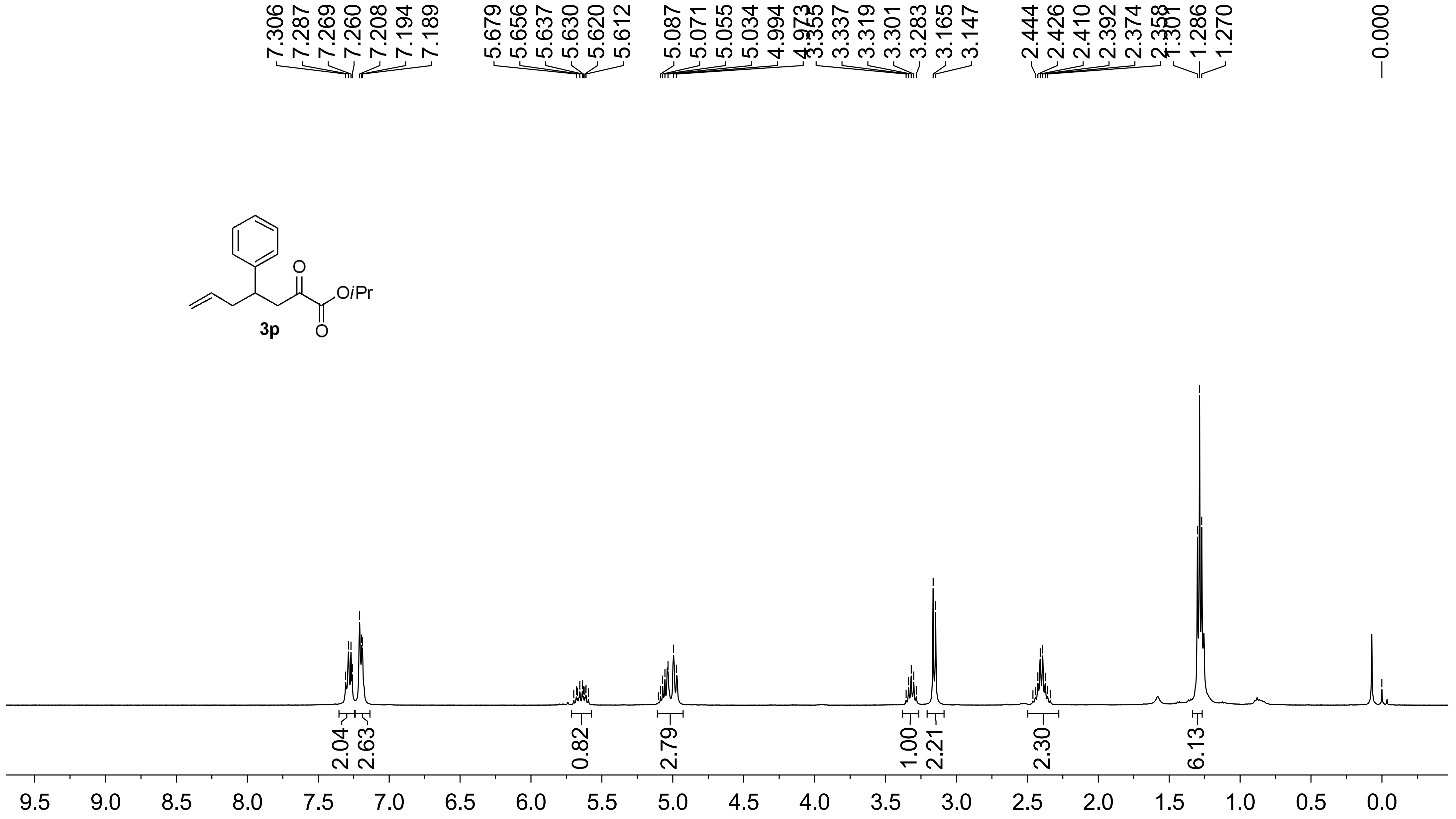

Gan-15-16-p1 C13 CDCl3
2015-12-29 100MHz

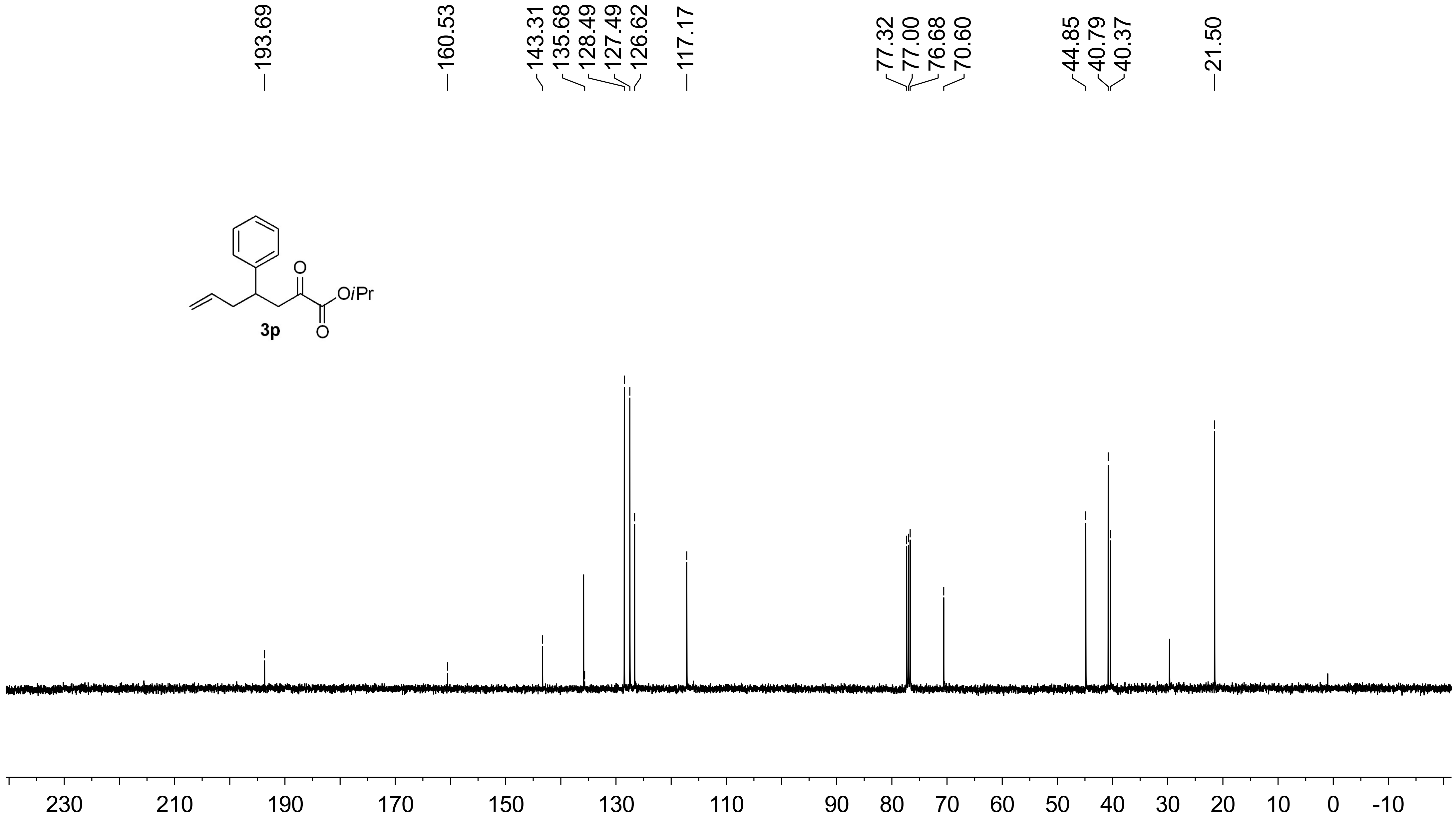

Gan-15-37-1p H1 CDCl3
2015-12-25 400MHz

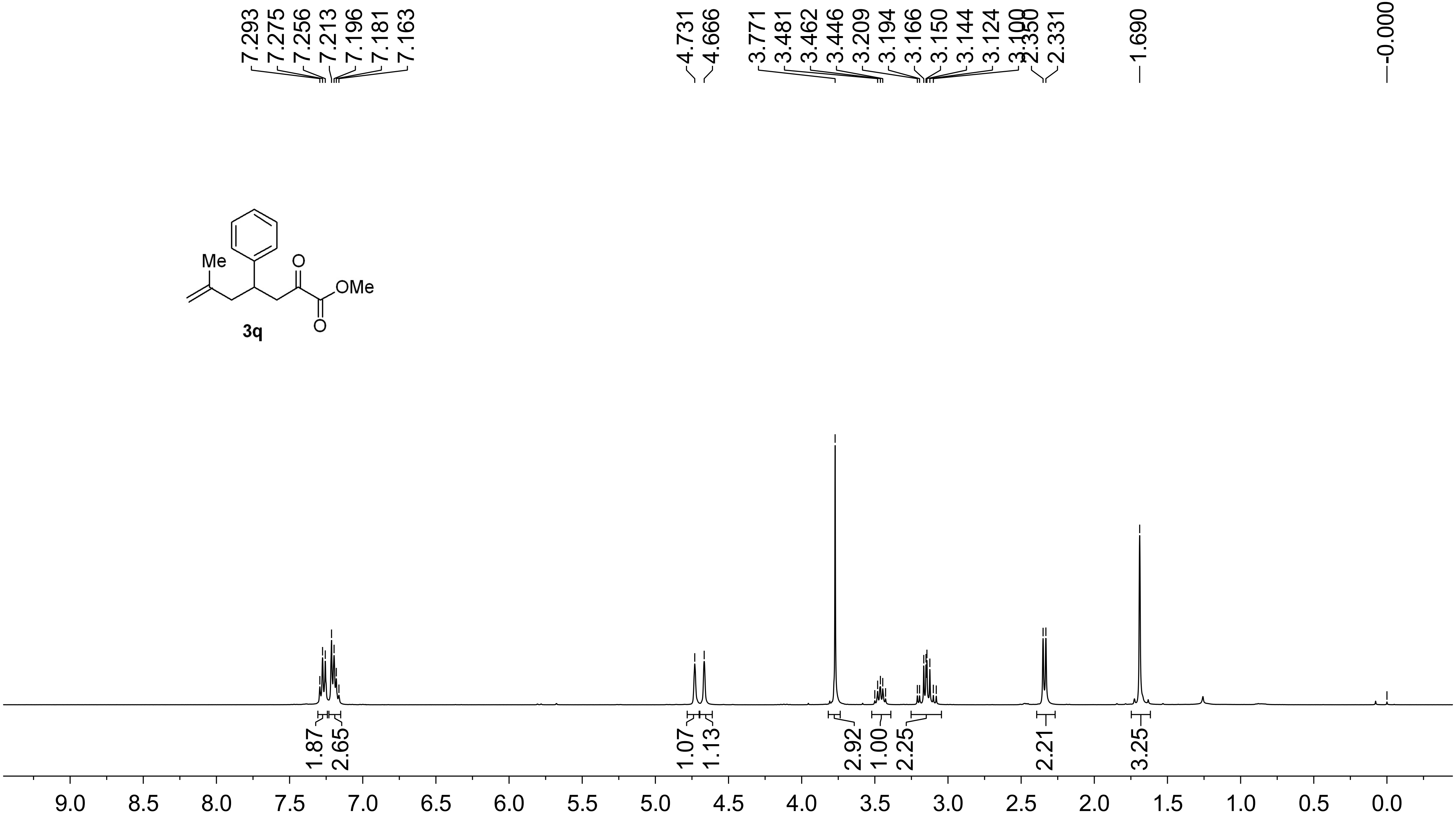

Gan-15-37-1p C13   CDCl3
2015-12-27   100MHz

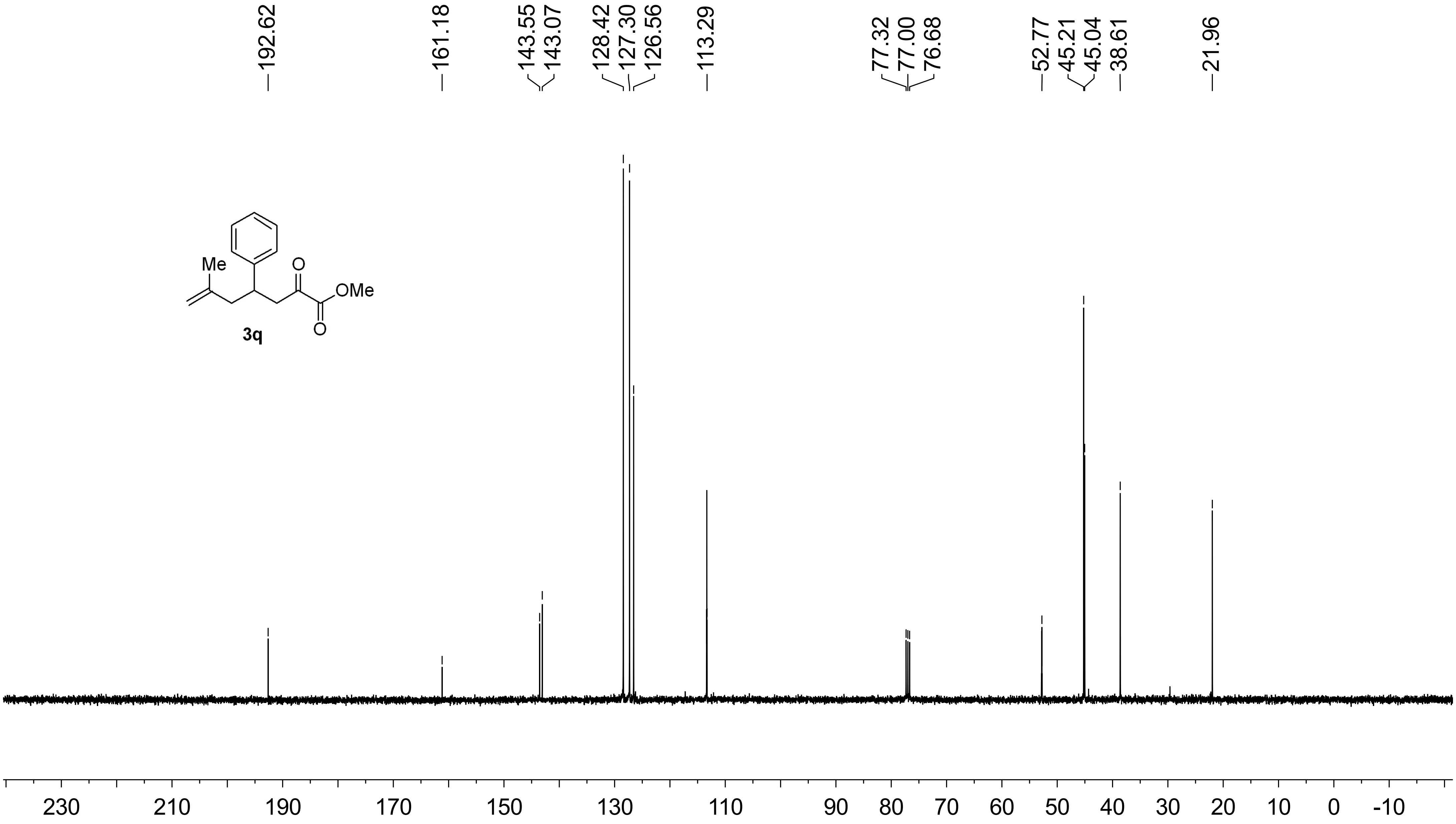

Gan-16-45-p H1 CDCl3
2016-3-14 100MHz

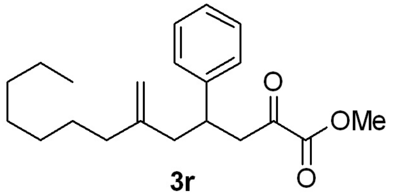

3r

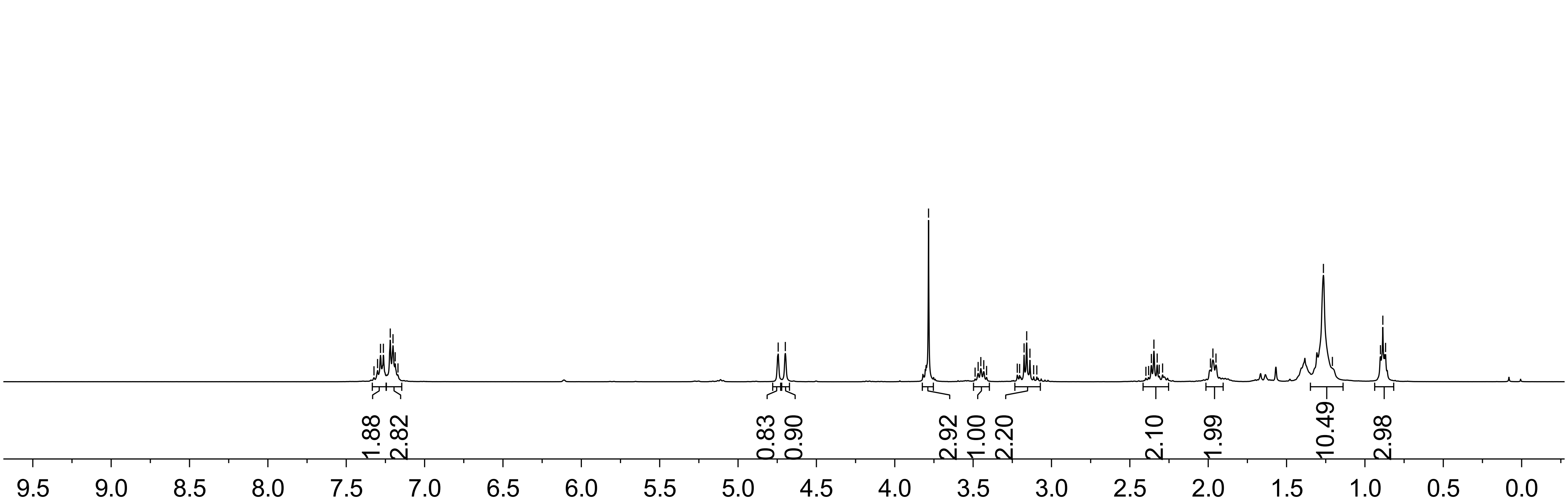

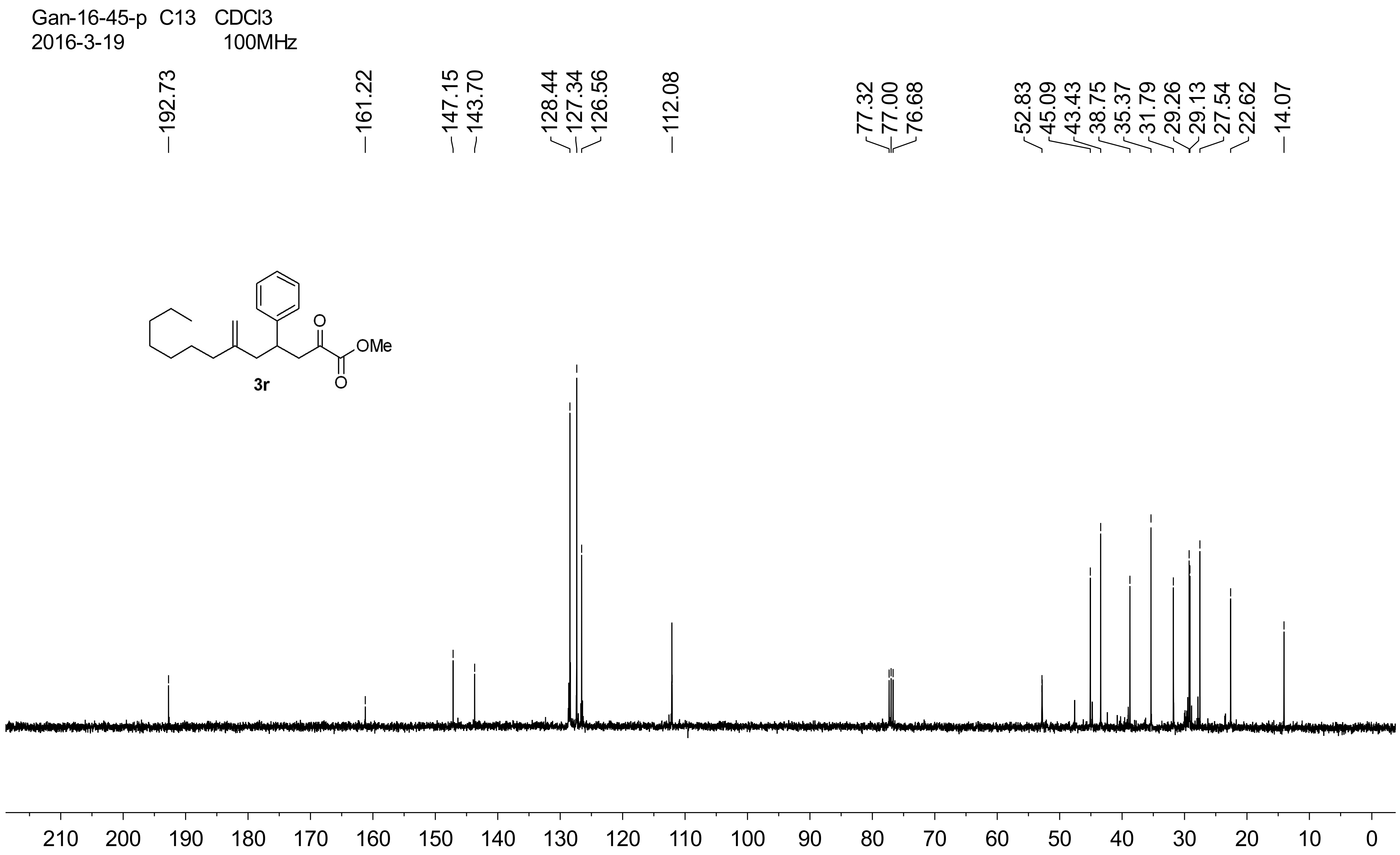

Gan-16-45-p C13 CDCl3
2016-3-19 100MHz
192.73
161.22
147.15
143.70
128.44
127.34
126.56
112.08
77.32
77.00
76.68
52.83
45.09
43.43
38.75
35.37
31.79
29.26
29.13
27.54
22.62
14.07
O
OMe
O
3r
210 200 190 180 170 160 150 140 130 120 110 100 90 80 70 60 50 40 30 20 10 0

Gan-16-35-1p H1 CDCl3
2016-3-10 100MHz

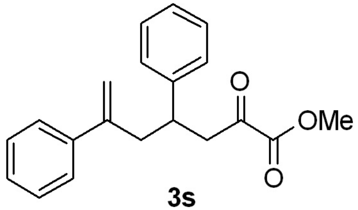

3s

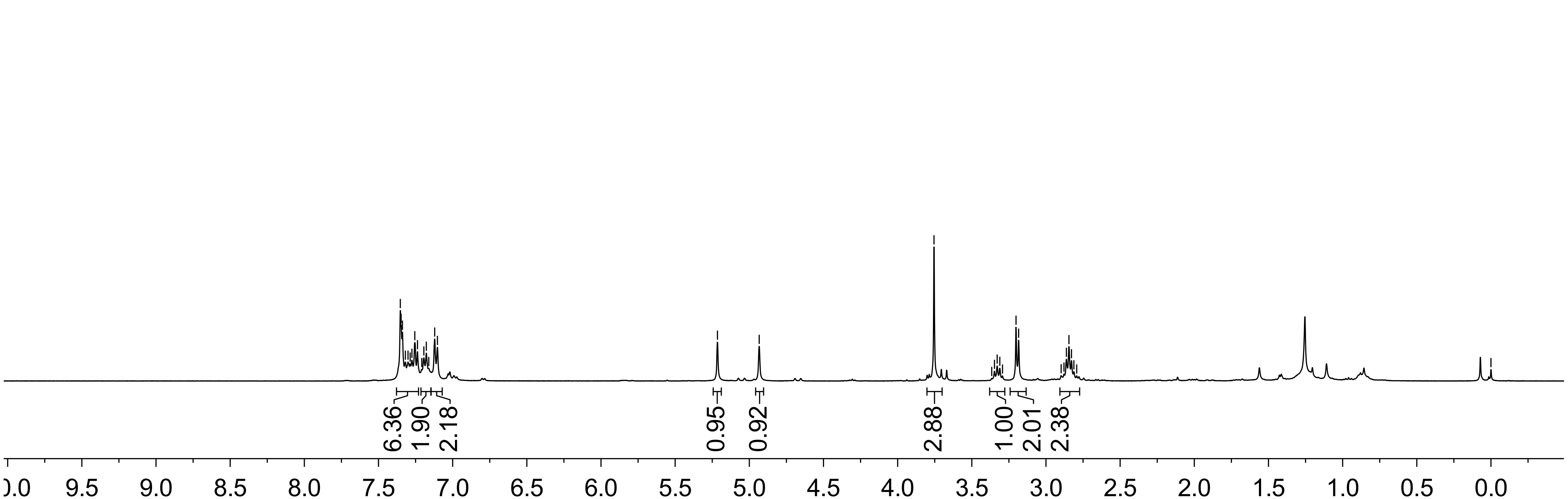

Gan-16-35-1p C13 CDCl3
2016-3-24 100MHz

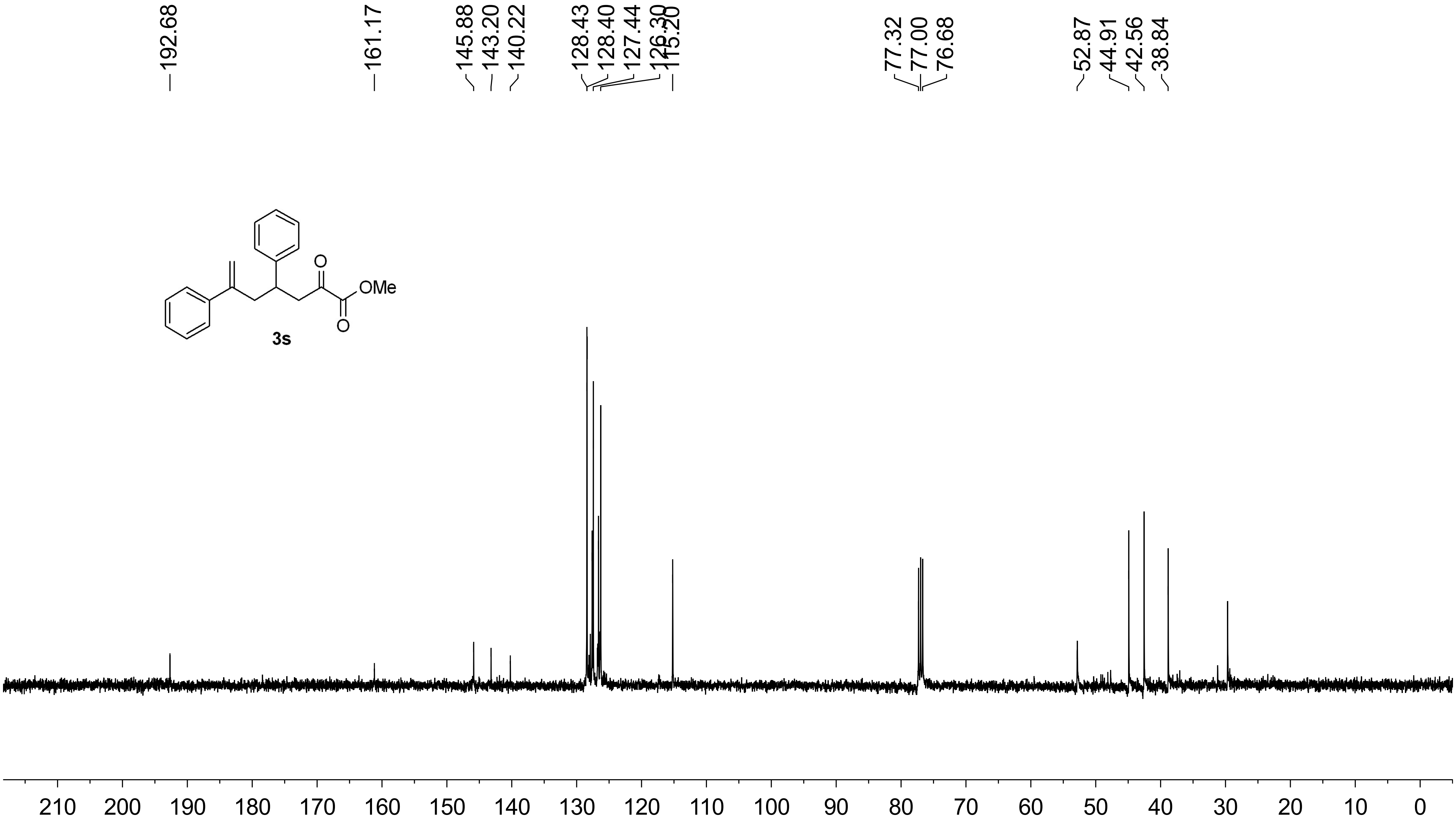

Gan-15-83-2p H1 CDCl3
2016-1-7 400MHz

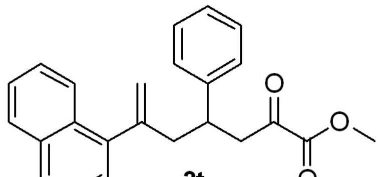

3t

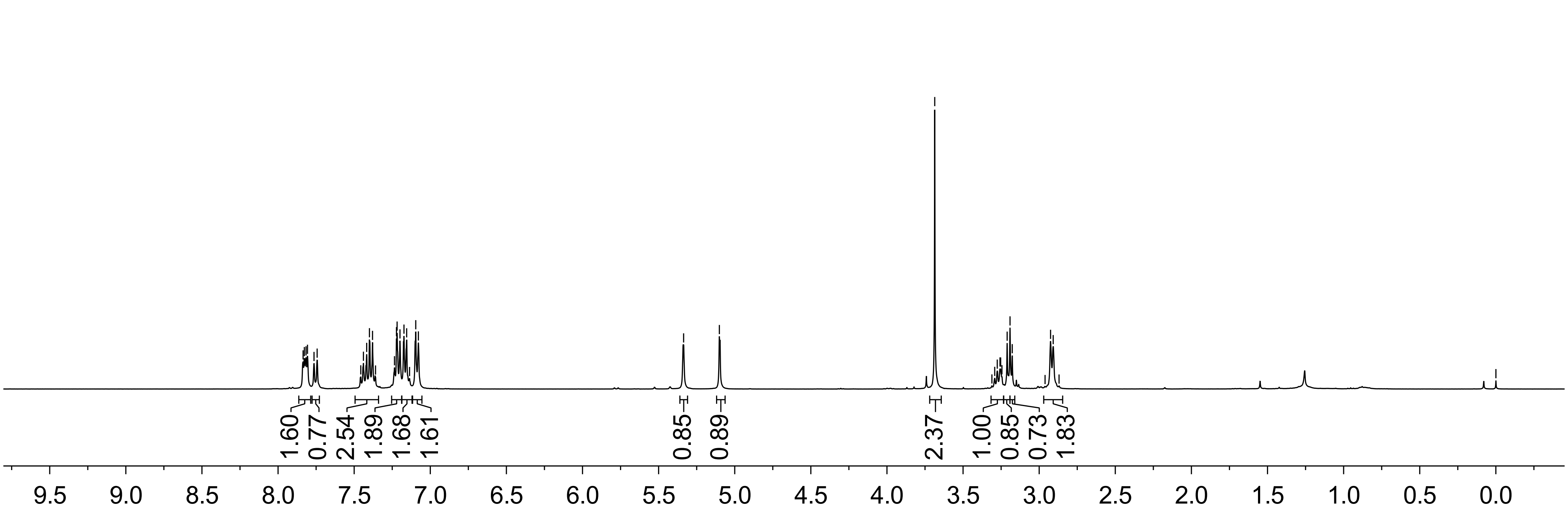

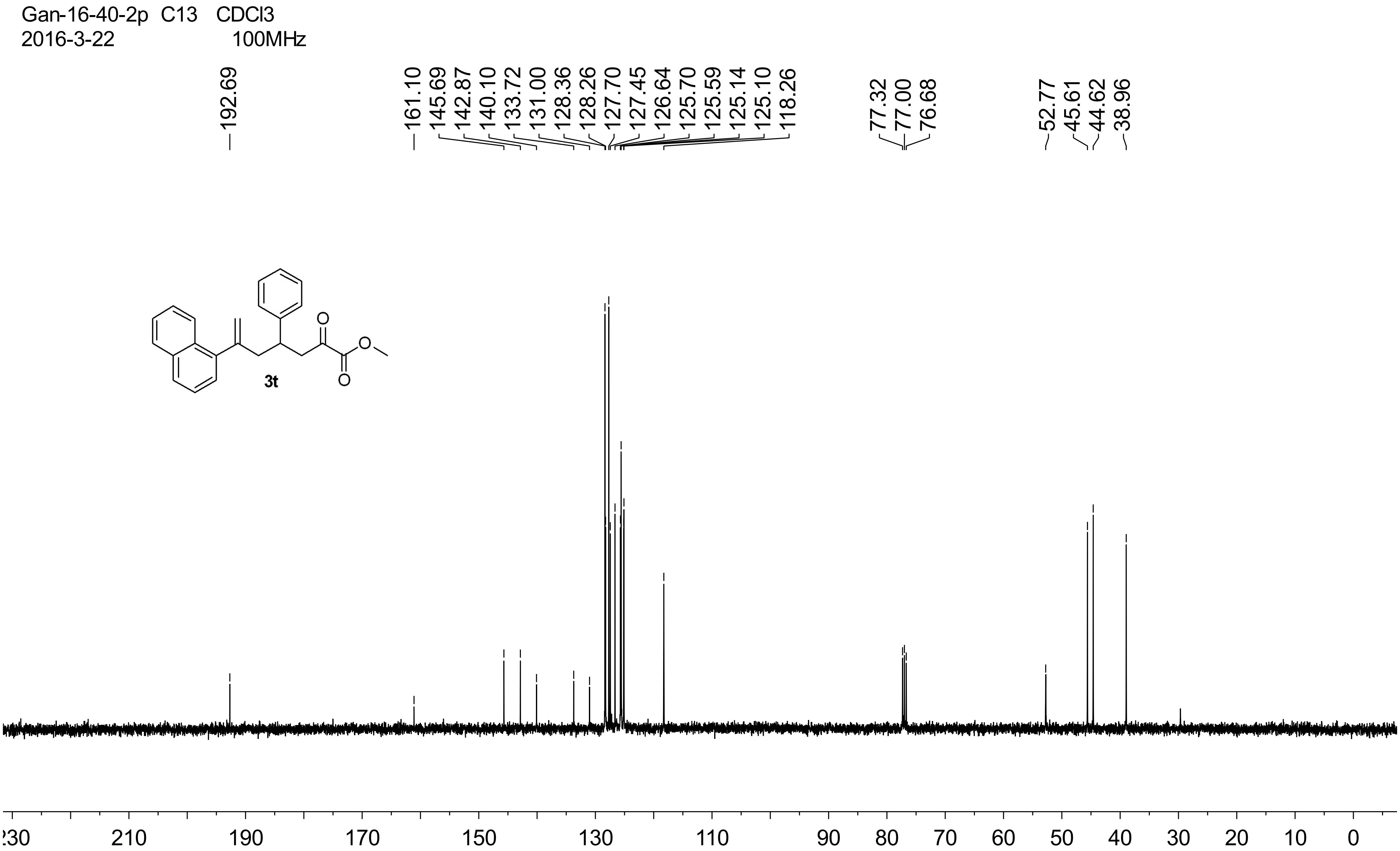
Gan-16-40-2p C13 CDCl3
2016-3-22 100MHz
192.69
161.10
145.69
142.87
140.10
133.72
131.00
128.36
128.26
127.70
127.45
126.64
125.70
125.59
125.14
125.10
118.26
77.32
77.00
76.68
52.77
45.61
44.62
38.96
O
O
O
3t
230
210
190
170
150
130
110
90
80
70
60
50
40
30
20
10
0

Gan-15-47-1p H1 CDCl3
2015-12-25 400MHz

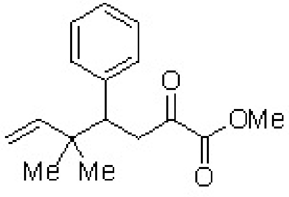

3u

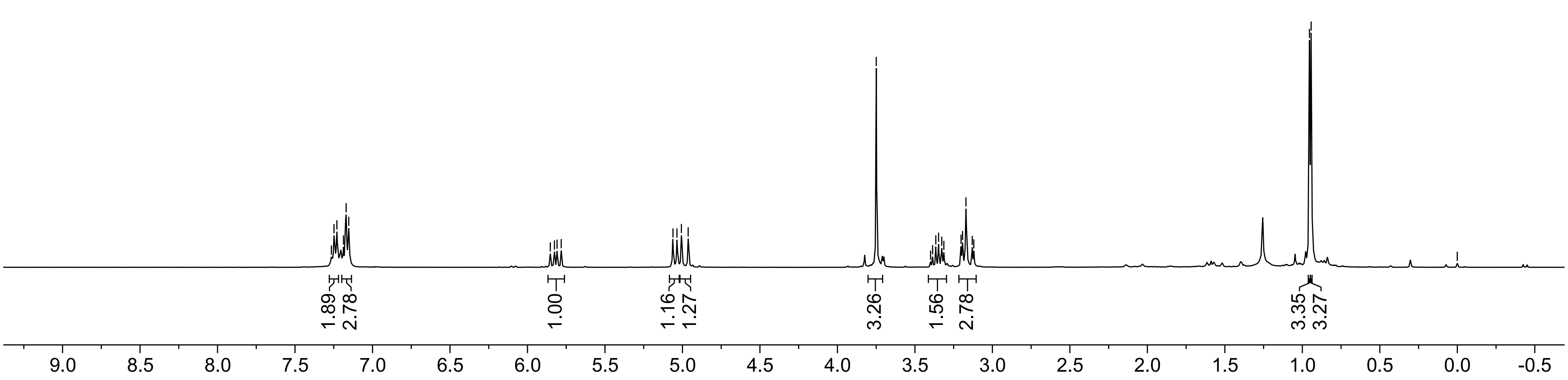

Gan-15-47-1p C13 CDCl3
2015-12-27 100MHz

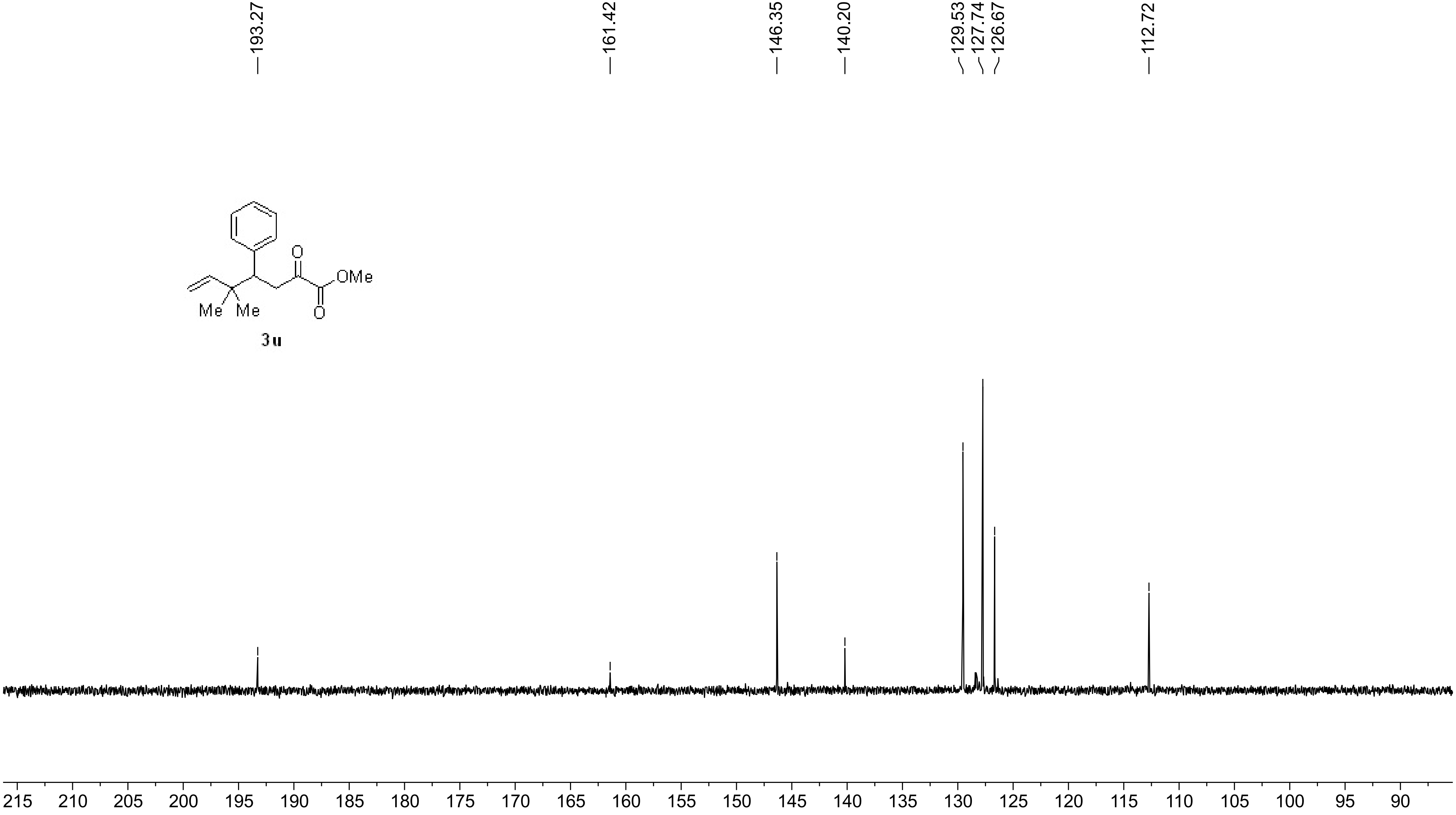

Gan-15-47-1p H1 CDCl3
2016-3-14 400MHz

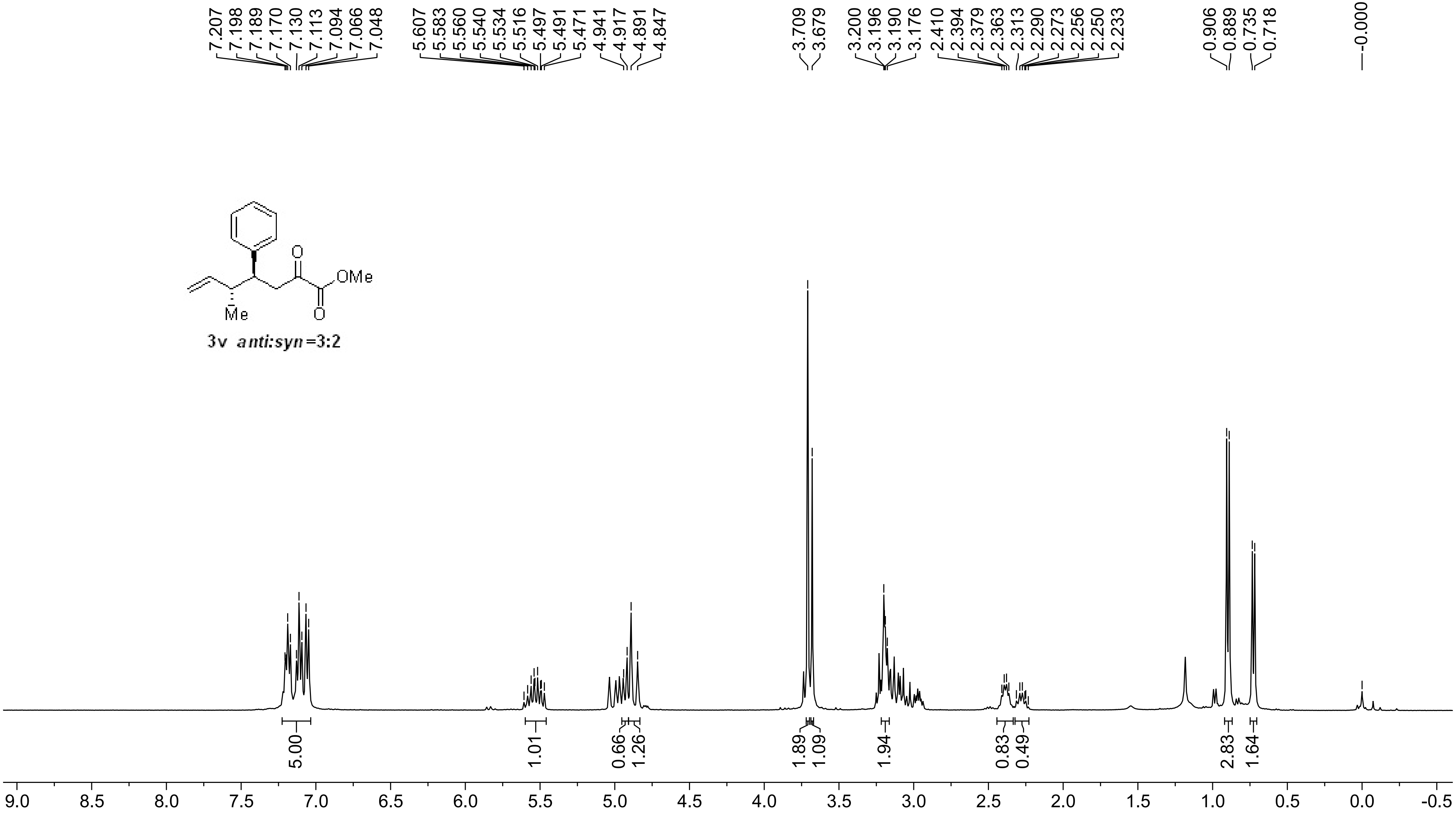

HLY-1-52-1p H1 CDCl3
2019-3-18 400MHz

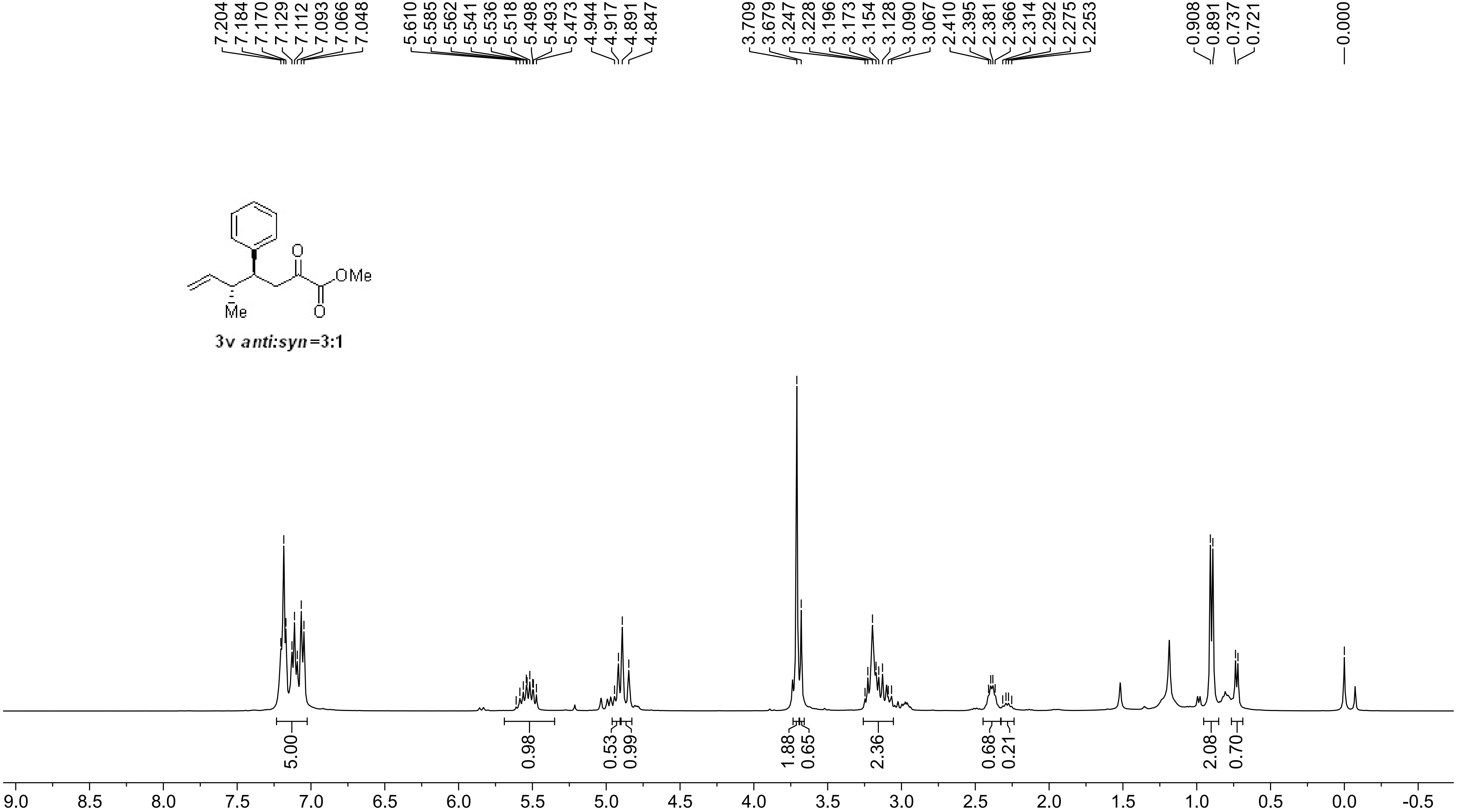

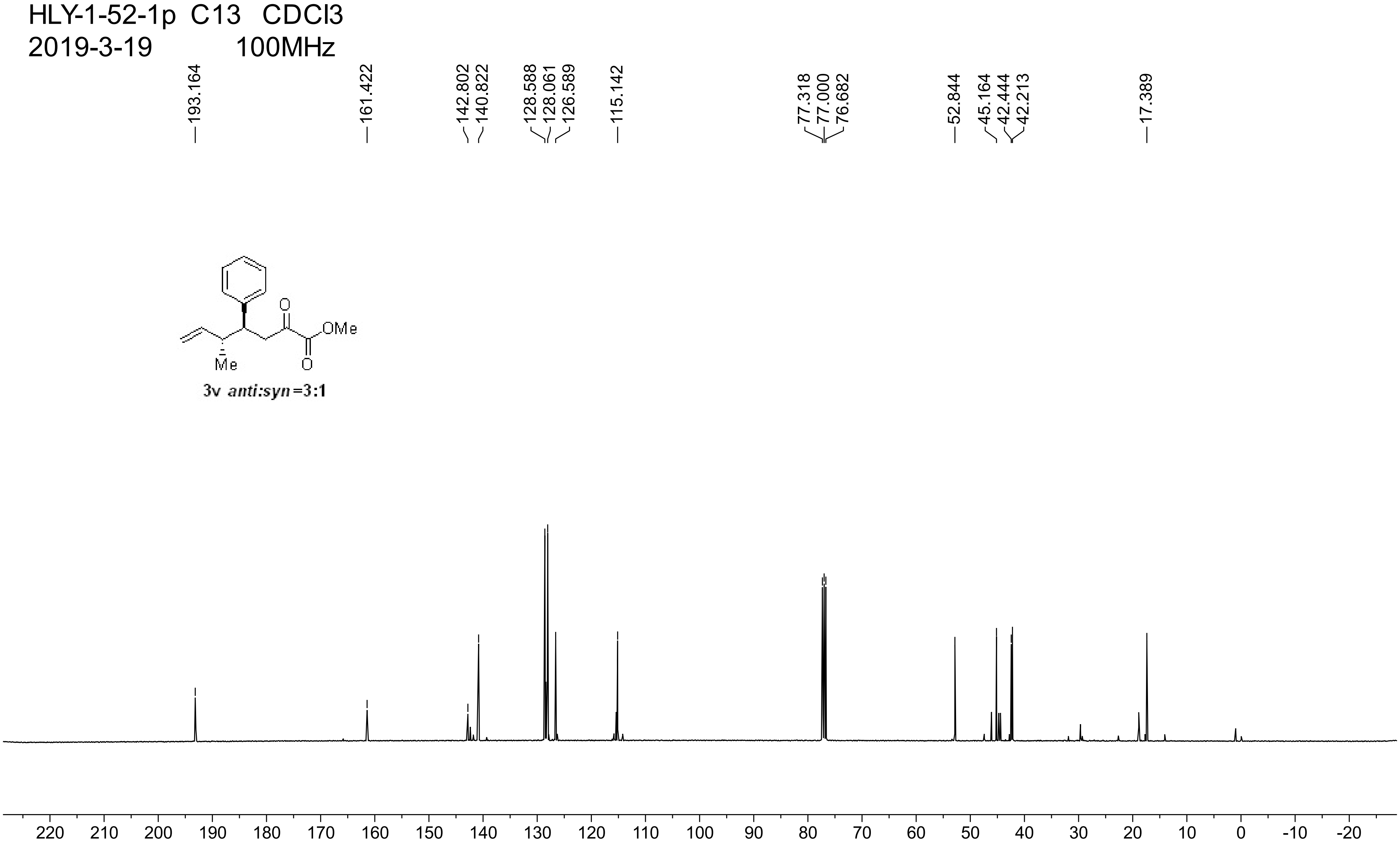
HLY-1-52-1p C13 CDCl3
2019-3-19 100MHz
193.164
161.422
142.802
140.822
128.588
128.061
126.589
115.142
77.318
77.000
76.682
52.844
45.164
42.444
42.213
17.389
OMe
Me
O
O
3v anti:syn=3:1
220 210 200 190 180 170 160 150 140 130 120 110 100 90 80 70 60 50 40 30 20 10 0 -10 -20

Gan-15-22-2p H1 CDCl3
2016-1-6 600MHz

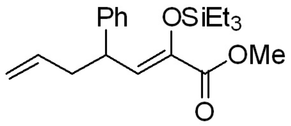

5a

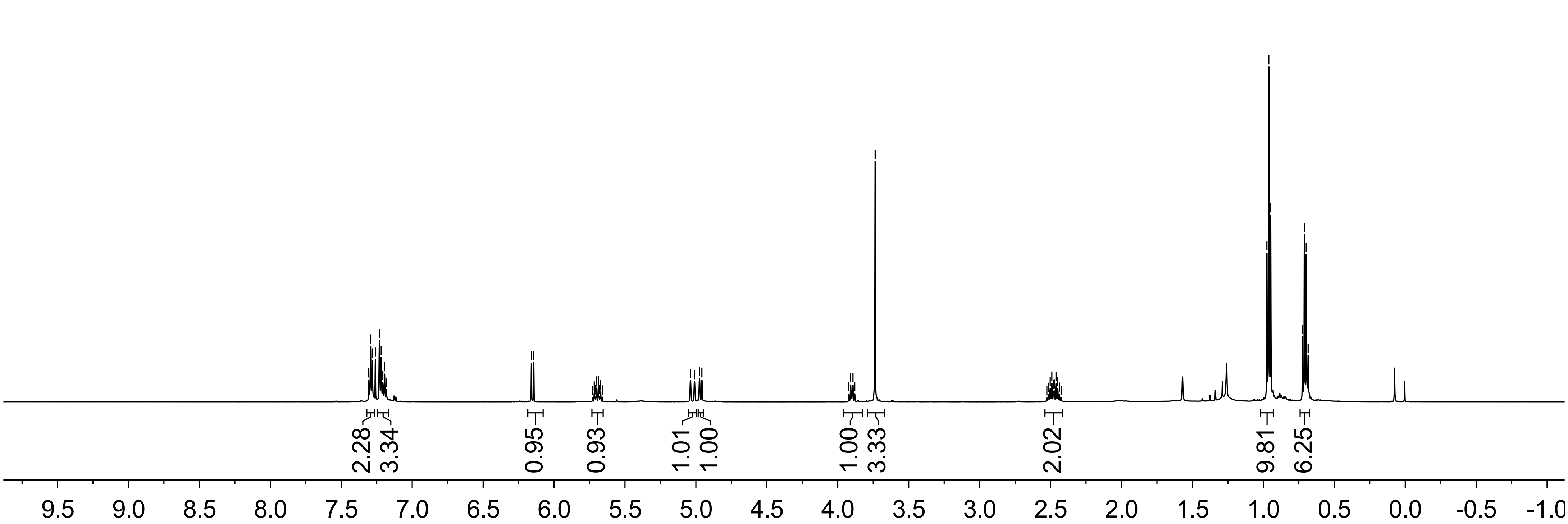

Gan-14-80-1p C13 CDCl3
2015-11-27 400MHz

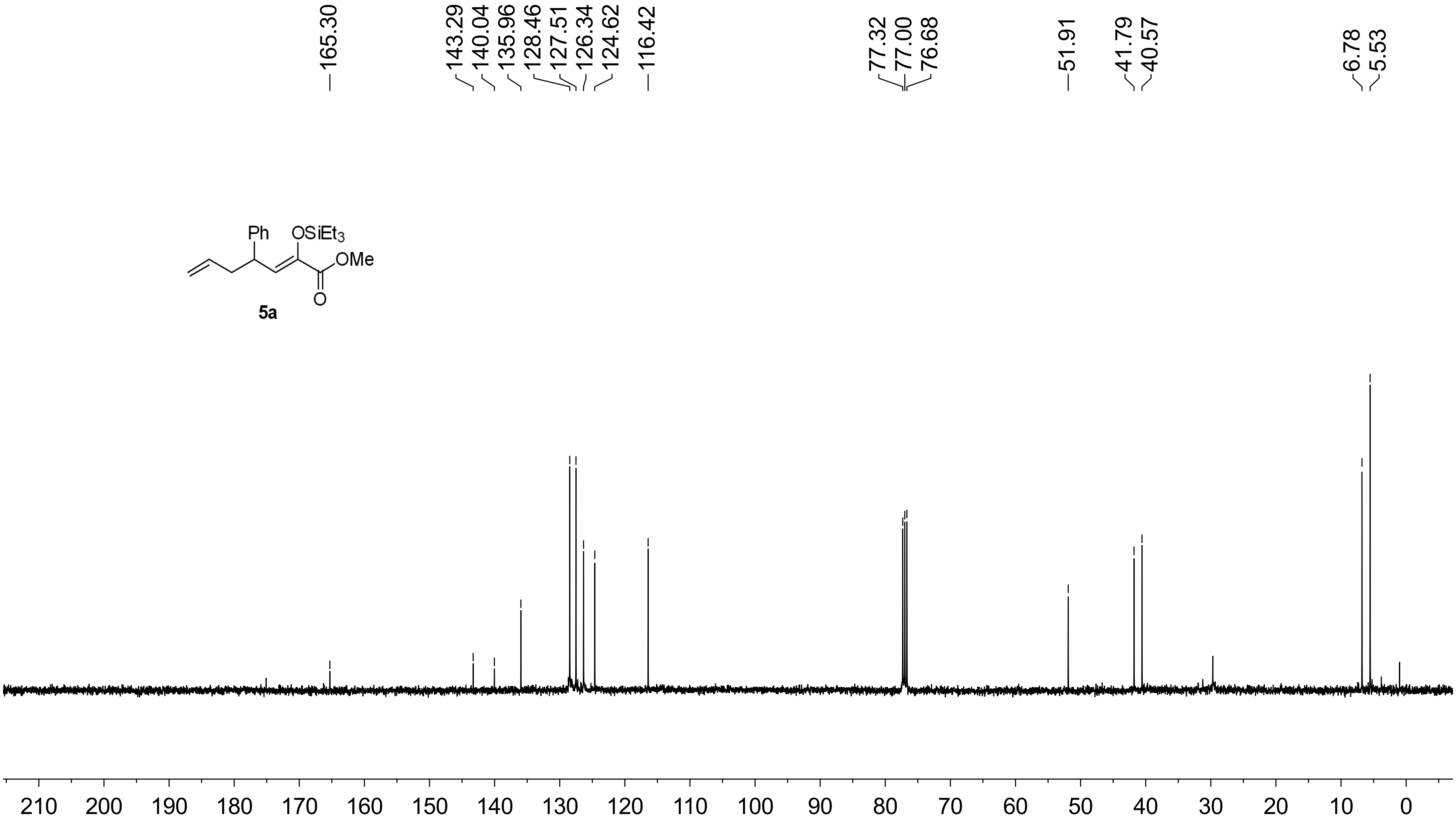

Gan-15-37-2p H1 CDCl3
2016-1-6 600MHz

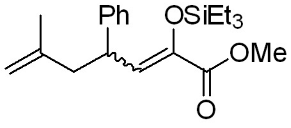

5b (*Z*:*E* = 2.5;1)

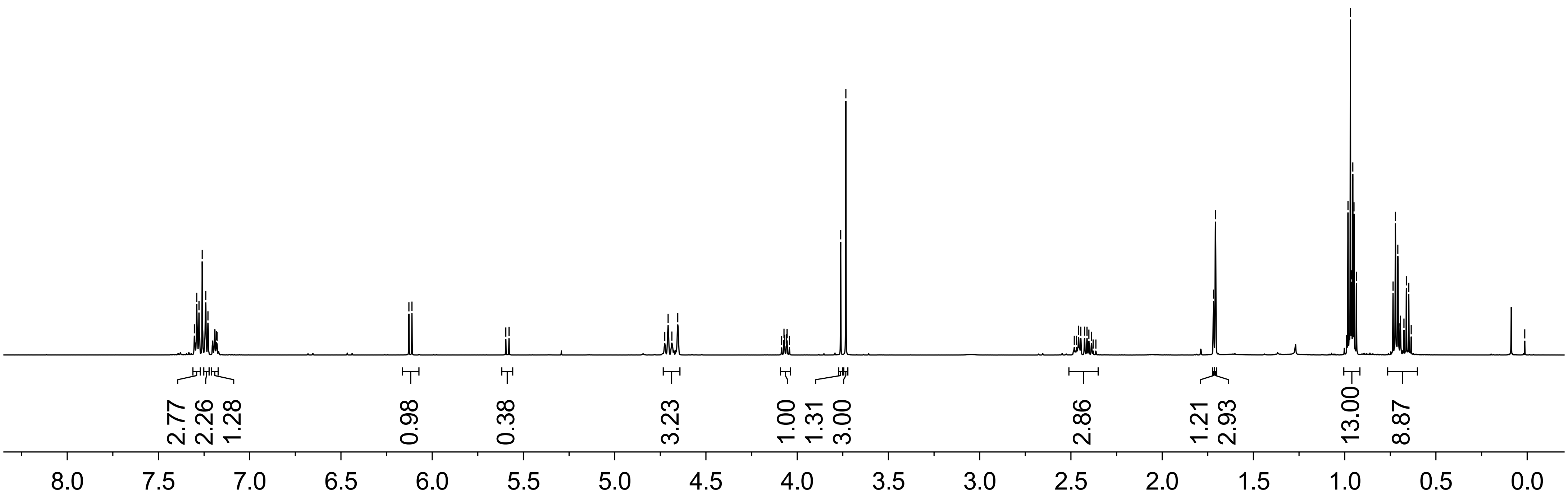

Gan-15-37-2p CDCl3
2015-12-24 400MHz

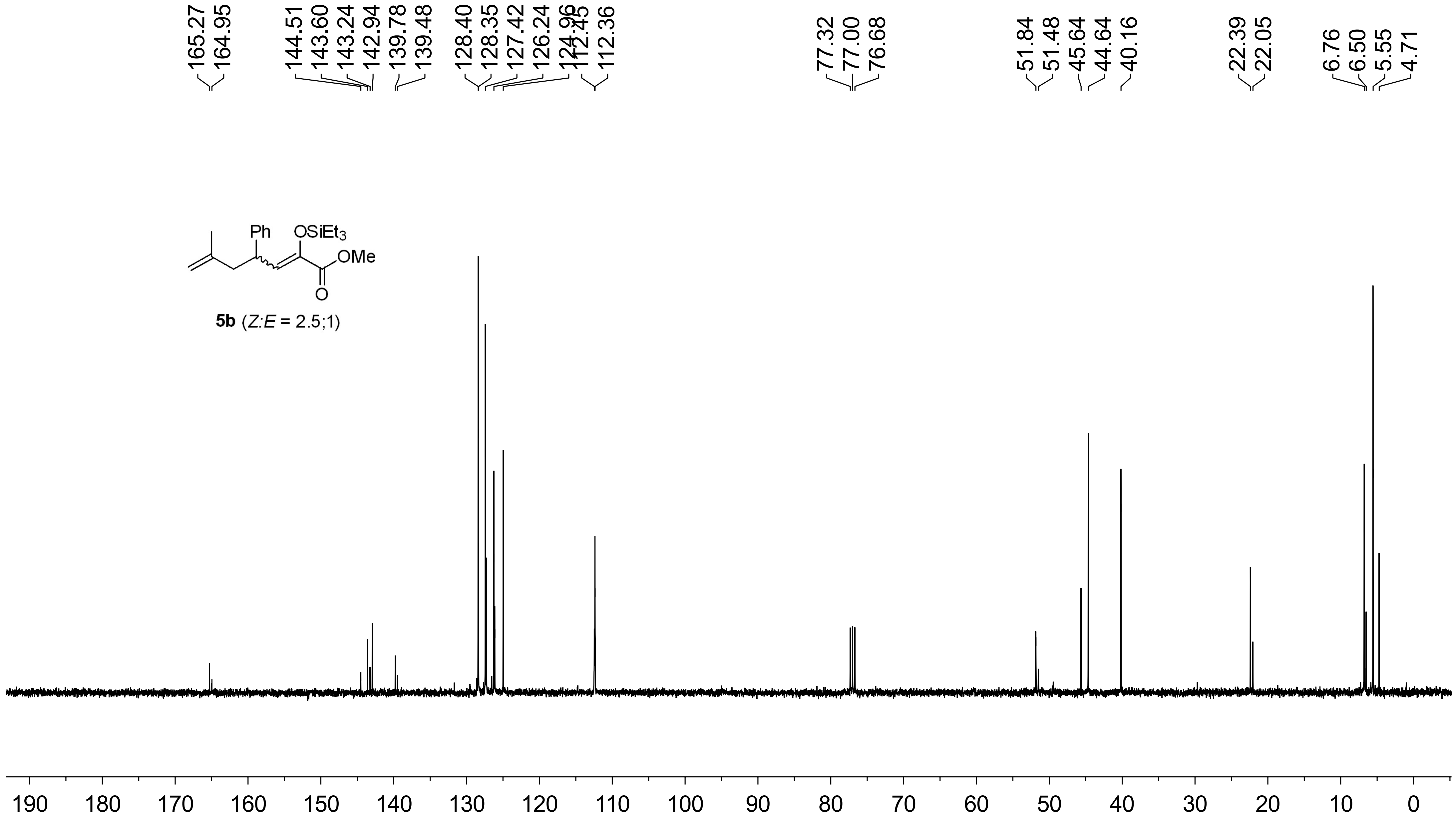

5b (*Z*:*E* = 2.5;1)

Gan-15-65-2p H1 CDCl3
2016-1-11 400MHz

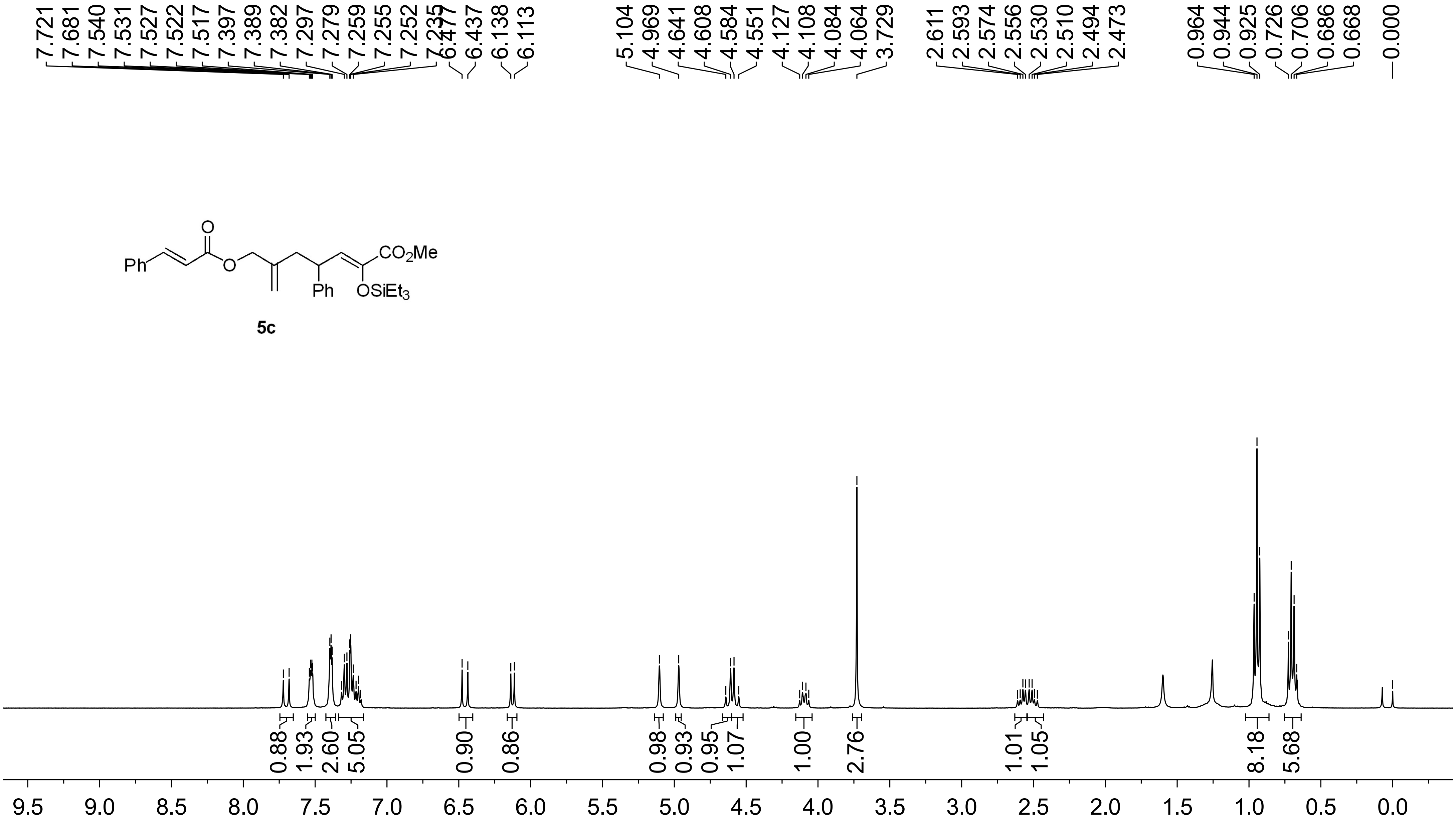

Gan-15-65-2p C13 CDCl3
2016-1-15 100MHz

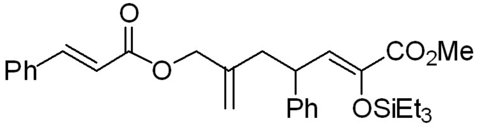

5c

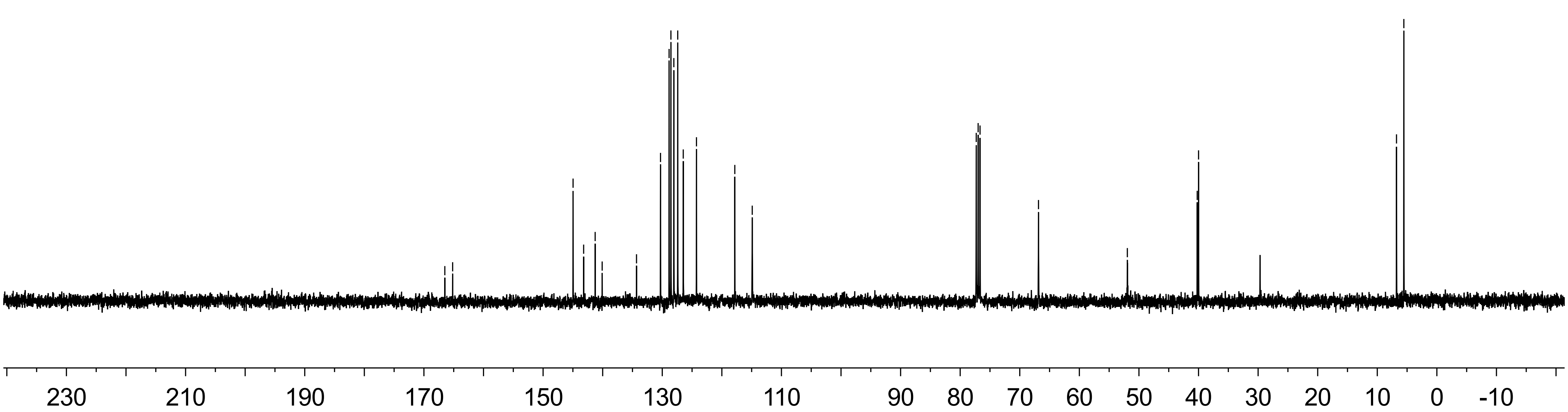

Gan-15-86-2p0 H1 CDCl3
2016-1-6 600MHz

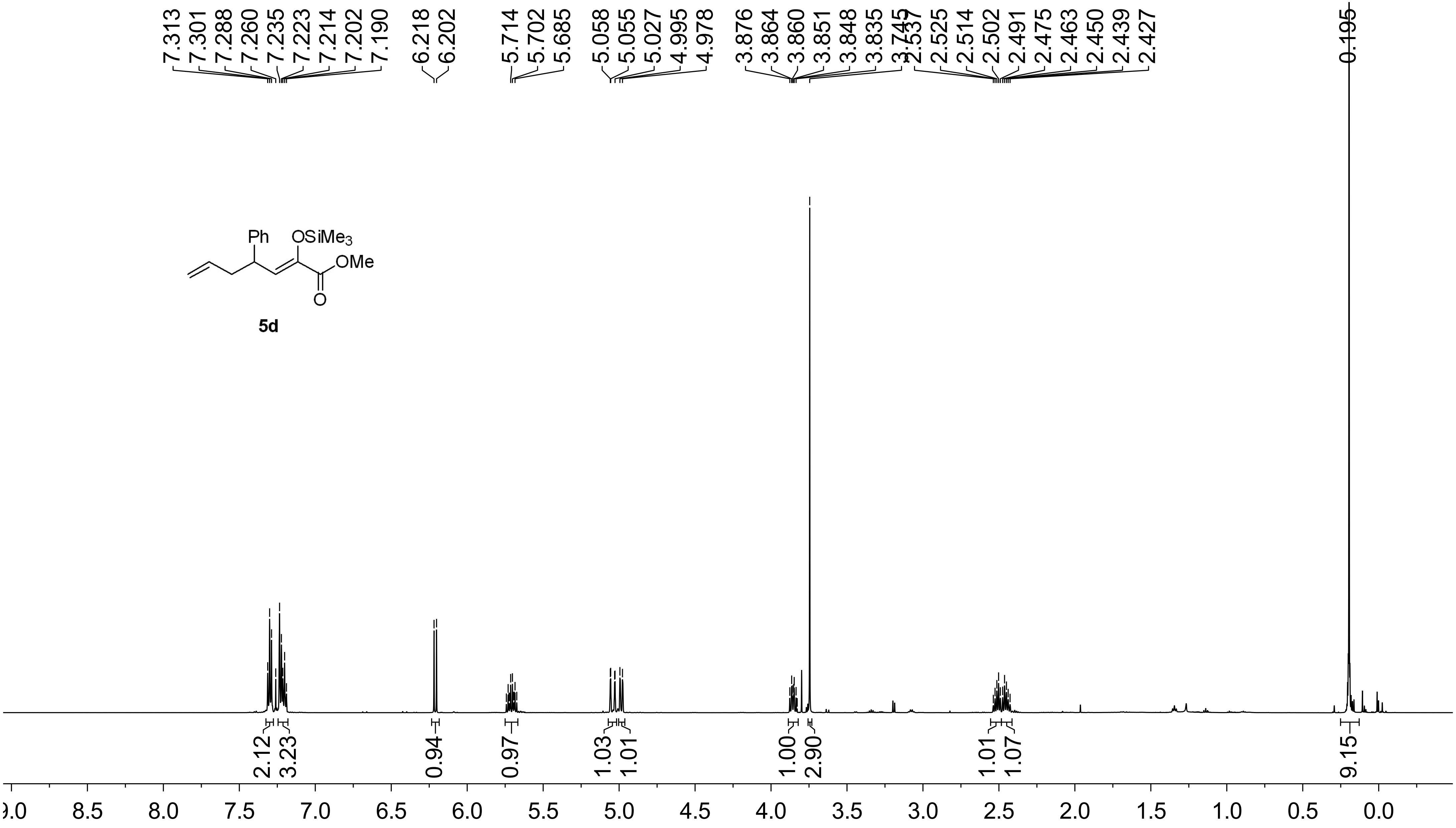

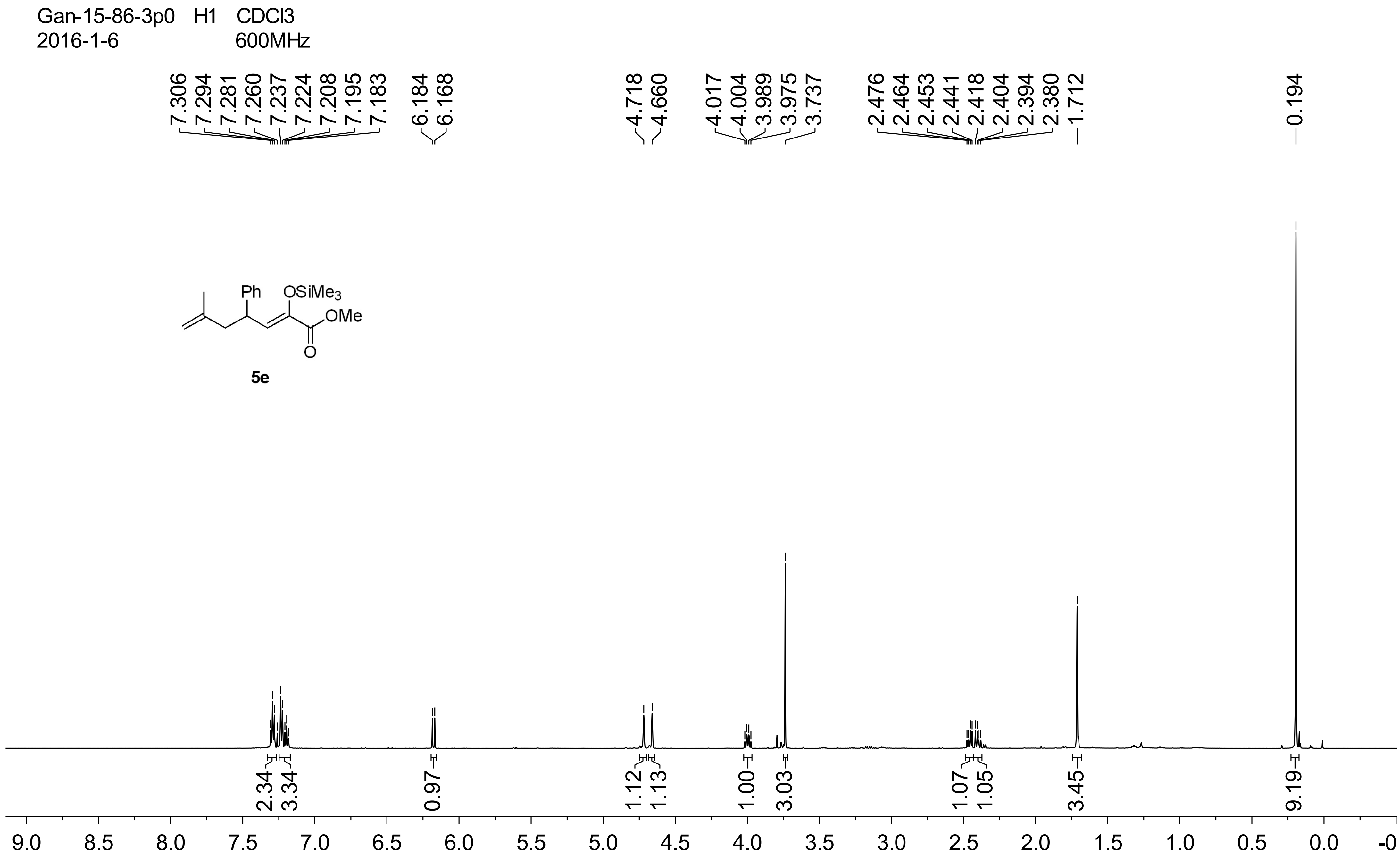
Gan-15-86-3p0 H1 CDCl3
2016-1-6 600MHz
7.306
7.294
7.281
7.260
7.237
7.224
7.208
7.195
7.183
6.184
6.168
4.718
4.660
4.017
4.004
3.989
3.975
3.737
2.476
2.464
2.453
2.441
2.418
2.404
2.394
2.380
1.712
0.194
Ph
OSiMe3
OMe
O
5e
2.34
3.34
0.97
1.12
1.13
1.00
3.03
1.07
1.05
3.45
9.19
9.0 8.5 8.0 7.5 7.0 6.5 6.0 5.5 5.0 4.5 4.0 3.5 3.0 2.5 2.0 1.5 1.0 0.5 0.0 -0

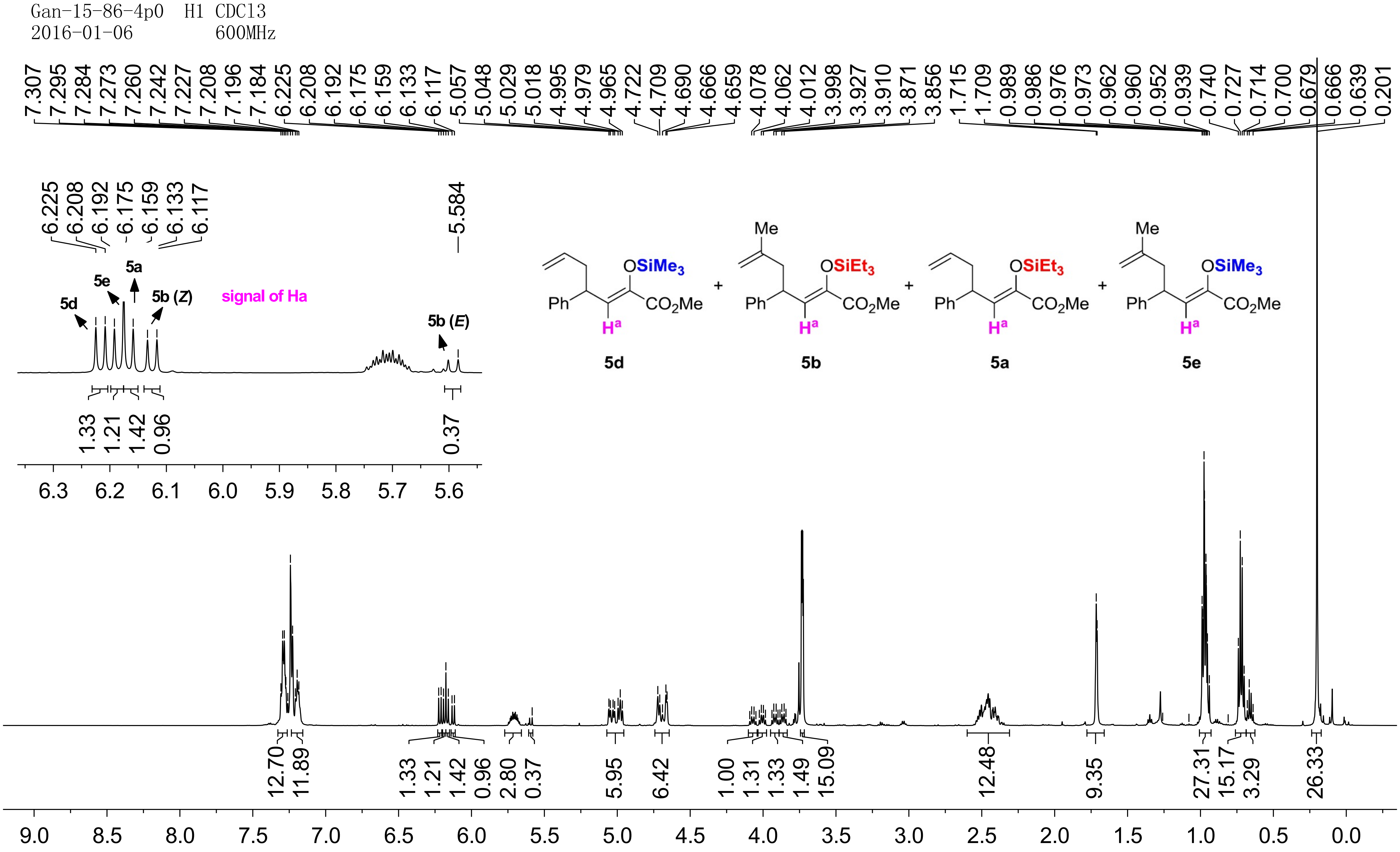
Gan-15-86-4p0 H1 CDCl3
2016-01-06 600MHz
7.307 7.295 7.284 7.273 7.260 7.242 7.227 7.208 7.196 7.184 6.225 6.208 6.192 6.175 6.159 6.133 6.117 5.057 5.048 5.029 5.018 4.995 4.979 4.965 4.722 4.709 4.690 4.666 4.659 4.078 4.062 4.012 3.998 3.927 3.910 3.871 3.856 1.715 1.709 0.989 0.986 0.976 0.973 0.962 0.960 0.952 0.939 0.740 0.727 0.714 0.700 0.679 0.666 0.639 0.201
6.225 6.208 6.192 6.175 6.159 6.133 6.117
5.584
5d
5e
5a
5b (Z)
5b (E)
signal of Ha
1.33 1.21 1.42 0.96
0.37
6.3 6.2 6.1 6.0 5.9 5.8 5.7 5.6
OSiMe3
Ph
CO2Me
Ha
5d
Me
OSiEt3
5b
5a
5e
12.70 11.89 1.33 1.21 1.42 0.96 2.80 0.37 5.95 6.42 1.00 1.31 1.33 1.49 15.09 12.48 9.35 27.31 15.17 3.29 26.33
9.0 8.5 8.0 7.5 7.0 6.5 6.0 5.5 5.0 4.5 4.0 3.5 3.0 2.5 2.0 1.5 1.0 0.5 0.0

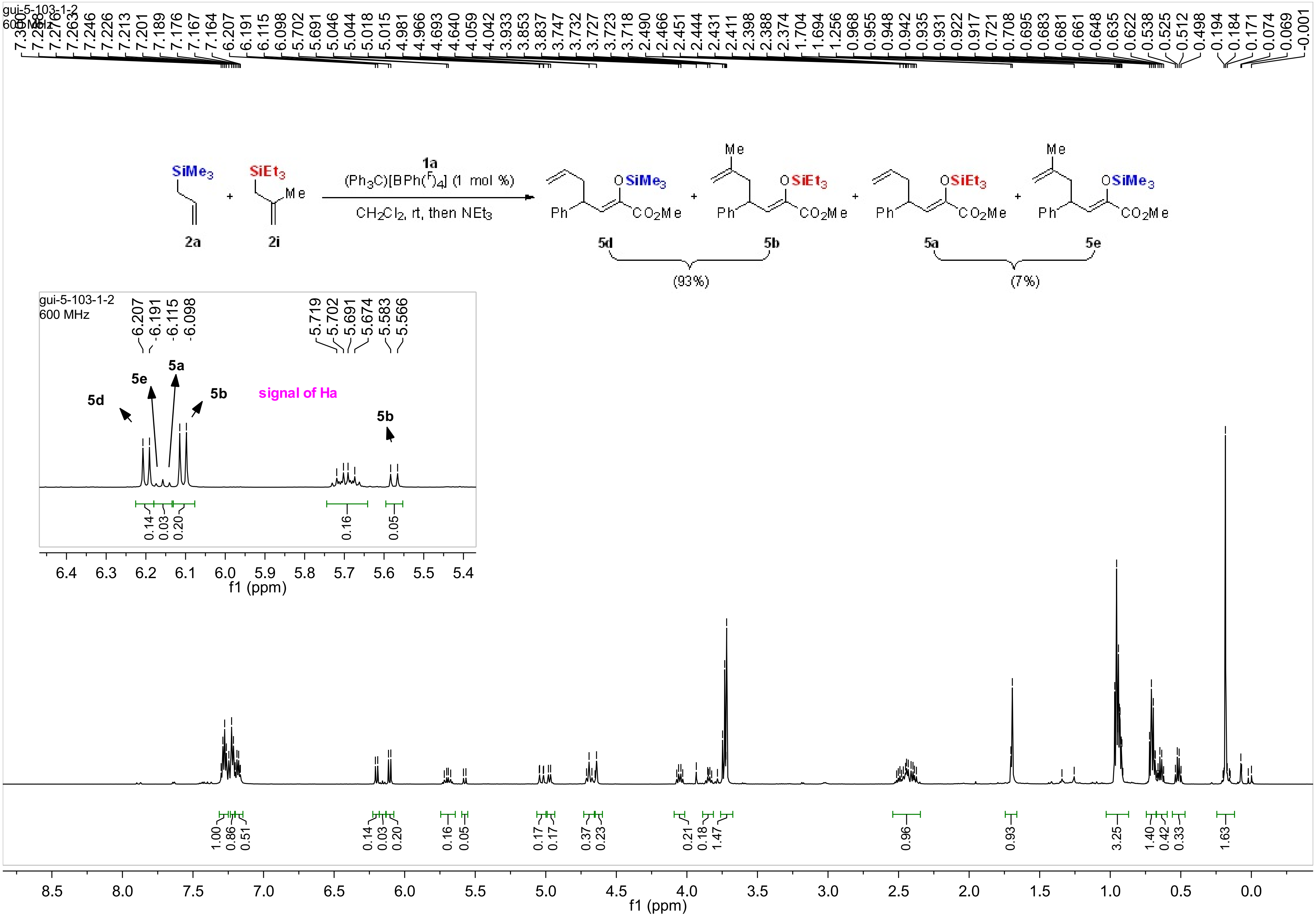
gui-5-103-1-2
600 MHz
1a
(Ph3C)[BPh(F)4] (1 mol %)
CH2Cl2, rt, then NEt3
2a
2i
5d
5b
5a
5e
(93%)
(7%)
signal of Ha
f1 (ppm)

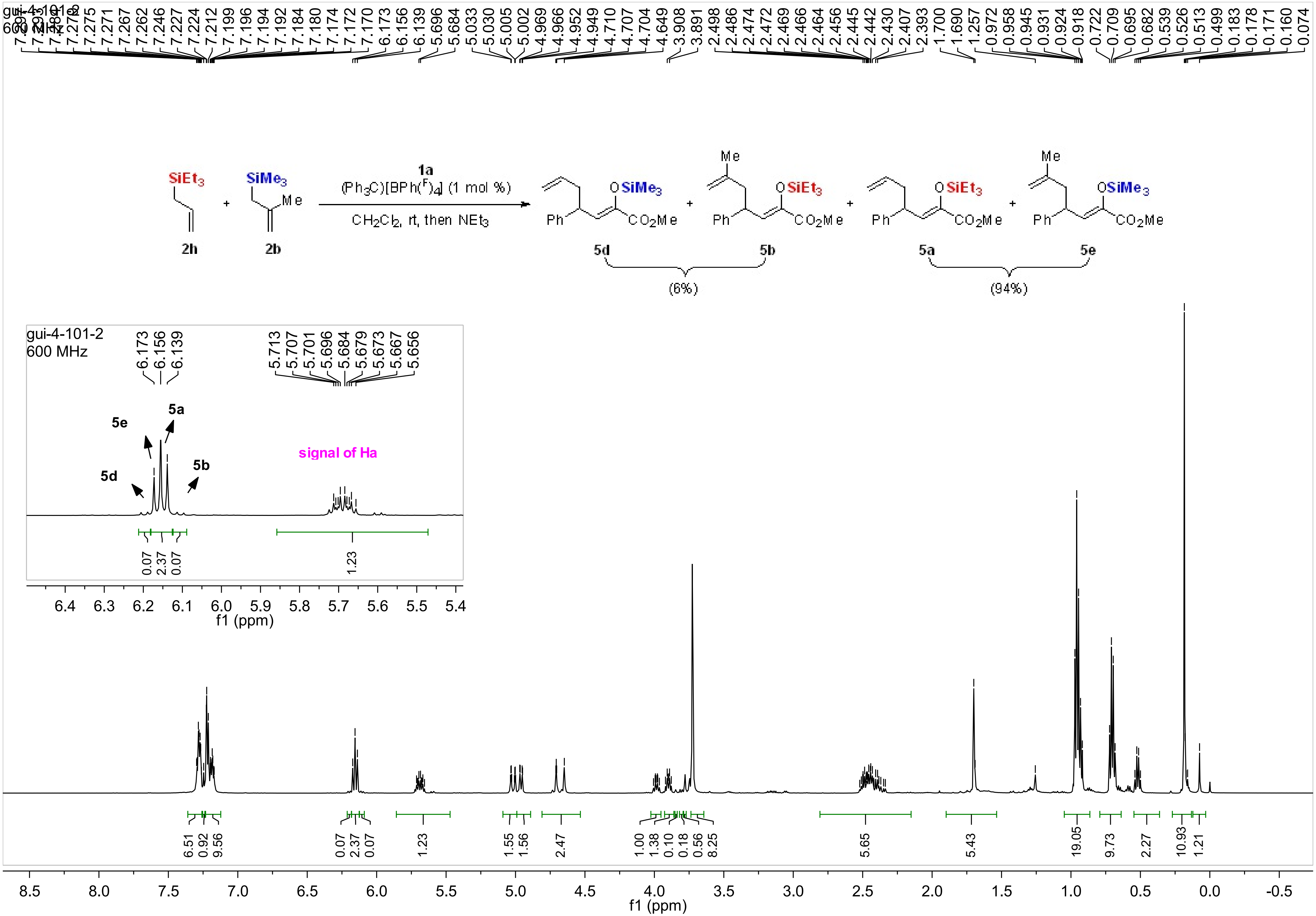

gui-4-101-2
600 MHz
7.275 7.271 7.267 7.262 7.246 7.227 7.224 7.212 7.199 7.196 7.194 7.192 7.184 7.180 7.174 7.172 7.170 6.173 6.156 6.139 5.696 5.684 5.033 5.030 5.005 5.002 4.969 4.966 4.952 4.949 4.710 4.707 4.704 4.649 3.908 3.891 2.498 2.486 2.474 2.472 2.469 2.466 2.464 2.456 2.445 2.442 2.430 2.407 2.393 1.700 1.690 1.257 0.972 0.958 0.945 0.931 0.924 0.918 0.722 0.709 0.695 0.682 0.539 0.526 0.513 0.499 0.183 0.178 0.171 0.160 0.074
SiEt3
SiMe3
Me
2h
2b
1a
(Ph3C)[BPh(F)4] (1 mol %)
CH2Cl2, rt, then NEt3
OSiMe3
OSiEt3
Ph
CO2Me
5d
5b
5a
5e
(6%)
(94%)
gui-4-101-2
600 MHz
6.173 6.156 6.139
5.713 5.707 5.701 5.696 5.684 5.679 5.673 5.667 5.656
signal of Ha
0.07 2.37 0.07
1.23
6.4 6.3 6.2 6.1 6.0 5.9 5.8 5.7 5.6 5.5 5.4
f1 (ppm)
6.51 0.92 9.56
0.07 2.37 0.07
1.23
1.55 1.56
2.47
1.00 1.38 0.10 0.18 0.56 8.25
5.65
5.43
19.05
9.73
2.27
10.93 1.21
8.5 8.0 7.5 7.0 6.5 6.0 5.5 5.0 4.5 4.0 3.5 3.0 2.5 2.0 1.5 1.0 0.5 0.0 -0.5
f1 (ppm)

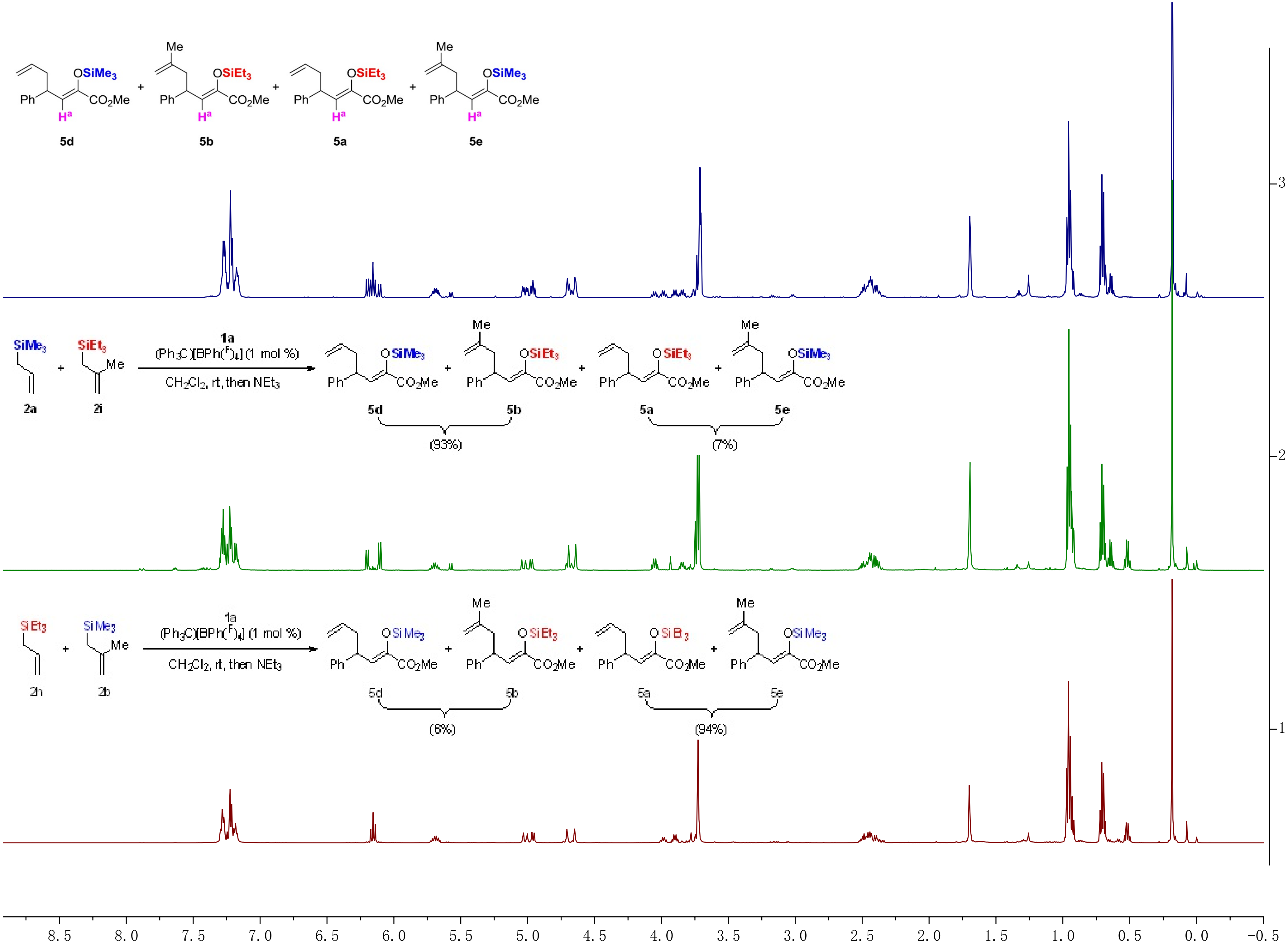

OSiMe3
Ph
CO2Me
Ha
5d
Me
OSiEt3
5b
5a
5e
SiMe3
SiEt3
Me
2a
2i
1a
(Ph3C)[BPh(F)4] (1 mol %)
CH2Cl2, rt, then NEt3
(93%)
(7%)
2h
2b
(6%)
(94%)
3
2
1
8.5
8.0
7.5
7.0
6.5
6.0
5.5
5.0
4.5
4.0
3.5
3.0
2.5
2.0
1.5
1.0
0.5
0.0
-0.5
